# Systematic review of empiric studies on lockdowns, workplace closures, and other non-pharmaceutical interventions in non-healthcare workplaces during the initial year of the COVID-19 pandemic: benefits and selected unintended consequences

**DOI:** 10.1186/s12889-024-18377-1

**Published:** 2024-03-22

**Authors:** Faruque Ahmed, Livvy Shafer, Pallavi Malla, Roderick Hopkins, Sarah Moreland, Nicole Zviedrite, Amra Uzicanin

**Affiliations:** 1 Division of Global Migration Health, Centers for Disease Control and Prevention, 1600 Clifton Road NE, Mailstop V18-2, Atlanta, GA 30329-4027 USA; 2 Oak Ridge Institute for Science and Education, Oak Ridge, TN USA; 3 Cherokee Nation Operational Solutions, Tulsa, OK USA

**Keywords:** Anxiety, Community mitigation, COVID-19, Depression, Employment, Lockdown, Non-pharmaceutical, Novel coronavirus, Social distancing, Systematic review, Workplace

## Abstract

**Background:**

We conducted a systematic review aimed to evaluate the effects of non-pharmaceutical interventions within non-healthcare workplaces and community-level workplace closures and lockdowns on COVID-19 morbidity and mortality, selected mental disorders, and employment outcomes in workers or the general population.

**Methods:**

The inclusion criteria included randomized controlled trials and non-randomized studies of interventions. The exclusion criteria included modeling studies. Electronic searches were conducted using MEDLINE, Embase, and other databases from January 1, 2020, through May 11, 2021. Risk of bias was assessed using the Risk of Bias in Non-Randomized Studies of Interventions (ROBINS-I) tool. Meta-analysis and sign tests were performed.

**Results:**

A total of 60 observational studies met the inclusion criteria. There were 40 studies on COVID-19 outcomes, 15 on anxiety and depression symptoms, and five on unemployment and labor force participation. There was a paucity of studies on physical distancing, physical barriers, and symptom and temperature screening within workplaces. The sign test indicated that lockdown reduced COVID-19 incidence or case growth rate (23 studies, *p* < 0.001), reproduction number (11 studies, *p* < 0.001), and COVID-19 mortality or death growth rate (seven studies, *p* < 0.05) in the general population. Lockdown did not have any effect on anxiety symptoms (pooled standardized mean difference = -0.02, 95% CI: -0.06, 0.02). Lockdown had a small effect on increasing depression symptoms (pooled standardized mean difference = 0.16, 95% CI: 0.10, 0.21), but publication bias could account for the observed effect. Lockdown increased unemployment (pooled mean difference = 4.48 percentage points, 95% CI: 1.79, 7.17) and decreased labor force participation (pooled mean difference = -2.46 percentage points, 95% CI: -3.16, -1.77). The risk of bias for most of the studies on COVID-19 or employment outcomes was moderate or serious. The risk of bias for the studies on anxiety or depression symptoms was serious or critical.

**Conclusions:**

Empiric studies indicated that lockdown reduced the impact of COVID-19, but that it had notable unwanted effects. There is a pronounced paucity of studies on the effect of interventions within still-open workplaces. It is important for countries that implement lockdown in future pandemics to consider strategies to mitigate these unintended consequences.

**Systematic review registration:**

PROSPERO registration # CRD42020182660.

**Supplementary Information:**

The online version contains supplementary material available at 10.1186/s12889-024-18377-1.

## Background

Coronavirus disease (COVID-19) is an infectious disease caused by Severe Acute Respiratory Syndrome Coronavirus 2 (SARS-CoV-2) that emerged in December 2019. COVID-19 has caused a global pandemic that resulted in long-term health problems as well as millions of deaths around the world [1]. The World Health Organization Director-General indicated that all countries must strike a fine balance between protecting health and minimizing economic and social disruption [2]. Several community-level containment and closure policies were implemented by government authorities to reduce the transmission of SARS-CoV-2 and avert overwhelming of healthcare systems. These policies included cancellation of public events, restrictions on gathering sizes, restrictions on internal movement and international travel, closure of public transport systems, school closures, closures of non-essential businesses, and lockdowns [3]. Governments provided fiscal support to varying extents to reduce financial hardship due to the COVID-19 pandemic and the interventions to reduce SARS-CoV-2 transmission [1, 4, 5].

About two-thirds of the global population over 15 years of age participate in the labor force [6]. SARS-CoV-2 transmission can occur in workplaces through respiratory droplets and aerosols generated by pre-symptomatic, asymptomatic, or symptomatic persons and through fomites [7, 8]. In 2020, employers were encouraged to implement several measures to prevent and reduce the transmission of SARS-CoV-2 within the workplace, including use of face masks or coverings, physical distancing to increase physical space between people and decrease the frequency of face-to-face contact (including teleworking), symptom and temperature screening, flexible leave policies to facilitate self-isolation of sick workers, environmental cleaning and disinfection, and engineering controls to improve air quality (Additional file 1: Appendix Table S1) [9–11]. These measures could be used by essential businesses that were not subject to government-mandated closures and by all businesses when lockdowns were not in effect.

Research has primarily focused on preventing or reducing SARS-CoV-2 infection in healthcare workers, with non-healthcare workers receiving less attention [12]. A Cochrane systematic review on interventions in non-healthcare workplaces examined the effect of interventions introduced by researchers [12]. The review identified one study that met their inclusion criteria, which was a cluster-randomized non-inferiority trial that assigned staff working in schools to standard isolation after contact with a SARS-CoV-2-infected person or to daily COVID-19 testing and staying at work if the test was negative. Because randomizing employers or geographic regions to workplace-related non-pharmaceutical interventions (NPIs) may not be feasible or ethical during an outbreak, observational studies may provide the best available evidence. We conducted a systematic review to assess the benefits and unintended consequences of NPIs in non-healthcare workplaces that included observational studies. The objectives of our review were to evaluate the effects of NPIs within non-healthcare workplaces and community-level workplace closures and lockdowns, compared to no intervention, on the following outcomes in workers or the general population: 1) COVID-19 morbidity and mortality, 2) selected mental disorders, and 3) employment outcomes.

## Methods

We registered our systematic review protocol on PROSPERO (ID # CRD42020182660) [13]. We reported the review according to the Preferred Reporting Items for Systematic Reviews and Meta-Analysis (PRISMA) statement (Appendix Table S2) [14].

### Protocol amendments

We amended our original protocol to exclude studies on severe acute respiratory syndrome (SARS) and Middle East respiratory syndrome (MERS). We included lockdown that affects workplaces and selected mental disorders. We excluded the following interventions: staying home when ill, respiratory etiquette, and cleaning and disinfection of frequently touched surfaces and objects. We excluded qualitative and modeling studies. We examined the references of relevant systematic reviews to identify studies that met our inclusion criteria instead of performing a systematic review of systematic reviews.

### Literature search strategy and study selection

Electronic searches of the published and grey literature were conducted using MEDLINE, Embase, PsycINFO, Cumulative Index to Nursing and Allied Health Literature (CINAHL), Scopus, Cochrane Library, NIOSHTIC-2, and EconLit to identify studies published in English from January 1, 2020, through May 11, 2021. The search strategy is provided in Appendix Table S3. Additional studies were identified through authors’ knowledge and examination of references of included studies and previous systematic reviews.

### Inclusion and exclusion criteria

The inclusion criteria included randomized controlled trials and non-randomized studies (cohort, case–control, before-after, controlled before-after, interrupted time series). Cohort studies include both inception cohorts and retrospective cohorts. Controlled before-after studies commonly present a ‘difference in differences’ analysis, where before-after differences in the outcome are compared between the intervention and comparator groups. Before-after and controlled before-after studies can include measurements on the same individual before and after the intervention, or on different individuals at each time point. Interrupted time series studies are those with at least three measurement times before the intervention and at least three measurement times after the intervention. More details about the study designs are available elsewhere [15].

The population of interest was persons working in non-healthcare settings, with no restrictions regarding age, sex, or race/ethnicity. We included the following NPIs within non-healthcare workplaces: 1) Physical distancing (e.g., increased use of telework, email, and teleconferences; increasing physical space between employees; modifying schedules for on-site work; staggered work hours; limiting customers in indoor spaces, including capacity restrictions and outdoor dining; increasing physical space between employees and customers, including delivering services remotely, drive-through service, curbside pick-up, or delivery); 2) Physical barriers (e.g., plexiglass partitions between workstations or at other points of close, frequent contact); 3) Symptom and/or temperature screening to prevent potentially infectious persons entering the job site. We also included community-level initial business closures (e.g., closing of restaurants, bars, and entertainment venues), closures of workplaces with exceptions for essential workers, and lockdowns. Lockdowns represent government mandates to stay home except for essential work or necessities and often include several but not necessarily all of the following in a geographic area: closure of non-essential businesses, restaurants and entertainment facilities; closure of schools and universities; prohibition of indoor and outdoor gatherings; restrictions on non-essential travel [16, 17]. Lockdowns are also called stay-at-home or shelter-in-place orders [18]. Persons may telework, if feasible, during workplace closures and lockdowns.

We assessed both public health benefits and selected unintended consequences of an intervention. The beneficiaries may be workers or the general population (including both working and non-working persons of any age). The benefits examined were reduction of COVID-19 morbidity and mortality: COVID-19 incidence, case growth rate, reproductive number, epidemic doubling time, COVID-19 mortality, death growth rate. COVID-19 incidence is defined as the number of new cases per 100,000 population, and COVID-19 mortality represents the number of COVID-19-attributed deaths per 100,000 population over a specified time period; the case or death growth rate is the percent increase/decrease in daily incidence of cases or deaths, respectively [19]. The reproduction number is the average number of secondary cases each current case would produce, and the epidemic doubling time is the number of days required for the daily incidence to double [19].

The unintended consequences assessed were anxiety and depression symptoms in workers or the general adult population (including both working and non-working persons), and unemployment and labor force participation rates in persons ages 16 years and older. Anxiety is characterized by excessive fear and worry and related behavioral disturbances [20], and depression is characterized by persistent sadness and a lack of interest or pleasure in previously rewarding or enjoyable activities [21]. The labor force participation rate is the number of people who are either employed or actively looking for work as a percentage of the civilian noninstitutional population aged 16 years and older [22]. The unemployment rate is the number of employed people as a percentage of the number of people who are employed or actively looking for work. People who are not actively looking for work are excluded from the denominator for computing the unemployment rate.

The exclusion criteria included the following: 1) Studies on SARS, MERS, influenza, influenza-like illness, or other diseases; 2) Editorials, commentaries, narrative reviews, as well as case series, cross-sectional, qualitative, and modeling studies; 3) Studies in healthcare, long-term care, nursing home, school, or university settings; 4) Studies on children, family members of healthcare workers or patients, or studies in animals; 5) Studies on hand hygiene, respiratory hygiene (including face mask or covering), generic physical distancing with no specific mention of workplace physical distancing, environmental cleaning and disinfection, isolation, quarantine, postponing work-related travel, or building engineering controls (e.g., ventilation, avoiding air recirculation, particle filtration, ultraviolet germicidal irradiation); 6) Studies that lacked a "no intervention" comparator; 7) Studies on mobility, workplace social contact rates, air pollution, access to health care (e.g., visits to physicians, cancer screening), mental disorders other than anxiety or depression (e.g., post-traumatic stress disorder), or employment outcomes other than unemployment and labor force participation (e.g., reduced work hours); 8) Publications in languages other than English.

### Data extraction and risk of bias assessment

Seven reviewers independently performed title and abstract screening, full text reviews, and data extraction using Covidence software, with each record reviewed by two persons [23]. The variables for which data were extracted included the following: country, population, source of outcome data, sample size, period of data collection, intervention, comparator, outcomes (COVID-19 incidence or case growth rate, epidemic doubling time, reproduction number, COVID-19 mortality or death growth rate, anxiety symptoms, depression symptoms, unemployment, labor force participation), study design, and funding source. Any disagreements were resolved through discussion or by a third reviewer. All risk of bias assessments were reviewed by a senior author. Study investigators were not contacted.

We did not identify any eligible randomized controlled trial. The quality of observational studies was assessed using the Risk of Bias in Non-Randomized Studies of Interventions (ROBINS-I) tool, which assesses the risk of bias of non-randomized studies compared to a well-performed randomized trial [15, 24]. Our effect of interest was assignment to intervention as opposed to adherence to intervention. The ROBINS-I tool has seven bias domains: confounding, selection of participants into the study, classification of interventions, deviations from intended interventions, missing data, measurement of outcome, and selection of the reported result. To assess confounding for COVID-19 outcomes, we examined whether studies adjusted for population characteristics (age structure, population size) and for social contact or proxies for social contact at baseline (e.g., mobility, population density, occupation, socioeconomic variables such as income or education) [25]. For anxiety and depression outcomes, we assessed whether studies adjusted for age, sex, marital status, and socioeconomic status [26]. For employment outcomes, we assessed adjustment for age, sex, and education [27].

The risk of bias judgment for each ROBINS-I domain is classified as follows: *low* (study is comparable to a well-performed randomized trial), *moderate* (study appears to provide sound evidence for a non-randomized study but cannot be considered comparable to a well-performed randomized trial), *serious* (study has one or more important problems), and *critical* (study is too problematic to provide any useful evidence on the effect of the intervention). It is rare for a non- randomized study to be judged as low risk of confounding because of the potential for residual or unmeasured confounding. Before-after studies are usually judged to have at least serious risk of bias because it is not possible to determine whether pre-post changes are due to the intervention rather than other factors. A particular level of risk of bias for a specific domain will mean that the overall risk of bias across domains for a study is at least this severe for the outcome being assessed.

### Data synthesis

A study could include more than one intervention or more than one outcome. Because studies used several instruments to measure anxiety and depression symptoms, we computed the standardized mean difference (mean difference in each study divided by that study’s standard deviation) to enable comparison across studies [28]. We conducted random-effects meta-analysis to compute pooled effect sizes for anxiety, depression, unemployment, and labor force participation using the Comprehensive Meta-Analysis software [29]. We created funnel plots if there were at least 10 studies and used the Trim and Fill adjustment to estimate the true effect size if there was publication bias [28]. We could not perform meta-analysis of studies on COVID-19 morbidity and mortality because these studies rarely reported sample sizes; we performed the sign test where a non-significant *p*-value (two-sided) supports the null hypothesis that the mean effect across studies is zero [28].

## Results

**Fig. 1:**
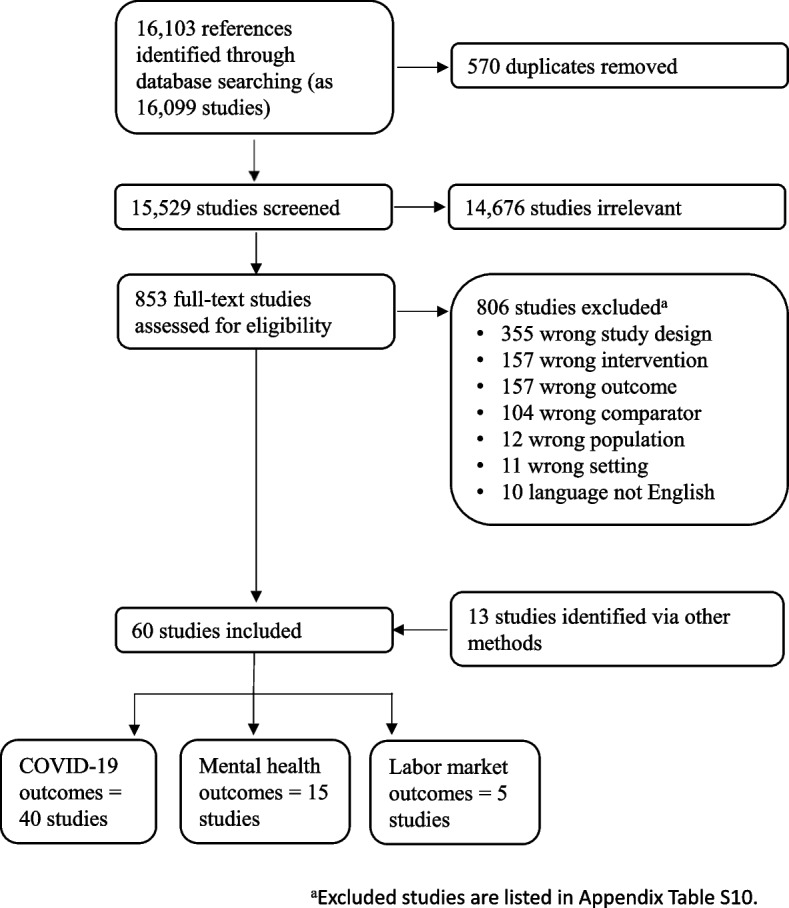
Systematic review of the effects of non-pharmaceutical interventions in non-healthcare workplaces, January 1, 2020–May 11, 2021

Search of the databases yielded 15,529 studies. After screening titles and abstracts, we reviewed the full text of 853 studies for eligibility (Fig. 1). Among these studies, we excluded 806 that did not meet the inclusion criteria. The percent agreement between reviewers was 95% for title and abstract screening and 87% for full-text reviews. We identified 47 observational studies through database searching and 13 via other sources (i.e., examination of references of previous systematic reviews and authors’ knowledge), yielding a total of 60 observational studies that met the inclusion criteria. Forty studies reported on COVID-19 morbidity and mortality outcomes (Appendix Table S4) [30–70], 15 assessed the effect on anxiety and depression symptoms (Appendix Table S5) [71–85], and five assessed the effect on unemployment and labor force participation (Appendix Table S6) [5, 86–89]. The studies were based on data from the first year of the pandemic, mostly covering the period March to July 2020. The domain-specific and overall risk of bias for each study are shown in Appendix Tables S7-S9. Studies that were excluded from the review are listed in Appendix Table S10.

Of the 40 studies on COVID-19 morbidity and mortality, 16 were based on data from the USA, and 13 studies analyzed data from multiple countries, ranging from 2 to 202 countries (Appendix Table S4). Other studies included data from countries in Europe (Spain, Italy, Germany), Asia (India, China), Africa (South Africa), and Australia. The median study period over which outcome data were collected was 10 weeks (interquartile range: 8 weeks, 17 weeks). The overall risk of bias was moderate for 25 studies, serious for 14 studies, and critical for one study (Appendix Table S7). All studies had at least a moderate risk of confounding, and most studies had a low risk of bias for the other six domains. Although studies on physical distancing (teleworking) [41] and physical barriers [45] reported a significant decrease in COVID-19 incidence in workers, and studies on initial business closures (i.e., restaurant or entertainment business closures) reported a significant decrease in COVID-19 case growth rate and epidemic doubling time in the general population [35, 67], the sign tests were not significant (Table 1). Studies on workplace closures reported a decrease in COVID-19 incidence or case growth rate (six of seven studies) and reproduction number (four studies) in the general population, but the sign tests were not significant (Table 1). Studies showed that lockdown significantly decreased COVID-19 incidence or case growth rate (23 studies, *p* < 0.001), reproduction number (11 studies, *p* < 0.001), and COVID-19 mortality or death growth rate (seven studies, *p* < 0.05) in the general population (Table 1). The 23 studies on the effect of lockdown on COVID-19 incidence or case growth rate reported a variety of effect measures, with seven studies reporting percentage decrease in daily case growth rate (median: 6 percentage decrease) [33–35, 42, 50, 56, 65], and six studies reporting the growth rate before and after lockdown (median growth rate before lockdown: 18.0 percentage increase; median growth rate after lockdown: 3.8 percentage increase) [32, 60, 61, 66, 68, 70].

**Table 1:** Studies on the effects of non-pharmaceutical interventions on COVID-19 morbidity and mortality outcomes, January 1, 2020–May 11, 2021

Intervention^a^	Outcome	No. of studies favoring intervention	No. of studies favoring no intervention	Sign test *p*	Overall risk of bias^b^
Working from home or telecommuting [41]	COVID-19 illness	1 (significant)	0	1.0	Serious
Physical barrier [45]	COVID-19 incidence	1 (significant)	0	1.0	Serious
Initial business closure [35]	COVID-19 case growth rate	1 (significant)	0	1.0	Moderate
Initial business closure [67]	Epidemic doubling time	1 (significant)	0	1.0	Moderate
Workplace closure [31, 36, 38, 39, 42, 50, 64]	COVID-19 incidence or case growth rate	6 (5 significant, 1 not significant)	1 (not significant)	0.13	Moderate for 4 studies, serious for 3 studies
Workplace closure [37, 39, 49, 52]	Reproduction number	4 (3 significant, 1 not significant)	0	0.13	Moderate for 1, serious for 2, critical for 1
Workplace closure [67]	Epidemic doubling time	1 (not significant)	0	1.0	Moderate
Workplace closure [50]	COVID-19 death growth rate	1 (not significant)	0	1.0	Moderate
Lockdown [30–36, 42, 46, 50, 51, 53, 55, 56, 58, 60–62, 65, 66, 68–70]	COVID-19 incidence or case growth rate	23 (22 significant, 1 not significant)	0	< 0.001	Moderate for 19, serious for 4
Lockdown [33, 37, 40, 43, 44, 47, 49, 52, 57, 59, 63]	Reproduction number	11 (10 significant, 1 not significant)	0	< 0.001	Moderate for 4, serious for 6, critical for 1
Lockdown [48, 57, 61, 67]	Epidemic doubling time	4 (1 significant, 3 not significant)	0	0.13	Moderate for 2, serious for 2
Lockdown [50, 54, 55, 58, 62, 66, 69]	COVID-19 mortality or death growth rate	7 (7 significant)	0	< 0.05	Moderate for 6, serious for 1

^a^The numbers inside square brackets represent the study references

^b^Overall risk of bias, assessed using the Risk of Bias in Non-Randomized Studies of Interventions (ROBINS-I) tool, is categorized as Low, Moderate, Serious, or Critical

**Fig. 2:**
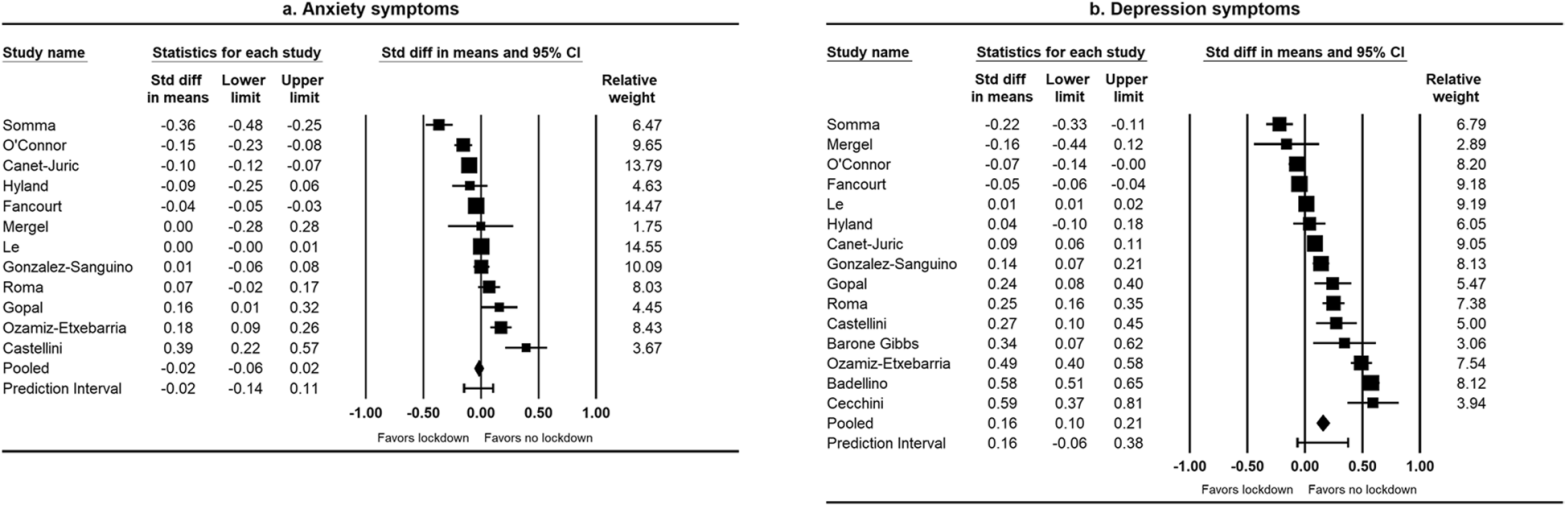
Forest plots of the effect of lockdown on anxiety and depression symptoms, January 1, 2020–May 11, 2021^a^. ^a^I^2^ for heterogeneity for studies on anxiety symptoms = 94% (Q test *p* < 0.001). I^2^ for heterogeneity for studies on depression symptoms = 98% (Q test *p* < 0.001)

**Fig. 3:**
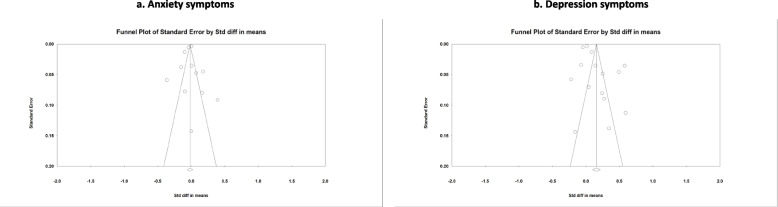
Funnel plots of the effect of lockdown on anxiety and depression symptoms, January 1, 2020–May 11, 2021^a^. ^a^The graph on the left shows studies on anxiety symptoms, and that on the right shows studies on depression symptoms. The Trim and Fill adjusted pooled standardized mean difference for depression symptoms = 0.001 (95% CI: -0.04, 0.02)

Among the 15 studies on anxiety and depression symptoms, 10 were conducted in European countries (Spain, Italy, Germany, Ireland, United Kingdom) and two were conducted in the USA (Appendix Table S5). All studies reported on the effect of lockdown, with the median interval between the baseline and follow-up outcome measurements being 6 weeks. Several instruments were used for assessing anxiety symptoms, including the Generalized Anxiety Disorder Scale [76–79, 82], the Brief Symptom Inventory [74, 81], and the Depression Anxiety and Stress Scale [83, 84]. Instruments for assessing depression symptoms included the Patients Health Questionnaire [71, 76–79, 82], the Brief Symptom Inventory [74, 81], and the Depression Anxiety and Stress Scale [83, 84]. The overall risk of bias was serious for five studies and critical for 10 studies (Appendix Table S8). This was mainly because of risk of bias in the confounding, selection of participants, and missing data domains. Fourteen studies reported the effect of lockdown on anxiety and/or depression symptoms in the general adult population and one study reported the effect on depression symptoms in workers. For the effect of lockdown on anxiety symptoms, the pooled standardized mean difference was -0.02 (95% CI: -0.06, 0.02) (Fig. 2a). For the effect of lockdown on depression symptoms, the pooled standardized mean difference was 0.16 (95% CI: 0.10, 0.21) (Fig. 2b). The funnel plot for depression symptoms showed some asymmetry in the distribution of studies about the pooled standardized mean difference (Fig. 3b), and the Trim and Fill adjustment indicated that publication bias could account for the observed effect (adjusted pooled standardized mean difference = 0.001, 95% CI: -0.04, 0.02).

**Fig. 4:**
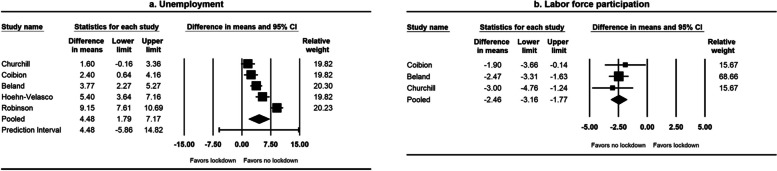
Forest plots of the effect of lockdown on unemployment and labor force participation, January 1, 2020–May 11, 2021^a^. ^a^I^2^ for heterogeneity for studies on unemployment = 92% (Q test *p* < 0.001). I^2^ for heterogeneity for studies on labor force participation = 0% (Q test *p* = 0.69)

Among the five studies on unemployment and labor force participation, three were from the USA, one from Mexico, and one from Australia (Appendix Table S6). The median interval between the baseline and follow-up outcome measurements was 3 months. The overall risk of bias was moderate in two studies and serious in three studies (Appendix Table S9). All studies had a moderate or serious risk of confounding, and one study had a serious risk of bias because of missing data. The studies showed that lockdown increased unemployment (pooled mean difference = 4.48 percentage points, 95% CI: 1.79, 7.17) (Fig. 4a) and decreased labor force participation (pooled mean difference = -2.46 percentage points, 95% CI: -3.16, -1.77) (Fig. 4b).

## Discussion

Empiric studies showed that lockdown reduced COVID-19 incidence or case growth rate, reproduction number, and COVID-19 mortality or death growth rate in the general population during the initial year of COVID-19 pandemic. We found few studies on the effect of NPIs other than lockdown on COVID-19 morbidity and mortality outcomes. Lockdown increased unemployment and decreased labor force participation, but no effect was observed on anxiety symptoms. Lockdown had a small effect on increasing depression symptoms, but publication bias could account for the observed effect. The risk of bias for most of the studies on COVID-19 and employment outcomes was moderate or serious, and that for the studies on anxiety and depression symptoms was serious or critical.

Non-pharmaceutical measures can reduce SARS-CoV-2 transmission by reducing the likelihood of transmission per contact and by reducing contacts between infectious and healthy persons [90]. Studies published in 2023 found that employed adults who had telework experience before illness onset were less likely to work onsite while ill during COVID-19 and other acute respiratory illnesses than persons without telework experience, suggesting that telework may reduce workplace virus exposure [91, 92]. Systematic reviews that assessed the effect of physical distancing and screening in non-workplace settings or on other respiratory viruses provide indirect evidence for the effect of these measures on COVID-19 illness in non-healthcare workplaces. A systematic review assessed the effectiveness of physical distancing measures in non-healthcare workplaces on influenza attack rates [93]. One review included studies of physical distancing on COVID-19 illness in settings other than workplaces (e.g., ≥ 3 vs. ≥ 6 feet distancing policies in schools; frequency of close contact with a primary case in a household) [94]. A Cochrane rapid review assessed the effect of symptom/exposure-based or test-based screening of international travelers for SARS-CoV-2 at borders before or after travel [95]. Systematic reviews of modeling studies on the effect of NPIs within non-healthcare workplaces on COVID-19 illness are needed because modeling studies fill in gaps of information when decisions must be made and there is limited information [96, 97].

Recent systematic reviews of empiric studies have assessed the effect of workplace closures and lockdowns [18, 94, 98]. Two of these reviews included cross-sectional studies [94, 98]. We excluded cross-sectional studies because it is difficult to assess cause-and-effect relationships from such studies [99]. The previous reviews reported that workplace closures and lockdowns reduced COVID-19 incidence, case growth rate, reproduction number, COVID-19 mortality, and death growth rate in the general population [18, 94, 98]. Lockdowns have been shown to reduce population mobility, with increased time at home, reductions in visits to shops and workplaces, and decline in use of public transport [17].

Our systematic review did not find conclusive evidence that lockdown increased anxiety and depression symptoms. A previous rapid review of studies published from January 2020 to June 2020 reported small effects of lockdown on anxiety and depression symptoms [100]. Among the 11 empiric studies on anxiety and depression symptoms included in the review, four were conducted in college or university students and thus not directly relevant to our systematic review. Another review estimated that the global prevalence of anxiety and depression symptoms increased during the COVID-19 pandemic compared to the pre-pandemic period [101]. The authors attributed the increase in anxiety and depression symptoms to the combined effects of the spread of SARS-CoV-2 and the interventions, including lockdown, school and workplace closures, decreased public transport, and reduction of social interactions. Several risk factors for anxiety and depression during lockdown have been reported. Risk factors for anxiety include loneliness and history of mental health issues, while higher level of resilience and spiritual well-being are associated with lower anxiety [77, 78]. Risk factors for depression include loneliness, detachment, negative affect, history of mental health issues, concerns about changes at work and running out of money, and unemployment [71, 77, 84]. On the other hand, protective factors associated with depression include more resilient coping style, higher level of resilience, spiritual well-being, and moderate-to-vigorous physical activity [75, 77, 78, 84].

Our systematic review showed lockdown increased unemployment and decreased labor force participation. Lockdown can directly lead to layoffs because of business closures, cancelation of events, and reduced economic activities. However, in the absence of lockdown, employment can be affected by individuals’ refraining from activities outside their household to reduce their risk of infection, which can lead to decreased consumer spending and business revenues [5, 88]. We did not identify any previous systematic reviews of the effect of lockdown on unemployment and labor force participation.

Findings of our systematic review should be considered in context of at least seven limitations. First, some studies on the effects of workplace closures and lockdowns on COVID-19 outcomes used quasi-experimental designs (controlled before after, interrupted time series) that can allow for causal inferences without randomized trials [102, 103], but it is unclear if the assumptions required to ensure valid causal inference were met. The findings therefore need to be interpreted as showing an association. Second, the included studies often did not describe in detail the interventions that were assessed, which may make it difficult to compare findings across studies. Third, many NPIs were implemented together or within a short time, and so the independent effects of interventions may be difficult to determine [104], particularly for studies that did not have a concurrent control group. Fourth, the number of COVID-19 cases could have been underestimated to a greater degree during the early phase of the pandemic because of limited availability of COVID-19 tests. However, the underestimation would likely bias the effect of an intervention toward the null [105]. Fifth, several studies on the effect of lockdown on anxiety and depression symptoms collected baseline data after the start of lockdown, and so the magnitude of the effect may be under-estimated. In addition, anxiety and depression were assessed using screening questionnaires that identified probable cases, and the findings may not be extrapolated to diagnosed cases of anxiety and depression [101]. Sixth, although our electronic search identified grey literature (e.g., working papers, medRxiv preprints) [36, 40, 51, 86, 88], we did not specifically search preprint databases or dissertations and theses databases. Finally, we limited studies to English when we performed the electronic searches and screened articles, and thus the findings may not be generalizable to studies published in other languages.

However, this systematic review also has several strengths. We assessed both desired effects (i.e., public health benefits) and secondary (unintended / unwanted) effects of NPIs during the initial year of COVID-19 pandemic. Additionally, we used several electronic databases to search for studies and examined the references of previous systematic reviews, which increased the comprehensiveness of the literature search. Next, our review was based on empiric studies that provide direct evidence of effectiveness in real-world settings.

The COVID-19 pandemic had unequal effects on the population, with people who could work remotely faring better in terms of health and socioeconomic wellbeing than persons who were required to work in-person, such as those in goods production or essential industries [1]. Minority and low-income vulnerable persons are over-represented in high-risk essential industries [1, 45, 106]. COVID-19 death rates in the U.S. have been estimated to be substantially higher in Hispanics and non-Hispanic Blacks compared to non-Hispanic Whites [107, 108]. Compared to people working in non-essential sectors, those working in essential sectors (particularly in agriculture, emergency, manufacturing, facilities, and transportation or logistics) were found to have higher COVID-19 deaths [109, 110]. It is important to deploy effective science based NPIs to reduce health inequities and decrease overall disease transmission, especially in industries where work cannot be performed remotely.

## Conclusions

Our systematic review showed that several empiric studies assessed the effect of lockdowns, but there is a paucity of studies on the effects of other interventions undertaken in many workplace settings, including temperature/symptom screening, use of different barrier protections including some which were not previously proposed as an NPI or tested (e.g., plexiglass or curtain partitions), and physical distancing measures within the workplace. With the availability of COVID-19 vaccines and effective therapeutics that reduce hospitalizations and deaths [1], as well as the desire to avoid detrimental effects on daily life and the economy, the use of workplace closures and lockdowns abated after the initial year of the pandemic in most countries. However, because SARS-CoV-2 remains endemic and because it evolved into variants which can evade immunity acquired through prior infection or vaccination and transmit more efficiently [111], use of less disruptive NPIs including better ventilation, face masks, and some variations of physical distancing within the workplace may still have relevance. Addressing the gaps in the evidence base on the effects of NPIs pertaining to workplaces is therefore important for informing ongoing prevention strategies as well as future pandemic preparedness.

There was scarce direct evidence on the benefits of symptom and/or temperature screening, physical barriers, and physical distancing measures to reduce COVID-19 illness within workplaces that are open. While the use of these interventions is less likely to be perceived as disruptive for work process than lockdowns, they are not likely to be effective in reducing the transmission of an airborne virus like SARS-CoV-2 that can be readily spread in indoor settings by asymptomatic or pre-symptomatic individuals. There was evidence to indicate that lockdown helped reduce COVID-19 morbidity and mortality in the general population, but it increased unemployment and reduced labor force participation. It is important for countries that implement lockdown in future outbreaks of emerging infectious diseases or pandemics to consider strategies to mitigate these unintended consequences.

### Supplementary Information

**Additional file 1: Table S1.** Guidance for the implementation of non-pharmaceutical interventions to prevent the transmission of COVID-19 in non-healthcare workplaces, 2020. **Table S2.** PRISMA 2020 checklist. **Table S3.** Search strategy, January 1, 2020–May 11, 2021. **Table S4.** Characteristics and results of studies assessing effect of physical distancing, physical barriers, workplace closures, and lockdowns, January 1, 2020-May 11, 2021: COVID-19 morbidity and mortality outcomes. **Table S5.** Characteristics and results of studies assessing effect of lockdowns, January 1, 2020-May 11, 2021: Anxiety and depression symptoms. **Table S6.** Characteristics and results of studies assessing effect of lockdowns, January 1, 2020-May 11, 2021: Unemployment and labor force participation. **Table S7.** Risk of bias assessment for studies, January 1, 2020–May 11, 2021: COVID-19 morbidity and mortality outcomes. **Table S8.** Risk of bias assessment for studies, January 1, 2020–May 11, 2021: Anxiety and depression symptoms. **Table S9.** Risk of bias assessment for studies, January 1, 2020–May 11, 2021: Unemployment and labor force participation outcomes. **Table S10.** Studies excluded from the review and reasons for exclusion, January 1, 2020–May 11, 2021.

## Data Availability

All data generated or analyzed during this study are included in this published study and its Additional file 1.

## Abbreviations

CINAHL: Cumulative Index to Nursing and Allied Health Literature
NPI: Non-pharmaceutical intervention
PRISMA: Preferred Reporting Items for Systematic Reviews and Meta-Analysis
ROBINS-I: Risk of Bias in Non-Randomized Studies of Interventions
SARS-CoV-2: Severe Acute Respiratory Syndrome Coronavirus 2
SD: Standard deviation
SE: Standard error
