# Supplementary material for: Systematic review of empiric studies on lockdowns, workplace closures, and other non-pharmaceutical interventions in non-healthcare workplaces during the initial year of the COVID-19 pandemic: benefits and selected unintended consequences
Source: BMC Public Health. 2024 Mar 22;24:884. doi: 10.1186/s12889-024-18377-1 (PMC10960383; doi:10.1186/s12889-024-18377-1)
Supplement: Supplementary file 1 — Additional file 1: Table S1. Guidance for the implementation of non-pharmaceutical interventions to prevent the transmission of COVID-19 in non-healthcare workplaces, 2020. Table S2. PRISMA 2020 checklist. Table S3. Search strategy, January 1, 2020–May 11, 2021. Table S4. Characteristics and results of studies assessing effect of physical distancing, physical barriers, workplace closures, and lockdowns, January 1, 2020-May 11, 2021: COVID-19 morbidity and mortality outcomes. Table S5. Characteristics and results of studies assessing effect of lockdowns, January 1, 2020-May 11, 2021: Anxiety and depression symptoms. Table S6. Characteristics and results of studies assessing effect of lockdowns, January 1, 2020-May 11, 2021: Unemployment and labor force participation. Table S7. Risk of bias assessment for studies, January 1, 2020–May 11, 2021: COVID-19 morbidity and mortality outcomes. Table S8. Risk of bias assessment for studies, January 1, 2020–May 11, 2021: Anxiety and depression symptoms. Table S9. Risk of bias assessment for studies, January 1, 2020–May 11, 2021: Unemployment and labor force participation outcomes. Table S10. Studies excluded from the review and reasons for exclusion, January 1, 2020–May 11, 2021. [file 12889_2024_18377_MOESM1_ESM.docx]

**Additional file 1: Appendix**

**Systematic review of empiric studies on lockdowns, workplace closures, and other non-pharmaceutical interventions in non-healthcare workplaces during the initial year of the COVID-19 pandemic: benefits and selected unintended consequences**

Table of Contents

[Table S1. Guidance for the implementation of non-pharmaceutical interventions to prevent the transmission of COVID-19 in non-healthcare workplaces, 2020 2](#_Toc161925377)

[Table S2. PRISMA 2020 checklist 5](#_Toc161925378)

[Table S3. Search strategy, January 1, 2020–May 11, 2021 9](#_Toc161925379)

[Table S4. Characteristics and results of studies assessing effect of physical distancing, physical barriers, workplace closures, and lockdowns, January 1, 2020-May 11, 2021: COVID-19 morbidity and mortality outcomes 13](#_Toc161925380)

[Table S5. Characteristics and results of studies assessing effect of lockdowns, January 1, 2020-May 11, 2021: Anxiety and depression symptoms 31](#_Toc161925381)

[Table S6. Characteristics and results of studies assessing effect of lockdowns, January 1, 2020-May 11, 2021: Unemployment and labor force participation 36](#_Toc161925382)

[Table S7. Risk of bias assessment for studies, January 1, 2020–May 11, 2021: COVID-19 morbidity and mortality outcomes 38](#_Toc161925383)

[Table S8. Risk of bias assessment for studies, January 1, 2020–May 11, 2021: Anxiety and depression symptoms 40](#_Toc161925384)

[Table S9. Risk of bias assessment for studies, January 1, 2020–May 11, 2021: Unemployment and labor force participation 41](#_Toc161925385)

[Table S10. Studies excluded from the review and reasons for exclusion, January 1, 2020–May 11, 2021 42](#_Toc161925386)

[References 164](#_Toc161925387)

## Table S1. Guidance for the implementation of non-pharmaceutical interventions to prevent the transmission of COVID-19 in non-healthcare workplaces, 2020

| **Measure** | **WHO (May 2020)^a^** | **ECDC (September 2020)^b^** | **US CDC (December 2020)^c^** |
| --- | --- | --- | --- |
| Hand hygiene | Handwashing with soap and water; use alcohol-based hand-sanitizer | Handwashing with soap and water; use hand sanitizer | Handwashing with soap and water; use alcohol-based hand sanitizer |
| Respiratory hygiene | Respiratory etiquette; use face mask or covering | Respiratory etiquette; use face mask | Respiratory etiquette; use face mask or cloth face covering |
| Physical distancing | Implement or enhance shift or split-team arrangements or teleworking; avoid crowding by staggering working hours; distance of at least 1 meter between people; avoid direct physical contact (e.g., handshaking); minimize need for physical meetings (e.g., by using teleconferencing); defer or suspend workplace events | Opportunity for distance working/ teleworking; flexible working schedules/ shifts for employees; physical distancing measures within the workspace; increase use of email and teleconferences to reduce close contacts; reduce contact between employees; reduce contact between employees and customers | Flexible worksites (e.g., telework); flexible work hours (e.g., rotate or stagger shifts); increase physical space between employees; prohibit handshaking; flexible meeting and travel options (e.g., videoconferencing or teleconferencing); close or limit access to common areas; cancel group events; deliver services remotely (e.g., phone, video, web); reduce close contact with customers (e.g., drive-through service, online shopping, curbside pickup, delivery options) |
| Physical barrier | Install plexiglass barriers at all points of regular interaction for workplaces with close, frequent contact with the general public, or other co-workers, visitors, clients or customers, or contractors | – | Configure partitions as a barrier |
| Environmental cleaning and disinfection | Priority disinfection of high-touch areas | – | Clean and disinfect frequently touched objects and surfaces |
| Symptom and temperature screening | Thermal screening at workplace | – | Symptom and temperature screening before entering facility^d^ |
| Isolation | Self-isolation at home if sick | Self-isolation at home if respiratory symptoms; flexible leave policy | Self-isolation at home if symptoms; flexible non-punitive paid sick leave |
| Quarantine | Quarantine for 14 days if close contact with a COVID-19 case at the workplace | – | Quarantine for 14 days if close contact with a COVID-19 case |
| Work-related travel | Cancel or postpone non-essential travel | – | Delay travel |
| Building engineering control | Natural or mechanical ventilation, preferably without re-circulation of the air, for workplaces with close, frequent contact with the general public, or other co-workers, visitors, clients or customers, or contractors | Natural or mechanical ventilation; avoid air recirculation without filtration | Natural or mechanical ventilation; air filtration; ultraviolet germicidal irradiation if options for increasing ventilation are limited |

^a^World Health Organization. Considerations for public health and social measures in the workplace in the context of COVID-19. https://www.who.int/publications/i/item/considerations-for-public-health-and-social-measures-in-the-workplace-in-the-context-of-covid-19

^b^European Centre for Disease Prevention and Control. Guidelines for the implementation of non-pharmaceutical interventions against COVID-19. https://www.ecdc.europa.eu/sites/default/files/documents/covid-19-guidelines-non-pharmaceutical-interventions-september-2020.pdf

^c^Centers for Disease Control and Prevention. Guidance for Businesses and Employers Responding to Coronavirus Disease 2019 (COVID-19). <https://public4.pagefreezer.com/browse/CDC%20Covid%20Pages/11-05-2022T12:30/https://www.cdc.gov/coronavirus/2019-ncov/community/guidance-business-response.html>

^d^US Centers for Disease Control and Prevention issued an updated guidance on March 8, 2021 to include screening testing for SARS-CoV-2. <https://public4.pagefreezer.com/browse/CDC%20Covid%20Pages/30-07-2021T14:07/https://www.cdc.gov/coronavirus/2019-ncov/community/guidance-business-response.html#previous>

## Table S2. PRISMA 2020 checklist

PRISMA 2020 checklist

| **Section and Topic** | **Item #** | **Checklist item** | **Location where item is reported** |
| --- | --- | --- | --- |
| **TITLE** | | |  |
| Title | 1 | Identify the report as a systematic review. | Page 1 |
| **ABSTRACT** | | |  |
| Abstract | 2 | See the PRISMA 2020 for Abstracts checklist. | Pages 1-2 |
| **INTRODUCTION** | | |  |
| Rationale | 3 | Describe the rationale for the review in the context of existing knowledge. | Page 2 |
| Objectives | 4 | Provide an explicit statement of the objective(s) or question(s) the review addresses. | Page 2 |
| **METHODS** | | |  |
| Eligibility criteria | 5 | Specify the inclusion and exclusion criteria for the review and how studies were grouped for the syntheses. | Pages 3-4 |
| Information sources | 6 | Specify all databases, registers, websites, organisations, reference lists and other sources searched or consulted to identify studies. Specify the date when each source was last searched or consulted. | Page 3 |
| Search strategy | 7 | Present the full search strategies for all databases, registers and websites, including any filters and limits used. | Table S3 |
| Selection process | 8 | Specify the methods used to decide whether a study met the inclusion criteria of the review, including how many reviewers screened each record and each report retrieved, whether they worked independently, and if applicable, details of automation tools used in the process. | Page 4 |
| Data collection process | 9 | Specify the methods used to collect data from reports, including how many reviewers collected data from each report, whether they worked independently, any processes for obtaining or confirming data from study investigators, and if applicable, details of automation tools used in the process. | Page 4 |
| Data items | 10a | List and define all outcomes for which data were sought. Specify whether all results that were compatible with each outcome domain in each study were sought (e.g. for all measures, time points, analyses), and if not, the methods used to decide which results to collect. | Page 3 |
|  | 10b | List and define all other variables for which data were sought (e.g. participant and intervention characteristics, funding sources). Describe any assumptions made about any missing or unclear information. | Page 3 |
| Study risk of bias assessment | 11 | Specify the methods used to assess risk of bias in the included studies, including details of the tool(s) used, how many reviewers assessed each study and whether they worked independently, and if applicable, details of automation tools used in the process. | Page 4 |
| Effect measures | 12 | Specify for each outcome the effect measure(s) (e.g. risk ratio, mean difference) used in the synthesis or presentation of results. | Page 4 |
| Synthesis methods | 13a | Describe the processes used to decide which studies were eligible for each synthesis (e.g. tabulating the study intervention characteristics and comparing against the planned groups for each synthesis (item #5)). | Page 4 |
|  | 13b | Describe any methods required to prepare the data for presentation or synthesis, such as handling of missing summary statistics, or data conversions. | Page 4 |
|  | 13c | Describe any methods used to tabulate or visually display results of individual studies and syntheses. | Page 4 |
|  | 13d | Describe any methods used to synthesize results and provide a rationale for the choice(s). If meta-analysis was performed, describe the model(s), method(s) to identify the presence and extent of statistical heterogeneity, and software package(s) used. | Page 4 |
|  | 13e | Describe any methods used to explore possible causes of heterogeneity among study results (e.g. subgroup analysis, meta-regression). | Page 4 |
|  | 13f | Describe any sensitivity analyses conducted to assess robustness of the synthesized results. | Page 4 |
| Reporting bias assessment | 14 | Describe any methods used to assess risk of bias due to missing results in a synthesis (arising from reporting biases). | Page 4 |
| Certainty assessment | 15 | Describe any methods used to assess certainty (or confidence) in the body of evidence for an outcome. | Page 4 |
| **RESULTS** | | |  |
| Study selection | 16a | Describe the results of the search and selection process, from the number of records identified in the search to the number of studies included in the review, ideally using a flow diagram. | Fig. 1 |
|  | 16b | Cite studies that might appear to meet the inclusion criteria, but which were excluded, and explain why they were excluded. | Table S10 |
| Study characteristics | 17 | Cite each included study and present its characteristics. | Tables S4-S6 |
| Risk of bias in studies | 18 | Present assessments of risk of bias for each included study. | Tables S7-S9 |
| Results of individual studies | 19 | For all outcomes, present, for each study: (a) summary statistics for each group (where appropriate) and (b) an effect estimate and its precision (e.g. confidence/credible interval), ideally using structured tables or plots. | Table 1, Figs. 2-4 |
| Results of syntheses | 20a | For each synthesis, briefly summarise the characteristics and risk of bias among contributing studies. | Pages 5-7 |
|  | 20b | Present results of all statistical syntheses conducted. If meta-analysis was done, present for each the summary estimate and its precision (e.g. confidence/credible interval) and measures of statistical heterogeneity. If comparing groups, describe the direction of the effect. | Pages 5-7, Figs. 2, 4 |
|  | 20c | Present results of all investigations of possible causes of heterogeneity among study results. | Not applicable |
|  | 20d | Present results of all sensitivity analyses conducted to assess the robustness of the synthesized results. | Page 6 |
| Reporting biases | 21 | Present assessments of risk of bias due to missing results (arising from reporting biases) for each synthesis assessed. | Pages 5-6 |
| Certainty of evidence | 22 | Present assessments of certainty (or confidence) in the body of evidence for each outcome assessed. | Pages 5-6 |
| **DISCUSSION** | | |  |
| Discussion | 23a | Provide a general interpretation of the results in the context of other evidence. | Page 8 |
|  | 23b | Discuss any limitations of the evidence included in the review. | Pages 8-9 |
|  | 23c | Discuss any limitations of the review processes used. | Pages 8-9 |
|  | 23d | Discuss implications of the results for practice, policy, and future research. | Page 9 |
| **OTHER INFORMATION** | | |  |
| Registration and protocol | 24a | Provide registration information for the review, including register name and registration number, or state that the review was not registered. | Page 2 |
|  | 24b | Indicate where the review protocol can be accessed, or state that a protocol was not prepared. | Page 2 |
|  | 24c | Describe and explain any amendments to information provided at registration or in the protocol. | Page 2 |
| Support | 25 | Describe sources of financial or non-financial support for the review, and the role of the funders or sponsors in the review. | Page 10 |
| Competing interests | 26 | Declare any competing interests of review authors. | Page 10 |
| Availability of data, code and other materials | 27 | Report which of the following are publicly available and where they can be found: template data collection forms; data extracted from included studies; data used for all analyses; analytic code; any other materials used in the review. | Page 10 |

PRISMA 2020 for Abstracts checklist

| **Section and Topic** | **Item #** | **Checklist item** | **Reported (Yes/No)** |
| --- | --- | --- | --- |
| **TITLE** | | |  |
| Title | 1 | Identify the report as a systematic review. | Yes |
| **BACKGROUND** | | |  |
| Objectives | 2 | Provide an explicit statement of the main objective(s) or question(s) the review addresses. | Yes |
| **METHODS** | | |  |
| Eligibility criteria | 3 | Specify the inclusion and exclusion criteria for the review. | Yes |
| Information sources | 4 | Specify the information sources (e.g. databases, registers) used to identify studies and the date when each was last searched. | Yes |
| Risk of bias | 5 | Specify the methods used to assess risk of bias in the included studies. | Yes |
| Synthesis of results | 6 | Specify the methods used to present and synthesise results. | Yes |
| **RESULTS** | | |  |
| Included studies | 7 | Give the total number of included studies and participants and summarise relevant characteristics of studies. | Yes |
| Synthesis of results | 8 | Present results for main outcomes, preferably indicating the number of included studies and participants for each. If meta-analysis was done, report the summary estimate and confidence/credible interval. If comparing groups, indicate the direction of the effect (i.e. which group is favoured). | Yes |
| **DISCUSSION** | | |  |
| Limitations of evidence | 9 | Provide a brief summary of the limitations of the evidence included in the review (e.g. study risk of bias, inconsistency and imprecision). | Yes |
| Interpretation | 10 | Provide a general interpretation of the results and important implications. | Yes |
| **OTHER** | | |  |
| Funding | 11 | Specify the primary source of funding for the review. | Not applicable (no funding) |
| Registration | 12 | Provide the register name and registration number. | Yes |

## Table S3. Search strategy, January 1, 2020–May 11, 2021

| **Database** | **Strategy** |
| --- | --- |
| **Medline**  **(OVID)**  **1946-** | Novel coronavirus OR novel corona virus OR new coronavirus OR new corona virus OR covid19 OR covid 19 OR nCoV OR novel CoV OR CoV 2 OR CoV2 OR sarscov2 OR 2019nCoV OR 2019-nCoV  AND  distancing OR distance OR social network* OR isolation OR social contact* OR worker contact* OR limit contact OR spacing OR community mitigation OR non-pharmaceutical OR nonpharmaceutical OR (work* ADJ2 home*) OR (stay* ADJ2 home*) OR sick leave OR paid leave OR teleconferenc* OR tele-work* OR telework* OR (cancel ADJ2 gathering*) OR (restrict* ADJ2 gathering*) OR (postpone ADJ2 gathering*) OR (avoid ADJ2 gathering*) OR (cancel ADJ2 meeting*) OR (postpone ADJ2 meeting*) OR (avoid ADJ2 meeting*) OR (restrict* ADJ2 meeting*) OR (avoid ADJ2 congregating) OR social behavior* OR social behaviour* OR social interaction* OR face-to-face OR (restrict* ADJ2 interaction*) OR (restrict* ADJ2 contact*) OR (shar* ADJ2 equipment) OR social mixing OR fever check* OR health check* OR screening OR lockdown* OR exit strateg* OR mobilit* OR control measure* OR control strateg* OR partition* OR (modified ADJ3 space*)  **AND**  Workplace* OR work place* OR work site* OR worksite* OR jobsite* OR job site* OR worker* OR employee* OR occupational health OR office* OR "on the job" OR "at work" OR industry OR industries OR factory OR factories OR processing plant* OR processing facilit* OR restaurant* OR grocery store* OR lockdown*  NOT  Exp animals/ NOT exp humans/  Limit English |
| **Embase**  **(OVID)**  **1996-** | Novel coronavirus OR novel corona virus OR new coronavirus OR new corona virus OR covid19 OR covid 19 OR nCoV OR novel CoV OR CoV 2 OR CoV2 OR sarscov2 OR 2019nCoV OR 2019-nCoV  AND  (  distancing OR distance OR social network* OR isolation OR social contact* OR worker contact* OR limit contact OR spacing OR community mitigation OR non-pharmaceutical OR nonpharmaceutical OR (work* ADJ2 home*) OR (stay* ADJ2 home*) OR sick leave OR paid leave OR teleconferenc* OR tele-work* OR telework* OR (cancel ADJ2 gathering*) OR (restrict* ADJ2 gathering*) OR (postpone ADJ2 gathering*) OR (avoid ADJ2 gathering*) OR (cancel ADJ2 meeting*) OR (postpone ADJ2 meeting*) OR (avoid ADJ2 meeting*) OR (restrict* ADJ2 meeting*) OR (avoid ADJ2 congregating) OR social behavior* OR social behaviour* OR social interaction* OR face-to-face OR (restrict* ADJ2 interaction*) OR (restrict* ADJ2 contact*) OR (shar* ADJ2 equipment) OR social mixing OR fever check* OR health check* OR screening OR lockdown* OR exit strateg* OR mobilit* OR control measure* OR control strateg* OR partition* OR (modified ADJ3 space*)  **AND**  Workplace* OR work place* OR work site* OR worksite* OR jobsite* OR job site* OR worker* OR employee* OR occupational health OR office* OR "on the job" OR "at work" OR industry OR industries OR factory OR factories OR processing plant* OR processing facilit* OR restaurant* OR grocery store* OR lockdown*  )  NOT  Exp animal/ NOT exp human/  Limit English; not pubmed/medline |
| **PsycInfo**  **(OVID)**  **1987-** | Novel coronavirus OR novel corona virus OR new coronavirus OR new corona virus OR covid19 OR covid 19 OR nCoV OR novel CoV OR CoV 2 OR CoV2 OR sarscov2 OR 2019nCoV OR 2019-nCoV  AND  (  distancing OR distance OR social network* OR isolation OR social contact* OR worker contact* OR limit contact OR spacing OR community mitigation OR non-pharmaceutical OR nonpharmaceutical OR (work* ADJ2 home*) OR (stay* ADJ2 home*) OR sick leave OR paid leave OR teleconferenc* OR tele-work* OR telework* OR (cancel ADJ2 gathering*) OR (restrict* ADJ2 gathering*) OR (postpone ADJ2 gathering*) OR (avoid ADJ2 gathering*) OR (cancel ADJ2 meeting*) OR (postpone ADJ2 meeting*) OR (avoid ADJ2 meeting*) OR (restrict* ADJ2 meeting*) OR (avoid ADJ2 congregating) OR social behavior* OR social behaviour* OR social interaction* OR face-to-face OR (restrict* ADJ2 interaction*) OR (restrict* ADJ2 contact*) OR (shar* ADJ2 equipment) OR social mixing OR fever check* OR health check* OR screening OR lockdown* OR exit strateg* OR mobilit* OR control measure* OR control strateg* OR partition* OR (modified ADJ3 space*)  **AND**  Workplace* OR work place* OR work site* OR worksite* OR jobsite* OR job site* OR worker* OR employee* OR occupational health OR office* OR "on the job" OR "at work" OR industry OR industries OR factory OR factories OR processing plant* OR processing facilit* OR restaurant* OR grocery store* OR lockdown*  )  Limit English |
| **CINAHL**  **(Ebsco)**  **1982-** | “Novel coronavirus” OR “novel corona virus” OR “new coronavirus” OR “new corona virus” OR covid19 OR “covid 19” OR nCoV OR “novel CoV” OR “CoV 2” OR CoV2 OR sarscov2 OR 2019nCoV OR 2019-nCoV  AND  (  distancing OR distance OR "social network*" OR isolation OR "social contact*" OR "worker contact*" OR "limit contact" OR spacing OR "community mitigation" OR non-pharmaceutical OR nonpharmaceutical OR (work N2 home) OR (stay* N2 home) OR "sick leave" OR “paid leave” OR teleconferenc* OR tele-work OR telework OR (cancel N2 gathering*) OR (restrict* N2 gathering*) OR (postpone N2 gathering*) OR (avoid N2 gathering*) OR (cancel N2 meeting*) OR (postpone N2 meeting*) OR (avoid N2 meeting*) OR (restrict* N2 meeting*) OR (avoid N2 congregating) OR "social behavior*" OR "social behaviour*" OR "social interaction*" OR face-to-face OR (restrict* N2 interaction*) OR (restrict* N2 contact*) OR (shar* N2 equipment) OR "social mixing" OR “fever check*” OR “health check*” OR screening OR lockdown* OR “exit strateg*” OR mobilit* OR “control measure*” OR “control strateg*” OR partition* OR (modified N3 space*)  **AND**  Workplace* OR "work place*" OR "work site*" OR worksite* OR jobsite* OR "job site*" OR worker* OR employee* OR "occupational health" OR office* OR "on the job" OR "at work" OR industry OR industries OR factory OR factories OR “processing plant*” OR “processing facilit*” OR restaurant* OR “grocery store*” OR lockdown*  )  Limit English; Exclude Medline Records ; Human |
| **Econlit**  **(Ebsco)**  **1886-** | “Novel coronavirus” OR “novel corona virus” OR “new coronavirus” OR “new corona virus” OR covid19 OR “covid 19” OR nCoV OR “novel CoV” OR “CoV 2” OR CoV2 OR sarscov2 OR 2019nCoV OR 2019-nCoV  AND  (  distancing OR distance OR "social network*" OR isolation OR "social contact*" OR "worker contact*" OR "limit contact" OR spacing OR "community mitigation" OR non-pharmaceutical OR nonpharmaceutical OR (work N2 home) OR (stay* N2 home) OR "sick leave" OR “paid leave” OR teleconferenc* OR tele-work OR telework OR (cancel N2 gathering*) OR (restrict* N2 gathering*) OR (postpone N2 gathering*) OR (avoid N2 gathering*) OR (cancel N2 meeting*) OR (postpone N2 meeting*) OR (avoid N2 meeting*) OR (restrict* N2 meeting*) OR (avoid N2 congregating) OR "social behavior*" OR "social behaviour*" OR "social interaction*" OR face-to-face OR (restrict* N2 interaction*) OR (restrict* N2 contact*) OR (shar* N2 equipment) OR "social mixing" OR “fever check*” OR “health check*” OR screening OR lockdown* OR “exit strateg*” OR mobilit* OR “control measure*” OR “control strateg*” OR partition* OR (modified N3 space*)  **AND**  Workplace* OR "work place*" OR "work site*" OR worksite* OR jobsite* OR "job site*" OR worker* OR employee* OR "occupational health" OR office* OR "on the job" OR "at work" OR industry OR industries OR factory OR factories OR “processing plant*” OR “processing facilit*” OR restaurant* OR “grocery store*” OR lockdown*  )  Limit English |
| **Cochrane** | “Novel coronavirus” OR “novel corona virus” OR “new coronavirus” OR “new corona virus” OR covid19 OR “covid 19” OR nCoV OR “novel CoV” OR “CoV 2” OR CoV2 OR sarscov2 OR 2019nCoV OR 2019-nCoV  AND  (  distancing OR distance OR "social network*" OR isolation OR "social contact*" OR "worker contact*" OR "limit contact" OR spacing OR "community mitigation" OR non-pharmaceutical OR nonpharmaceutical OR (work NEAR/2 home) OR (stay* NEAR/2 home) OR "sick leave" OR “paid leave” OR teleconferenc* OR tele-work OR telework OR (cancel NEAR/2 gathering*) OR (restrict* NEAR/2 gathering*) OR (postpone NEAR/2 gathering*) OR (avoid NEAR/2 gathering*) OR (cancel NEAR/2 meeting*) OR (postpone NEAR/2 meeting*) OR (avoid NEAR/2 meeting*) OR (restrict* NEAR/2 meeting*) OR (avoid NEAR/2 congregating) OR "social behavior*" OR "social behaviour*" OR "social interaction*" OR face-to-face OR (restrict* NEAR/2 interaction*) OR (restrict* NEAR/2 contact*) OR (shar* NEAR/2 equipment) OR "social mixing" OR “fever check*” OR “health check*” OR screening OR lockdown* OR “exit strateg*” OR mobilit* OR “control measure*” OR “control strateg*” OR partition* OR (modified NEAR/3 space*)  AND  Workplace* OR "work place*" OR "work site*" OR worksite* OR jobsite* OR "job site*" OR worker* OR employee* OR "occupational health" OR office* OR "on the job" OR "at work" OR industry OR industries OR factory OR factories OR “processing plant*” OR “processing facilit*” OR restaurant* OR “grocery store*” OR lockdown*  )  Limit English |
| **Scopus** | TITLE-ABS-KEY(“Novel coronavirus” OR “novel corona virus” OR “new coronavirus” OR “new corona virus” OR covid19 OR “covid 19” OR nCoV OR “novel CoV” OR “CoV 2” OR CoV2 OR sarscov2 OR 2019nCoV OR 2019-nCoV) AND TITLE-ABS-KEY(distancing OR distance OR "social network*" OR isolation OR "social contact*" OR "worker contact*" OR "limit contact" OR spacing OR "community mitigation" OR non-pharmaceutical OR nonpharmaceutical OR (work W/2 home) OR (stay* W/2 home) OR "sick leave" OR teleconferenc* OR tele-work OR telework OR (cancel W/2 gathering*) OR (restrict* W/2 gathering*) OR (postpone W/2 gathering*) OR (avoid W/2 gathering*) OR (cancel W/2 meeting*) OR (postpone W/2 meeting*) OR (avoid W/2 meeting*) OR (restrict* W/2 meeting*) OR (avoid W/2 congregating) OR "social behavior*" OR "social behaviour*" OR "social interaction*" OR face-to-face OR (restrict* W/2 interaction*) OR (restrict* W/2 contact*) OR (shar* W/2 equipment) OR "social mixing" OR “fever check*” OR “health check*” OR screening OR lockdown* OR “exit strateg*” OR mobilit* OR “control measure*” OR “control strateg*” OR partition* OR (modified W/3 space*)) **AND** TITLE-ABS-KEY(Workplace* OR "work place*" OR "work site*" OR "worksite*" OR worker* OR employee* OR "occupational health" OR office* OR "on the job" OR "at work" OR industry OR industries OR factory OR factories OR “processing plant*” OR “processing facilit*” OR restaurant* OR “grocery store*” OR lockdown*) AND NOT INDEX(medline) AND NOT INDEX(embase)  Limit English |
| **NIOSHTIC-2** | Novel coronavirus OR novel corona virus OR new coronavirus OR new corona virus OR covid19 OR covid 19 OR nCoV OR novel CoV OR CoV 2 OR CoV2 OR sarscov2 OR 2019nCoV OR 2019 nCoV  AND  social distancing OR community mitigation OR nonpharmaceutical OR non-pharmaceutical OR isolation OR telework OR sick leave OR social mixing |

## Table S4. Characteristics and results of studies assessing effect of physical distancing, physical barriers, workplace closures, and lockdowns, January 1, 2020-May 11, 2021: COVID-19 morbidity and mortality outcomes

| **First author, year published** | **Country** | **Population in whom outcomes assessed**  **(sample size)** | **Intervention (comparator)** | **COVID-19 morbidity and mortality outcomes** | | | | **Overall risk of bias (study design)^a^** | **Funding source** |
| --- | --- | --- | --- | --- | --- | --- | --- | --- | --- |
|  |  |  |  | **COVID-19 incidence or case growth rate** | **Epidemic doubling time** | **Reproduction number** | **COVID-19 mortality or death growth rate** |  |  |
| Alfano 2020  [30] | 202 countries | General population (COVID-19 daily cases, January 22-May 10, 2020. 109 daily observations in 202 countries, giving 22,018 observations) | Countries with lockdown (vs. countries with no lockdown) | Lockdown reduced new COVID-19 cases (after 10 days, beta = -73.3, t = -3.99, p<0.01; after 20 days, beta = -220.0, t = -10.27, p<0.01) | Not reported | Not reported | Not reported | Serious (cohort) | None |
| Askitas 2021  [31] | 175 countries | General population (COVID-19 daily cases in 175 countries that acted before the first 300 cases, January 3-February 6, 2020) | Lockdown (vs. no lockdown)  Workplace closure (vs. no closure) | Lockdown significantly reduced daily COVID-19 incidence over a limited number of days  Workplace closure led to a 15 percentage decrease in daily COVID-19 incidence after 6 weeks, p<0.05 | Not reported | Not reported | Not reported | Moderate (controlled before after) | None |
| Castillo 2020  [32] | USA | General population (COVID-19 daily cases in 42 states and Washington DC) | 42 states and Washington DC issued lockdown between March 19 and April 7, 2020 | The average rate of increase was 0.113 (95% CI = 0.110, 0.115) pre-lockdown and 0.047 (95% CI = 0.045, 0.048) post-lockdown. Standardized mean difference in case rates from before to after lockdown = -6.847 (95% CI = -6.831,  -6.863, p<0.0001). | Not reported | Not reported | Not reported | Serious (before after) | Not stated |
| Chae 2020  [33] | Germany | General population (COVID-19 daily cases, March 15-May 11, 2020) | 19 counties introduced fines for lockdown violations on March 20 (vs. 19 counties that did not have fines) | Daily growth rate decreased by 6 percentage points (95% CI = -11, -2, p=0.005) | Not reported | Decreased by 0.32 (95% CI = -0.46, -0.18, p<0.001) | Not reported | Moderate (controlled before after) | Not stated |
| Cobb 2020  [34] | USA | General population (COVID-19 daily cases in 246 counties, January 21-March 31, 2020) | 186 counties with lockdown on or after March 19 (vs. 60 counties with no lockdown) | Growth rate decreased by 7.8% (p<0.01) | Not reported | Not reported | Not reported | Moderate (controlled before after) | None |
| Courtemanche 2020 [35] | USA | General population (COVID-19 daily cases in 3,138 counties, March 1-April 27, 2020) | Lockdown (vs. no lockdown)  Restaurant or entertainment business closure (vs. no closure) | Lockdown reduced COVID-19 daily growth rate by 5.9 percentage points (95% CI = -8.9, -2.9) after 16-20 days  Restaurant or entertainment business closure reduced daily growth rate by 5.6 percentage points (95% CI = -10.6, -0.6) after 16-20 days | Not reported | Not reported | Not reported | Moderate (controlled before after) | Not stated |
| Deb 2020  [36] | 129 countries | General population (COVID-19 daily cases, January 22-June 15, 2020) | Lockdown (vs. no lockdown).  Workplace closure (vs. no closures) | Lockdown significantly reduced COVID-19 cases  Workplace closure reduced COVID-19 cases (not significant) | Not reported | Not reported | Not reported | Moderate (controlled before after) | Not stated |
| Dreher 2021  [37] | USA | General population (COVID-19 daily cases in 48 states and Washington DC, up to April 30, 2020) | 15 states with lockdown prior to the 500th case (vs. 34 states without lockdown at 500^th^ case)  States with workplace closure prior to the 500^th^ case (vs. states without closure at 500^th^ case) | Not reported | Not reported | Lockdown reduced reproduction number the week following 500 cases (beta = -0.15, 95% CI = -0.23, -0.07)  Workplace closure reduced reproduction number the week following 500 cases (beta = -0.13, 95% CI = -0.21, -0.05) | Not reported | Moderate (cohort) | Not stated |
| Duhon 2021  [38] | Countries with at least a 6-day period of at least 30 daily cases as of April 23, 2020 | General population (COVID-19 daily cases up to July 29, 2020) | Workplace closure (vs. no closure) | Non-significant increase in daily growth rate (Beta = 0.0354, std error = 0.0239, t=1.479, p=0.1474) | Not reported | Not reported | Not reported | Serious (before after) | Canada's International Development Research Centre, Canadian Institute of Health Research |
| Ebrahim 2020 [39] | USA | General population (COVID-19 daily cases for 1,320 large counties in 50 states, March-July, 2020) | Counties with workplace closure (vs. counties with no workplace closure) | Weekly growth rate decreased from the week before workplace closure to 14 days following closure (p=0.004) | Not reported | Decreased 14 days following workplace closure (p<0.001) | Not reported | Serious (cohort) | National Science Foundation, National Institutes of Health |
| Esra 2020  [40] | 26 countries and 34 US states where COVID-19 exceeded 5,000 cases by May 31, 2020 | General population (COVID-19 daily cases up to May 31, 2020) | Lockdown) (vs. no lockdown) | Not reported | Not reported | Daily reproduction number decreased 23% (95% CI = 18, 27) from the day reported cases exceeded 50 to May 31, 2020 | Not reported | Serious (cohort) | European Union's Horizon 2020, Swiss Excellence Foreign Scholar Grant. Swiss National Science Foundation Grants. President's Emergency Plan for AIDS Relief through USAID |
| Fisher 2020  [41] | USA | Symptomatic adults tested for SARS-CoV-2 in outpatient testing or health care centers in 10 states, July 1-29, 2020 (120 cases and 128 controls) | Working from home or teleworking (vs. going to an office or school setting) | Working from home or teleworking during the 2 weeks before illness onset was associated with a lower risk of COVID-19 illness (adjusted odds ratio = 0.48, 95% CI = 0.28, 0.77) | Not reported | Not reported | Not reported | Serious (case-control) | Not reported |
| Gokmen 2021 [42] | France, Spain, China, South Korea | General population (COVID-19 daily cases. First case in each country to June 1, 2020) | Lockdown (vs. no lockdown).  Workplace closure (vs. no closure) | Lockdown reduced daily growth rate by 38.3 percentage points (95% CI = 22.5, 54.0)  Workplace closure reduced daily growth rate by 53.7 percentage points (95% CI = 31.38, 76.04) | Not reported | Not reported | Not reported | Serious (cohort) | None |
| Guzzetta 2021 [43] | Italy | General population (March 10-April 15, 2020) | Lockdown from March 11, 2020 to May 11, 2020 (vs. no lockdown) | Not reported | Not reported | Decreased from 2.03 (95% CI = 1.94, 2.13) the day before the lockdown to 0.88 (95% CI = 0.84, 0.91) 14 days later. It remained stable at 0.76 (95% CI = 0.67, 0.85) for the subsequent 3 weeks during the lockdown | Not reported | Serious (before after) | European Commission, VRT Foundation |
| Haug 2020  [44] | 55 countries and 24 US states | General population | Lockdown, implemented in March-April 2020 (vs. no lockdown) | Not reported | Not reported | Significantly reduced  (-.008 to -0.14) | Not reported | Moderate (case control) | Austrian Science Promotion Agency, WWTF , Medizinisch-Wissenschaftlichen Fonds des Bargermeisters der Bundeshauptstadt Wien and the project VET-Austria |
| Herstein 2021 [45] | USA | Workers in 11 meat processing facilities in Nebraska (April 1-July 31, 2020) | Physical barriers (vs. no physical barriers) | 8 of 11 facilities showed a statistically significant reduction in COVID-19 incidence (p<0.001) | Not reported | Not reported | Not reported | Serious (before after) | Not stated |
| Islam 2020  [46] | 149 countries | General population (COVID-19 daily cases, March 24-May 30, 2020) | Lockdown (vs. none) | COVID-19 incidence post-lockdown vs. pre-lockdown decreased by 13% (incidence rate ratio = 0.87, 95% CI = 0.85, 0.89) | Not reported | Not reported | Not reported | Moderate (interrupted time series) | University of Oxford |
| Koh 2020 [47] | 142 countries that reported at least 100 cases as of May 28, 2020 | General population (first reported case to May 28, 2020) | Lockdown (vs. none) | Not reported | Not reported | Lockdown in place on the date of the 100^th^ case reduced the reproduction number over the 14 days since the date of the 100^th^ case by 0.32 (95% CI = -0.55, -0.09) | Not reported | Moderate (cohort) | None |
| Lau 2021 [48] | China | General population (COVID-19 daily cases, January 20-February 7, 2020) | Lockdown (vs. no lockdown) | Not reported | Increased from 1.9 days (95% CI = 1.4, 2.6) before lockdown (January 20-25) to 3.9 days (95% CI = 3.5, 4.3) during lockdown (January 26-February 7) | Not reported | Not reported | Serious (before after) | Not stated |
| Li 2021 [49] | 131 countries | General population (January 1 to July 20, 2020) | Lockdown (vs. none)  Workplace closure (vs. no closure) | Not reported | Not reported | Lockdown reduced the reproduction number 14 days after lockdown by 11% (R ratio = 0.89, 95% CI = 0.79, 1.00) (not significant)  Workplace closure reduced reproduction number 14 days after workplace closure by 11% (R ratio = 0.89, 95% CI = 0.78, 1.02) (not significant) | Not reported | Serious (before after) | Wellcome Trust |
| Li 2021b [50] | USA | General population (COVID-19 daily cases and deaths in US states, March-July, 2020) | Lockdown (vs. no lockdown)  Workplace closure (vs. no closure) | Lockdown showed a 4.79 percentage point reduction in growth rate by the fourth week (p<0.05)  Workplace closure resulted in a 7.52 percentage reduction in growth rate by the fourth week (p<0.05) | Not reported | Not reported | Lockdown showed a 6.53 percentage point reduction in growth rate after 2 months (p<0.05).  Workplace closure showed a 1.68 percentage point reduction in growth rate after 2 months (p>0.05) | Moderate (controlled before after) | National Science Foundation |
| Lin 2020 [51] | USA | General population (COVID-19 daily cases for 50 states and Washington DC, March 1-April 25, 2020) | Lockdown (vs. no Lockdown) | Growth rate significantly lower 26-30 days later (daily change in log of confirmed Covid cases =  -0.15, 95% CI = -0.29 to -0.01) | Not reported | Not reported | Not reported | Moderate (interrupted time series) | Not stated |
| Liu 2021 [52] | 130 countries | General population (January-June, 2020) | Lockdown (vs. no lockdown)  Workplace closure (vs. no closure) | Not reported | Not reported | Lockdown was significant in some but not all models.  Workplace closure significantly reduced the reproduction number | Not reported | Critical (cohort) | National Institute of Health Research, Bill & Melinda Gates Foundation |
| Lyu 2020 [53] | USA | General population (COVID-19 daily cases in 8 counties in Iowa and 7 bordering counties in Illinois, March 15-April 20, 2020) | 7 Illinois counties with lockdown (vs. 8 Iowa counties with no lockdown) | Cases reduced by 4.71 per 10,000 residents within 30 days (SE = 1.99, 95% CI = -8.64, -0.78, p=0.02). This represents a 30% reduction in cases | Not reported | Not reported | Not reported | Moderate (controlled before after) | Not stated |
| Lyu 2020b [54] | USA | General population (COVID-19 daily deaths for 47 states and Washington DC, March 21-May 15, 2020) | Lockdown in 42 states and DC (vs. no lockdown in 5 states) | Not reported | Not reported | Not reported | Daily growth rate declined by 6.1 percentage points (95% CI: -2.7, -9.9) 42 days after enactment of lockdown | Moderate (controlled before after) | Not stated |
| Padalabalanarayanan 2020  [55] | USA | General population (COVID-19 daily cases and deaths in 49 states and Washington DC, March 1-May 4, 2020) | Lockdown (vs. no lockdown) | Case rates reduced (beta = -1.166; 95% CI = -1.484, -0.847; p<.001) | Not reported | Not reported | Death rates reduced (beta = -0.204; 95% CI = -0.294, -0.113; p<.001) | Moderate (cohort) | Not stated |
| Saez 2020  [56] | Spain | General population (COVID-19 daily cases, January 17-April 5, 2020) | Lockdown (vs. no lockdown) | Cases decreased by 3.06 percentage points daily on average (95% credibility interval = -5.37, -0.88) | Not reported | Not reported | Not reported | Moderate (interrupted time series) | None |
| Salvatore 2020 [57] | India | General population (March 19-May 31, 2020) | Lockdown (vs. no lockdown) | Not reported | Increased from 3.56 days on March 24 to 14.37 days on May 31, 2020 | Decreased from 3.36 days (95% CI = 3.03, 3.71) on March 24 to 1.27 (95% CI = 1.26, 1.28) on May 31, 2020 | Not reported | Serious (before after) | University of Michigan, National Cancer Institute |
| Santamaria 2020 [58], Santamaria 2021 [59] | Spain | General population (COVID-19 daily cases and deaths, February 1-May 20, 2020) | Lockdown (vs. no lockdown) | Growth rate significantly decreased | Not reported | Lockdown resulted in a swift significant decrease until reaching values <1 | Growth rate significantly decreased | Serious (before after) | None |
| Saul 2020 [60] | Australia | General population (COVID-19 daily cases in Melbourne, June 14-July 30, 2020) | Lockdown (vs. no lockdown) | Growth rate decreased from 0.140 per day (SE = 0.008) to 0.038 per day (SE = 0.009) (p<0.001) | Not reported | Not reported | Not reported | Moderate (interrupted time series) | Not stated |
| Schroder 2021 [61] | South Africa | General population (COVID-19 daily cases, March 1-April 16, 2020) | Lockdown (vs. no lockdown) | Growth rate decreased from 0.27 to 0.038 (statistical significance not reported) | Increased from 2.5 days to 18 days | Not reported | Not reported | Moderate (interrupted time series) | German National Science Foundation |
| Silva 2020 [62] | Brazil | General population (COVID-19 daily cases and deaths, April 1-June 13, 2020) | Lockdown (vs. no lockdown) | Daily cases decreased in Sao Luis (beta = -0.09, SE = 0.02), Recife (beta = -0.11, SE = 0.01), Belem (beta = -0.13, SE = 0.02, and Fortaleza (beta = -0.07, SE = 0.01) | Not reported | Not reported | Daily deaths decreased in Sao Luis (beta = -0.13, SE = 0.02), Recife (beta = -0.06, SE = 0.01), Belem (beta = -0.10, SE = 0.02, and Fortaleza (beta = -0.09, SE = 0.02) | Moderate (interrupted time series) | Not stated |
| Singh 2020 [63] | India | General population (March 22-July 31, 2020) | Lockdown (vs. no lockdown) | Not reported | Not reported | Decreased over a period of 10 weeks from 2.78 (95% CI = 2.65, 2.91) to 1.27 (95% CI = 1.26, 1.27) | Not reported | Serious (before after) | Not stated |
| Singh 2021 [64] | USA | General population (January 1-June 3, 2020) | Workplace closure (vs. none) | Growth rate decreased by 1.26 percentage points (SE = 0.42) | Not reported | Not reported | Not reported | Moderate (controlled before after) | Not stated |
| Thayer 2021 [65] | India | General population (COVID-19 daily cases, March 2-September 1, 2020) | Lockdown (vs. no lockdown) | Growth rate decreased over a period of 8 weeks by 11% (95% CI = 9, 12) | Not reported | Not reported | Not reported | Moderate (interrupted time series) | None |
| Tobias 2020 [66] | Italy, Spain | General population (COVID-19 daily cases and deaths, February 24-April 5, 2020) | Lockdown (vs. no lockdown) | Daily percent increase in Italy declined from 21.6% (95% C = 16.2, 27.1) before lockdown to -2.0% (95% CI = -3.1, -0.9) during lockdown  Daily percent increase in Spain declined from 38.5% (95% CI = 27.0, 50.0) before lockdown to -2.7% (95% CI = -7.3, -1.9) during lockdown | Not reported | Not reported | Daily percent increase in Italy declined from 32.8% (95% CI: 21.0, 44.6) before lockdown to -0.2% (95% CI = -1.5, -1.0). Daily percent increase in Spain declined from 59.3% (95% CI = 23.0, 95.2) before lockdown to -1.8% (95% CI = -5.0, -3.1) | Moderate (interrupted time series) | Not stated |
| White 2020 [67] | USA | General population (COVID-19 daily cases, February 29-May 1, 2020) | Lockdown (yes vs. no)  Workplace closure (yes vs. no)  Restriction of restaurant operations (yes vs. no) | Not reported | Lockdown and workplace closure increased doubling time but not significantly.  Restricting restaurant operations significantly increased doubling time (p<0.01) | Not reported | Not reported | Moderate (cohort) | National Institutes of Health |
| Wong 2020 [68] | 54 countries and four epicenters | General population (COVID-19 daily cases, January 23-June 20, 2020) | Lockdown (vs. no lockdown) | Percent increase in daily cases was 26.9% (95% CI = 25.7, 28.0) before lockdown and 4.03% (95% CI = 3.96, 4.10) 30 days after lockdown began | Not reported | Not reported | Not reported | Moderate (interrupted time series) | None |
| Xu 2020 [69] | USA | General population (COVID-19 daily cases and deaths in 50 states and Washington DC, March 1-April 20, 2020) | Lockdown, from March 19, 2020 (vs. no order) | There was a turning point in daily new-case trend on March 28 (slope change = -0.09, 95% CI = -0.17, -0.01) | Not reported | Not reported | There was a turning point in daily new-death trend on April 9 (slope change = -0.06, 95% CI: -10, -0.02) | Moderate (interrupted time series) | National Institutes of Health |
| Zhang 2020 [70] | USA | General population (COVID-19 daily cases for 50 states, March 1-June 22, 2020) | Lockdown (vs. no lockdown) | Daily growth rate decreased significantly from 14.35% to 1.38% | Not reported | Not reported | Not reported | Moderate (controlled before after) | US Department of Agriculture, Cornell University |

Abbreviations, CI: Confidence interval; SD: Standard deviation; SE: Standard error

^a^Overall risk of bias, assessed using the ROBINS-I tool, is categorized as Low, Moderate, Serious, or Critical

## Table S5. Characteristics and results of studies assessing effect of lockdowns, January 1, 2020-May 11, 2021: Anxiety and depression symptoms

| **First author, year published** | **Country (outcome data source)** | **Population in whom outcomes assessed**  **(sample size)** | **Intervention (comparator)** | **Anxiety**  **[Measurement instrument]** | **Depression**  **[Measurement instrument]** | **Overall risk of bias (study design)^a^** | **Funding source** |
| --- | --- | --- | --- | --- | --- | --- | --- |
| Badellino, 2022  (published online in 2021)  [71] | Argentina (online survey disseminated in social media and by email) | Persons ages 18 years and older (1,985 respondents in March 29-April 12 and 2,839 in May 23-June 12, 2020) | Lockdown, from March to June 12, 2020 (vs. no lockdown) | Not reported | Prevalence of moderate or severe depression increased from 24.3% to 47.8%, p<0.000.  [PHQ-9] | Critical (before after) | None |
| Barone Gibbs, 2021 [72] | USA (longitudinal survey of desk workers enrolled in a blood pressure trial) | Desk workers with elevated, untreated blood pressure, ages 21-65 years, in Pittsburgh (111 respondents in December 2019 and 103 in May-June, 2020) | Lockdown, from March to May, 2020 (vs. no lockdown) | Not reported | Mean depression score increased from 2.2 (SD = 3.2) in December 2019 to 3.5 (SD = 4.3) in May-June 2020 (change of 1.5 (SD = 3.9), p<0.001).  [Short Form (SF)-36] | Serious (before after) | National Institutes of Health |
| Canet-Juric, 2020 [73] | Argentina (longitudinal online survey distributed in social media) | Persons ages 18 years and older in Argentina, (6,057 persons participated in both the March 22-25 and April 3-9 surveys) | Lockdown, from March 20 to June 12, 2020 (vs. no lockdown) | Mean state-anxiety score decreased from 1.16 (SD = 0.50) to 1.11 (SD = 0.51) (p<0.001).  [State-Trait Anxiety Inventory] | Mean depression score increased from 8.74 (SD = 7.41) to 9.41 (SD = 7.88) (p<0.001).  [Beck Depression Inventory-11] | Critical (before after) | National Scientific and Technical Research Council |
| Castellini, 2021 [74] | Italy (longitudinal online survey of persons recruited using convenience and snowball sampling) | Persons ages 18-60 years residing in Tuscany, Italy, (130 persons participated in both the December 1,2019-January 15, 2020 and April 22-May 3, 2020 surveys) | Lockdown, from March 9, 2020 (vs. no lockdown) | Mean phobic anxiety score increased from 0.26 (SD = 0.43) to 0.48 (SD = 0.63), p<0.001.  [BSI] | Mean depression score increased from 0.57 (SD = 0.48) to 0.73 (SD = 0.65), p=0.003.  [BSI] | Critical (before after) | None |
| Cecchini, 2021 [75] | Spain (longitudinal survey of persons recruited using social media and snowball sampling) | Persons ages 18-84 years (the number of participants were 595 in March 18-24 and 205 in April 17-23, 2020) | Lockdown, from March 14 to May 4, 2020 (vs. no lockdown) | Not reported | Depression increased from March 18-24 to April 17-23 (odds ratio = 2.93, 95% CI = 1.97, 4.38).  [Kandel and Davis scale] | Critical (before after) | Not stated |
| Fancourt, 2021 [76] | UK (longitudinal survey of persons recruited using snowballing sampling, recruitment companies, and partnerships with third sector organizations) | Adults living in England (36,520 adults with at least three repeated measures between March 23 and August 9, 2020) | Lockdown, from March 23 to May 10, 2020 (vs. no lockdown) | Mean anxiety score in week 1 was 5.7 (SD = 5.6). It declined across the first 20 weeks after implementation of lockdown (beta = -1.93, SE: 0.26, p<0.0001).  [GAD-7] | The mean depression score was 6.6 (SD = 6.0) in week 1. It declined across the first 20 weeks after implementation of lockdown (beta = -2.52, SE = 0.28, p<0.0001).  [PHQ-9] | Serious (before after) | Nuffield Foundation, UK Research and Innovation, Wellcome Trust |
| Gonzalez-Sanguino 2021 [77] | Spain (longitudinal survey of adults recruited through databases of different institutions and social media) | Persons ages 18 years and older (3,480 participants in March 21-29 and 1,041 in April 13-27, 2020) | Lockdown, from March 30 to April 12, 2020 (vs. no lockdown) | No significant change in anxiety score (mean = 1.79, SD = 1.63 vs. mean = 1.80, SD = 1.57, p=0.99).  [GAD-21] | Mean depression score increased from 1.60 (SD = 1.51) to 1.81 (SD = 1.43), p<0.001.  [PHQ-2] | Critical (before after) | None |
| Gopal 2020 [78] | India (longitudinal survey of adults recruited through social media and social networks of the authors) | Adults living in India (159 adults participated during the March 29-April 13 and May 24-June 6 survey rounds) | Lockdown, from March 25 to May 3, 2020 (vs.no lockdown) | Anxiety score increased (regression beta = 0.814, 95% CI = 0.025, 1.602).  [GAD-7] | Depression score increased (regression beta = 0.367, 95% CI = 0.130, 0.604).  [PHQ-4] | Critical (before after) | None |
| Hyland 2021 [79] | Ireland (longitudinal survey of adults recruited from existing online, nationally representative panels of survey participants) | Adults living in Ireland (1,041 participants in March 31-April 5 and 506 in April 30-May 14, 2020) | Lockdown, from March 31 to May 14, 2020 (vs. no lockdown) | No change in generalized anxiety disorder (20.0%, 95% CI = 17.6, 22.4 vs. 17.4%, 95% CI = 14.5, 20.4, p=0.083).  [GAD-7] | No change in major depression (22.8%, 95% CI = 20.2, 25.3 vs. 24.2, 95% CI = 20.9, 27.6, p=0.368).  [PHQ-9] | Critical (before after) | None |
| Le 2021 [80] | USA (data from the Census Bureau's Household Pulse Survey) | Adults in 50 states and Washington DC (166,037 respondents in April 23-May 5 and 151,555 respondents in May 21-26, 2020) | Lockdown, timing varied across states from March 19 to June 10, 2020 (vs. no lockdown) | No significant change in anxiety score (beta = 0.005, SE = 0.004).  [Instrument is unclear] | Depression increased significantly (beta = 0.008, SE = 0.002).  [Instrument is unclear] | Critical (controlled before after) | Not stated |
| Mergel 2021 [81] | Germany (longitudinal survey of adults recruited from an existing research study) | Adults ages 18-65 years with no mental disorder living in Dresden (49 adults completed surveys in March 23-April 20, 2020 and June 22-July 19, 2020 | Lockdown, from March 22 to April 17, 2020 (vs. no lockdown) | No significant change in anxiety score (mean = 0.27, SD = 0.30 vs. mean = 0.27, SD = 0.30).  [BSI-18] | No significant change in depression score (mean = 0.41, SD = 0.50 vs. mean = 0.33, SD = 0.50).  [BSI-18] | Critical (before after) | Not stated |
| O'Connor 2021  (published online in 2020)  [82] | UK (longitudinal survey of adults of an existing online UK panel were invited to take part in an online survey of health and well-being) | Adults ages 18 years and older (3,077 in March 31-April 9, 2020, and 2,604 in April 28-May 11, 2020) | Lockdown, from March 23, 2020 (vs.no lockdown) | Proportion with anxiety decreased. 21.0% (95% CI = 19.6, 22.4) vs. 16.8% (95% CI = 15.4, 18.2).  [GAD-7] | No significant change in depression. 26.1% (95% CI = 24.6, 27.7) vs. 23.7% (95% CI = 22.1, 25.3).  [PHQ-9] | Serious (before after) | University of Glasgow, Samaritans, Scottish Association for Mental Health, Mindset Foundation |
| Ozamiz-Etxebarria 2020 [83] | Spain (adults recruited through snowball sampling) | Adults ages 18 to 82 years in Basque (1,112 respondents in March 11-18, 2020 and 881 in April 2-12, 2020) | Lockdown, from March 14, 2020 (vs. no lockdown) | Mean anxiety score increased from 2.30 (SD = 2.93) to 2.86 (SD = 3.47), p<0.001.  [DASS-21] | Mean depression score increased from 2.8 (SD = 3.57) to 4.71 (SD = 4.25), p<0.001.  [DASS-21] | Critical (before after) | KideOn Research Group of the Basque Government |
| Roma 2020 [84] | Italy (Longitudinal survey of adults residing in Italy recruited through social networks) | Adults ages 18 years and older (439 adults participated in both the initial survey in March 18-22, 2020 and a follow-up survey in April 28-May 3, 2020) | Lockdown, from March 9, 2020 (vs. no lockdown) | No significant change in anxiety score (mean = 3.14, SD = 3.95 vs. mean = 3.44, SD = 4.14, p=0.08).  [DASS-21] | Mean depression score increased from 5.50 (SD = 4.88) to 6.79 (SD = 5.40), p<0.001.  [DASS-21] | Serious (before after) | None |
| Somma 2021 [85] | Italy (longitudinal survey of adults recruited through the web and social media) | Adults ages 18 years and older (304 participants during the first week of lockdown and after 2 months at the end of the lockdown) | Lockdown, from March 9, 2020 (vs. no lockdown) | Mean anxiety score decreased from 57.47 (SD = 7.56) to 54.27 (SD = 9.68).  [PROMIS Anxiety Short Form] | Mean depression score decreased from 53.18 (SD = 7.48) to 51.32 (SD = 9.17).  [PROMIS Depression Short Form] | Serious (before after) | None |

Abbreviations, BSI: Brief Symptom Inventory; DASS: Depression Anxiety and Stress Scale; GAD: Generalized Anxiety Disorder Scale; PHQ: Patients Health Questionnaire; SD: Standard deviation; SE: Standard error

^a^Overall risk of bias, assessed using the ROBINS-I tool, is categorized as Low, Moderate, Serious, or Critical

## Table S6. Characteristics and results of studies assessing effect of lockdowns, January 1, 2020-May 11, 2021: Unemployment and labor force participation

| **First author, year published** | **Country (outcome data source)** | **Population in whom outcomes assessed (sample size)** | **Intervention (comparator)** | **Unemployment** | **Labor force participation** | **Overall risk of bias (study design)^a^** | **Funding source** |
| --- | --- | --- | --- | --- | --- | --- | --- |
| Hoehn-Velasco, 2021 [7] | Mexico (Mexican Social Security Institute administrative data) | Census of all formal employees in the private sector, excluding informal workers not affiliated with social security (the total labor force is 55 million, and about 60% are informal workers). | Lockdown, March 23-May 30, 2020 (vs. no lockdown) | Employment decreased by 5.4 percentage points (SE = 0.9, p<0.01) from March to November 2020 | Not reported | Moderate (controlled before after) | Not stated |
| Beland, 2020 [86] | USA (Current Population Survey, January 2016-April 2020) | Civilian non-institutional population ages 16-70 years (60,000 households surveyed each month) | Lockdown, began on March 19, 2020 (vs. no lockdown) | Monthly unemployment was 4.4%, and it increased by 3.773 percentage points (SE = 0.765) from February to April 2020 | Labor force participation was 70.5%, and it decreased by 2.47 percentage points (SE = 0.43) from February to April 2020 | Moderate (controlled before after) | Not stated |
| Churchill, 2020 [87]^b^ | Australia (Labor Force Survey) | 15-64 year olds (26,000 households surveyed each month) | Lockdown, began in March 2020 (vs. no lockdown) | Unemployment increased from 5.9% in February 2020 to 7.5% in June 2020 | Decreased from 78.8% in February 2020 to 75.8% in June | Serious (before after) | Not stated |
| Coibion, 2020 [88] | USA (Kilts Nielsen Consumer Panel Survey) | Households (18,344 respondents in January 2020 and 13,771 in April 2020) | Lockdown (vs. no lockdown) | Unemployment increased by 2.4 percentage points (SE = 0.9, p<0.05) from January to April 2020 | Decreased by 1.9 percentage points (SE = 0.9, p<0.05) from January to April 2020 | Serious (controlled before after) | Not stated |
| Robinson, 2021 [89] | USA (Understanding America Study) | Adults ages 18 years and older (5,549 respondents in March 2020 and 5,784 in May 27-June 9, 2020) | Lockdown, from March 19, 2020 (vs. no lockdown) | Persons not employed increased from March 10-18 to May 27-June 9, 2020 by 9.15 percentage points (95% CI = 7.61, 10.69, p<0.001) | Not reported | Serious (before after) | Bill & Melinda Gates Foundation, National Institute on Aging |

Abbreviations, CI: Confidence interval; SE: standard error

^a^Overall risk of bias, assessed using the ROBINS-I tool, is categorized as Low, Moderate, Serious, or Critical

^b^Labor force participation and unemployment rates by age and sex were converted to overall rates using Australia's population demographics (available at https://www.abs.gov.au/AUSSTATS/abs@.nsf/DetailsPage/3101.0Jun%202019?OpenDocument, Table 7)

## Table S7. Risk of bias assessment for studies, January 1, 2020–May 11, 2021: COVID-19 morbidity and mortality outcomes

| Author, Year | Risk of bias domains^a^ | | | | | | | Overall risk of bias^a^ |
| --- | --- | --- | --- | --- | --- | --- | --- | --- |
|  | Confounding | Selection of participants into the study | Classification of interventions | Deviations from intended interventions | Missing data | Measurement of outcome | Selection of the reported result |  |
| Alfano 2020 [30] | Serious | Low | Low | Moderate | Low | Low | Low | Serious |
| Askitas 2021 [31] | Moderate | Low | Low | Low | Low | Low | Low | Moderate |
| Castillo 2020 [32] | Serious | Low | Low | Low | Low | Low | Moderate | Serious |
| Chae 2020 [33] | Moderate | Low | Low | Low | Low | Low | Low | Moderate |
| Cobb 2020 [34] | Moderate | Low | Low | Low | Low | Low | Moderate | Moderate |
| Courtemanche 2020 [35] | Moderate | Low | Low | Low | Low | Low | Low | Moderate |
| Deb 2020 [36] | Moderate | Low | Low | Low | Low | Low | Low | Moderate |
| Dreher 2021 [37] | Moderate | Low | Low | Low | Low | Low | Low | Moderate |
| Duhon 2021 [38] | Serious | Low | Low | Low | No info | Low | Low | Serious |
| Ebrahim 2020 [39] | Serious | Low | Moderate | Moderate | Low | Low | Moderate | Serious |
| Esra 2020 [40] | Serious | Low | Low | Low | No info | Low | Low | Serious |
| Fisher 2020 [41] | Moderate | Low | Moderate | Low | Moderate | Low | Low | Serious |
| Gokmen 2021 [42] | Serious | Low | Low | Low | Low | Low | Low | Serious |
| Guzzetta 2021 [43] | Serious | Low | Low | Moderate | Low | Low | Low | Serious |
| Haug 2020 [44] | Moderate | Low | Low | Low | No info | Low | Low | Moderate |
| Herstein 2021 [45] | Serious | Low | Low | Moderate | Low | Low | Low | Serious |
| Islam 2020 [46] | Moderate | Low | Low | Moderate | Low | Low | Low | Moderate |
| Koh 2020 [47] | Moderate | Low | Low | Low | Low | Low | Low | Moderate |
| Lau 2021 [48] | Serious | Low | Low | Low | Low | Low | Low | Serious |
| Li 2021 [49] | Serious | Low | Low | Low | Low | Low | Low | Serious |
| Li 2021b [50] | Moderate | Low | Low | Low | Low | Low | Low | Moderate |
| Lin 2020 [51] | Moderate | Low | Low | Low | Low | Low | Low | Moderate |
| Liu 2021 [52] | Critical | Low | Low | Low | Low | Low | Low | Critical |
| Lyu 2020 [53] | Moderate | Low | Low | Low | Low | Low | Low | Moderate |
| Lyu 2020b [54] | Moderate | Low | Low | Low | Low | Low | Low | Moderate |
| Padalabalanarayanan 2020 [55] | Moderate | Low | Low | Moderate | Low | Low | Low | Moderate |
| Saez 2020 [56] | Moderate | Low | Low | Low | Low | Low | Moderate | Moderate |
| Salvatore 2020 [57] | Serious | Low | Low | Low | Low | Low | Low | Serious |
| Santamaria 2020 [58], Santamaria 2021 [59] | Serious | Low | Low | Low | Low | Low | Low | Serious |
| Saul 2020 [60] | Moderate | Low | Low | Low | Low | Low | Low | Moderate |
| Schroder 2021 [61] | Moderate | Low | Low | Low | Low | Low | Low | Moderate |
| Silva 2020 [62] | Moderate | Low | Low | Low | Low | Low | Low | Moderate |
| Singh 2020 [63] | Serious | Low | Low | Low | Low | Low | Low | Serious |
| Singh 2021 [64] | Moderate | Low | Low | Low | Low | Low | Low | Moderate |
| Thayer 2021 [65] | Moderate | Low | Low | Low | Low | Low | Low | Moderate |
| Tobias 2020 [66] | Moderate | Low | Low | Low | Low | Moderate | Low | Moderate |
| White 2020 [67] | Moderate | Low | Low | Low | Low | Low | Low | Moderate |
| Wong 2020 [68] | Moderate | Low | Low | Low | Low | Low | Moderate | Moderate |
| Xu 2020 [69] | Moderate | Low | Low | Low | Low | Low | Moderate | Moderate |
| Zhang 2020 [70] | Moderate | Low | Low | Low | Low | Low | Low | Moderate |

^a^Risk of bias domains and overall risk of bias, assessed using the ROBINS-I tool, are categorized as Low, Moderate, Serious, or Critical

## Table S8. Risk of bias assessment for studies, January 1, 2020–May 11, 2021: Anxiety and depression symptoms

| Author, Year | Risk of bias domains^a^ | | | | | | | Overall risk of bias^a^ |
| --- | --- | --- | --- | --- | --- | --- | --- | --- |
|  | Confounding | Selection of participants into the study | Classification of interventions | Deviations from intended interventions | Missing data | Measurement of outcome | Selection of the reported result |  |
| Badellino 2022  [71] | Critical | Moderate | Low | Low | Low | Low | Low | Critical |
| Barone Gibbs 2021 [72] | Serious | Low | Low | Low | Low | Low | Low | Serious |
| Canet-Juric 2020 [73] | Serious | Serious | Low | Low | Serious | Low | Low | Critical |
| Castellini 2021 [74] | Serious | Low | Low | Low | Moderate | Low | Low | Critical |
| Cecchini 2021 [75] | Serious | Moderate | Low | Low | Serious | Low | Low | Critical |
| Fancourt 2021 [76] | Serious | Serious | Low | Low | Low | Low | Low | Serious |
| Gonzalez-Sanguino 2021 [77] | Serious | Low | Low | Low | Serious | Low | Low | Critical |
| Gopal 2020 [78] | Serious | Moderate | Low | Low | Serious | Low | Low | Critical |
| Hyland 2021 [79] | Serious | Serious | Low | Low | Serious | Low | Low | Critical |
| Le 2021 [80] | Moderate | Moderate | Low | Low | Critical | Low | Low | Critical |
| Mergel 2021 [81] | Serious | Serious | Low | Low | Serious | Low | Low | Critical |
| O'Connor 2021  [82] | Serious | Serious | Low | Low | Moderate | Low | Low | Serious |
| Ozamiz-Etxebarria 2020 [83] | Serious | Moderate | Low | Low | Serious | Low | Low | Critical |
| Roma 2020 [84] | Serious | Moderate | Low | Low | Moderate | Low | Low | Serious |
| Somma 2021 [85] | Serious | Serious | Low | Low | Low | Low | Low | Serious |

^a^Risk of bias domains and overall risk of bias, assessed using the ROBINS-I tool, are categorized as Low, Moderate, Serious, or Critical

## Table S9. Risk of bias assessment for studies, January 1, 2020–May 11, 2021: Unemployment and labor force participation

| Author, Year | Risk of bias domains^a^ | | | | | | | Overall risk of bias^a^ |
| --- | --- | --- | --- | --- | --- | --- | --- | --- |
|  | Confounding | Selection of participants into the study | Classification of interventions | Deviations from intended interventions | Missing data | Measurement of outcome | Selection of the reported result |  |
| Hoehn-Velasco 2021 [5] | Moderate | Low | Low | Low | Low | Low | Low | Moderate |
| Beland 2020 [86] | Moderate | Low | Low | Low | Low | Low | Low | Moderate |
| Churchill 2020 [87] | Serious | Low | Low | Low | Low | Low | Low | Serious |
| Coibion 2020 [88] | Moderate | Low | Low | Low | Serious | Low | Low | Serious |
| Robinson 2021 [89] | Serious | Low | Low | Low | Low | Low | Low | Serious |

^a^Risk of bias domains and overall risk of bias, assessed using the ROBINS-I tool, are categorized as Low, Moderate, Serious, or Critical

## Table S10. Studies excluded from the review and reasons for exclusion, January 1, 2020–May 11, 2021

| Title | Authors | Year | Journal | Vol. | Issue | Pages | Exclusion reason |
| --- | --- | --- | --- | --- | --- | --- | --- |
| The role of leisure crafting for emotional exhaustion in telework during the COVID-19 pandemic | Abdel Hadi, S.; Bakker, A. B.; Hausser, J. A. | 2021 | Anxiety, Stress, & Coping | |  | 15-Jan | Wrong comparator |
| Singapore's Responses to the COVID-19 Outbreak: A Critical Assessment | Abdullah, W. J.; Kim, S. | 2020 | American Review of Public Administration | | | | Wrong study design |
| COVID-19 in the gaza strip and the west bank under the political conflict in Palestine | Abed, Y. | 2020 | South Eastern European Journal of Public Health | 14 |  | 12-Jan | Wrong study design |
| SARS-CoV-2 Spread Dynamics in Italy: The Calabria Experience | Abenavoli, L.; Cinaglia, P.; Procopio, A. C.; et al | 2021 | Reviews on Recent Clinical Trials | 1 |  | 1 | Wrong study design |
| Optimal Targeted Lockdowns in a Multi-Group SIR Model | Acemoglu, D; Chernozhukov, V; Werning, I; Whinston, M.D. | 2020 |  |  |  |  | Wrong outcome |
| Reduction in air pollution and attributable mortality due to COVID-19 lockdown | Achebak, H.; Petetin, H.; Quijal-Zamorano, M.; Bowdalo, D.; Garcia-Pando, C. P.; Ballester, J. | 2020 | The lancet. Planetary Health | 4 | 7 | e268 | Wrong study design |
| Modeling behavioral change and COVID-19 containment in Mexico: A trade-off between lockdown and compliance | Acuna-Zegarra, M. A.; Santana-Cibrian, M.; Velasco-Hernandez, J. X. | 2020 | Mathematical Biosciences | 325 |  | 108370 | Wrong intervention |
| Work Tasks That Can Be Done From Home: Evidence on the Variation Within and Across Occupations and Industries | Adams-Prassl, A; Boneva, T; Golin, M; Rauh, C | 2020 |  |  |  |  | Wrong outcome |
| Inequality in the Impact of the Coronavirus Shock: New Survey Evidence for the UK | Adams-Prassl, A; Boneva, T; Golin, M; Rauh, C | 2020 |  |  |  |  | Wrong comparator |
| The Impact of the Coronavirus Lockdown on Mental Health: Evidence from the US | Adams-Prassl, A; Boneva, T; Golin, M; Rauh, C | 2020 |  |  |  |  | Wrong outcome |
| Interplay of global multi-scale human mobility, social distancing, government interventions, and COVID-19 dynamics | Adiga, A.; Wang, L.; Sadilek, A.; et al | 2020 | MedRxiv : the Preprint Server for Health Sciences | 11 |  | 11 | Wrong comparator |
| An analysis of Covid-19 implications for SMEs in Pakistan | Aftab, R.; Naveed, M.; Hanif, S. | 2021 | Journal of Chinese Economic and Foreign Trade Studies | 14 | 1 | 74-88 | Wrong intervention |
| COVID-19: Current pandemic and its societal impact | Agarwal, V.; Sunitha, B. K. | 2020 | International Journal of Advanced Science and Technology | 29 | 5 Special Issue | 432-439 | Wrong study design |
| Reproduction ratio and growth rates: Measures for an unfolding pandemic | Aguiar, M.; Van-Dierdonck, J. B.; Stollenwerk, N. | 2020 | PLoS ONE [Electronic Resource] | 15 | 7 | e0236620 | Wrong intervention |
| A Dynamic Structural Model of Virus Diffusion and Network Production: A First Report | Aguirregabiria, V; Gu, J; Luo, Y; Mira, P. S. | 2020 |  |  |  |  | Wrong study design |
| Evidence of initial success for China exiting COVID-19 social distancing policy after achieving containment | Ainslie, K. E. C.; Walters, C. E.; Fu, H.; et al | 2020 | Wellcome Open Research | 5 |  | 81 | Wrong intervention |
| The impact of COVID-19 related â€˜stay-at-homeâ€™ restrictions on food prices in Europe: findings from a preliminary analysis | Akter, S. | 2020 | Food Security | |  |  | Wrong outcome |
| Measurement Method for Evaluating the Lockdown Policies during the COVID-19 Pandemic | Al Zobbi, M.; Alsinglawi, B.; Mubin, O.; Alnajjar, F. | 2020 | International Journal of Environmental Research & Public Health [Electronic Resource] | 17 | 15 | 2 | Wrong study design |
| Coronavirus disease of 2019 (COVID-19) in the Gulf Cooperation Council (GCC) countries: Current status and management practices | Alandijany, T. A.; Faizo, A. A.; Azhar, E. I. | 2020 | Journal of Infection and Public Health | 13 | 6 | 839-842 | Wrong study design |
| A Comparative Study on the Strategies Adopted by the United Kingdom, India, China, Italy, and Saudi Arabia to Contain the Spread of the COVID-19 Pandemic | Alanezi, F.; Aljahdali, A.; Alyousef, S. M.; et al | 2020 | Journal of Healthcare Leadership | 12 |  | 117-131 | Wrong study design |
| A comparative study of strategies for containing the COVID-19 pandemic in Gulf Cooperation Council countries and the European Union | Alanezi, F.; Althumairi, A.; Aljaffary, A.; et al | 2021 | Informatics in Medicine Unlocked | 23 |  | 100547 | Wrong study design |
| Where are the missing emergencies? Lockdown and health risk during the pandemic | Ale-Chilet, J; Atal, J. P.; Dominguez, P | 2020 |  |  |  | 29 pages | Wrong outcome |
| Do Stay-at-Home Orders Cause People to Stay at Home? Effects of Stay-at-Home Orders on Consumer Behavior (REVISED May 2020) | Alexander, D; Karger, E | 2020 |  |  |  | 38-38 | Wrong outcome |
| Assessment of the Quality of Life during COVID-19 Pandemic: A Cross-Sectional Survey from the Kingdom of Saudi Arabia | Algahtani, F. D.; Hassan, S. U.; Alsaif, B.; Zrieq, R. | 2021 | International Journal of Environmental Research & Public Health [Electronic Resource] | 18 | 3 | 20 | Wrong comparator |
| Statistical analysis of COVID-19 pandemic across the provinces of Iraq | Ali Al-Suhail, R. G.; Ali, L. F. | 2021 | Iraqi Journal of Science | 62 | 3 | 811-824 | Wrong study design |
| Working from home facilities model during pandemic situation | Ali, I. M.; Hamid, M.; Zaidi, M.; Wan Hamdan, W. S.; Mohd Radzuan, N. A. | 2021 | Turkish Journal of Computer and Mathematics Education | 12 | 3 | 2683-2692 | Wrong outcome |
| Immediate impact of stay-at-home orders to control COVID-19 transmission on mental well-being in Bangladeshi adults: Patterns, Explanations, and future directions | Ali, M.; Ahsan, G. U.; Khan, R.; Khan, H. R.; Hossain, A. | 2020 | BMC Research Notes | 13 | 1 | 494 | Wrong comparator |
| My home is my castle â€“ The benefits of working from home during a pandemic crisis | Alipour, J. V.; Fadinger, H.; Schymik, J. | 2021 | Journal of Public Economics | 196 |  |  | Wrong intervention |
| Germany's Capacities to Work from Home | Alipour, Jean-Victor; Falck, Oliver; Schuller, Simone | 2020 |  |  |  |  | Wrong outcome |
| Health system's response to the COVID-19 pandemic in conflict settings: Policy reflections from Palestine | AlKhaldi, M.; Kaloti, R.; Shella, D.; Al Basuoni, A.; Meghari, H. | 2020 | Global Public Health | 15 | 8 | 1244-1256 | Wrong study design |
| Predicting cases and deaths in Europe from covid-19 tests and country populations | Allen, D. E.; McAleer, M. | 2021 | Annals of Financial Economics | | |  | Wrong study design |
| Substance use, mental health and weight-related behaviours during the COVID-19 pandemic in people with obesity | Almandoz, J. P.; Xie, L.; Schellinger, J. N.; Mathew, M. S.; et al | 2021 | Clinical Obesity | 11 | 2 | e12440 | Wrong intervention |
| Effects of the COVID-19 lockdown on urban mobility: Empirical evidence from the city of Santander (Spain) | Aloi, A.; Alonso, B.; Benavente, J.; et al | 2020 | Sustainability (Switzerland) | 12 | 9 |  | Wrong outcome |
| The Impact of COVID-19 on Gender Equality | Alon, T. M.; Doepke, M; Olmstead-Rumsey, J; Tertilt, M. | 2020 |  |  |  |  | Wrong study design |
| How Should Policy Responses to the COVID-19 Pandemic Differ in the Developing World? | Alon, T. M.; Kim, M; Lagakos, D; VanVuren, M. | 2020 |  |  |  |  | Wrong study design |
| Prediction of Epidemic Peak and Infected Cases for COVID-19 Disease in Malaysia, 2020 | Alsayed, A.; Sadir, H.; Kamil, R.; Sari, H. | 2020 | International Journal of Environmental Research & Public Health [Electronic Resource] | 17 | 11 | 8 | Wrong intervention |
| Seroprevalence of SARS-CoV-2 (COVID-19) among healthcare workers in Saudi Arabia: comparing case and control hospitals | Alserehi, H. A.; Alqunaibet, A. M.; Al-Tawfiq, J. A.; et al | 2021 | Diagnostic Microbiology & Infectious Disease | 99 | 3 | 115273 | Wrong setting |
| A Simple Planning Problem for COVID-19 Lockdown | Alvarez, Fernando; Argente, David; Lippi, Francesco | 2020 |  |  |  |  | Wrong study design |
| How the COVID-19 pandemic has changed our lives: A study of psychological correlates across 59 countries | Alzueta, E; Perrin, P; Baker, F. C.; et al | 2021 | Journal of Clinical Psychology | 77 | 3 | 556-570 | Wrong comparator |
| Mathematical Modelling to Assess the Impact of Lockdown on COVID-19 Transmission in India: Model Development and Validation | Ambikapathy, B.; Krishnamurthy, K. | 2020 | JMIR Public Health and Surveillance | 6 | 2 | e19368 | Wrong study design |
| On a Coupled Time-Dependent SIR Models Fitting with New York and New-Jersey States COVID-19 Data | Ambrosio, B.; Aziz-Alaoui, M. A. | 2020 | Biology | 9 | 6 | 24 | Wrong comparator |
| Mental health of Italian adults during COVID-19 pandemic | Amendola, S.; Spensieri, V.; Hengartner, M. P.; Cerutti, R. | 2021 | British Journal of Health Psychology | 26 | 2 | 644-656 | Wrong comparator |
| Assessment of Countries' Preparedness and Lockdown Effectiveness in Confronting COVID-19 | Amer, F.; Hammoud, S.; Farran, B.; Boncz, I.; Endrei, D. | 2020 | Disaster Medicine & Public Health Preparedness | | | 15-Jan | Wrong intervention |
| Psychological consequences of COVID-19 home confinement: The ECLB-COVID19 multicenter study | Ammar, A; Mueller, P; Trabelsi, K; et al | 2020 | PLoS ONE Vol 15(11), 2020, ArtID e0240204 | 15 | 11 |  | Wrong study design |
| Nigeria and Italy Divergences in Coronavirus Experience: Impact of Population Density | Amoo, E. O.; Adekeye, O.; Olawole-Isaac, A.; et al | 2020 | Thescientificworldjournal | 2020 |  | 8923036 | Wrong study design |
| Early adoption of non-pharmaceutical interventions and COVID-19 mortality | Amuedo-Dorantes, C.; Borra, C.; Rivera-Garrido, N.; Sevilla, A. | 2021 | Economics & Human Biology | 42 |  | 101003 | Wrong comparator |
| Coronavirus outbreak in Nigeria: Burden and socio-medical response during the first 100 days | Amzat, J.; Aminu, K.; Kolo, V. I.; et al | 2020 | International Journal of Infectious Diseases | 98 |  | 218-224 | Wrong study design |
| Consumer Responses to the COVID-19 Crisis: Evidence from Bank Account Transaction Data | Andersen, A; Hansen, E. T; Johannesen, N; Sheridan, A | 2020 |  |  |  |  | Wrong outcome |
| Effect of a Federal Paid Sick Leave Mandate on Working and Staying at Home: Evidence from Cellular Device Data | Andersen, M; Maclean, J. C; Pesko, M. F.; Simon, K. I. | 2020 |  |  |  |  | Wrong outcome |
| Developing a sustainable exit strategy for COVID-19: health, economic and public policy implications | Anderson, M.; McKee, M.; Mossialos, E. | 2020 | Journal of the Royal Society of Medicine | 113 | 5 | 176-178 | Wrong study design |
| Being a group fitness instructor during the covid-19 crisis: Navigating professional identity, social distancing, and community | Andersson, K.; Andreasson, J. | 2021 | Social Sciences | 10 | 4 |  | Wrong outcome |
| A Qualitative Study of Impacts of the COVID-19 Pandemic on Lives in Adults with Attention Deficit Hyperactive Disorder in Japan | Ando, M.; Takeda, T.; Kumagai, K. | 2021 | International Journal of Environmental Research & Public Health [Electronic Resource] | 18 | 4 | 21 | Wrong comparator |
| THE impact of COVID-19 on work force in information technology sector | Anjani, P. K.; Sundram, S.; Abinaya, V. | 2020 | European Journal of Molecular and Clinical Medicine | 7 | 2 | 3660-3674 | Wrong outcome |
| Covid-19: collateral damage of lockdown in India | Anonymous, | 2020 | BMJ | 369 |  | m1797 | Wrong study design |
| Coronavirus: share lessons on lifting lockdowns | Anonymous, | 2020 | Nature | 581 | 7806 | 8 | Wrong study design |
| COVID-19 and Bangladesh: Challenges and How to Address Them | Anwar, S.; Nasrullah, M.; Hosen, M. J. | 2020 | Frontiers in Public Health | 8 |  | 154 | Wrong study design |
| Are COVID fatalities in the US higher than in the EU, and if so, why? | Aparicio, A.; Grossbard, S. | 2021 | Review of Economics of the Household | | | 20-Jan | Wrong intervention |
| Deadly Debt Crises: COVID-19 in Emerging Markets | Arellano, Cristina; Bai, Yan; Mihalache, Gabriel | 2020 |  |  |  |  | Wrong outcome |
| Covid-19 lockdowns, income distribution, and food security: An analysis for South Africa | Arndt, C.; Davies, R.; Gabriel, S.; et al | 2020 | Global Food Security | 26 |  |  | Wrong outcome |
| Empirical assessment of government policies and flattening of the COVID19 curve | Arshed, N.; Meo, M. S.; Farooq, F. | 2020 | Journal of Public Affairs | |  | e2333 | Wrong outcome |
| Exploring the Socio-Economic Impact of COVID-19 Pandemic in Marketplaces in Urban Ghana | Asante, L. A.; Mills, R. O. | 2020 | Africa Spectrum | |  |  | Wrong study design |
| Lockdown Strategies, Mobility Patterns and COVID-19 | Askitas, N; Tatsiramos, K; Verheyden, B | 2020 |  |  |  |  | Wrong study design |
| Is the lockdown important to prevent the COVID-9 pandemic? Effects on psychology, environment and economy-perspective | Atalan, A. | 2020 | Annals of Medicine & Surgery | 56 |  | 38-42 | Wrong outcome |
| Modelling the spread of COVID-19 with new fractal-fractional operators: Can the lockdown save mankind before vaccination? | Atangana, A. | 2020 | Chaos Solitons & Fractals | 136 |  | 109860 | Wrong intervention |
| Heterogeneous social interactions and the COVID-19 lockdown outcome in a multi-group SEIR model | Augeraud, E.; Banerjee, M.; Dhersin, J. S.; et al | 2020 | Mathematical Modelling of Natural Phenomena | 15 |  | 18-Jan | Wrong intervention |
| Inequality of Fear and Self-Quarantine: Is There a Trade-off between GDP and Public Health? | Aum, S; Lee, S. Y; Shin, Y | 2020 |  |  |  |  | Wrong outcome |
| COVID-19 Doesn't Need Lockdowns to Destroy Jobs: The Effect of Local Outbreaks in Korea | Aum, S; Lee, S. Y; Shin, Y | 2020 |  |  |  |  | Wrong intervention |
| When Face-to-Face Interactions Become an Occupational Hazard : Jobs in the Time of COVID-19 | Avdiu, B; Nayyar, G | 2020 |  |  |  |  | Wrong study design |
| Utilizing adaptive AI-based information systems to analyze the effectiveness of policy measures in the fight of COVID-19 | Baier, L.; Schaeffer, J.; Kahl, N. | 2021 | International Conference on Information Systems, ICIS 2020 - Making Digital Inclusive: Blending the Local and the Global | | | | Wrong study design |
| CoVidAffect, real-time monitoring of mood variations following the COVID-19 outbreak in Spain | Bailon, C.; Goicoechea, C.; Banos, O.; et al | 2020 | Scientific Data | 7 | 1 | 365 | Wrong outcome |
| Nonrelocatable occupations at increased risk during pandemics: United States, 2018 | Baker, M. G. |  |  | 110 | 8 | 1126-1132 | Wrong outcome |
| An institutionally racist lockdown policy | Bandyopadhyay, S. | 2020 | The Lancet | 396 | 10265 | 1802 | Wrong study design |
| "Pandemonium of the pandemic": Impact of COVID-19 in India, focus on mental health | Banerjee, D.; Bhattacharya, P. | 2020 | Psychological Trauma:Theory, Pesearch, Practice and Policy | 3 |  | 3 | Wrong study design |
| U.S. county level analysis to determine If social distancing slowed the spread of COVID-19 | Banerjee, T.; Nayak, A. | 2020 | Pan American Journal of Public Health | 44 |  | e90 | Wrong intervention |
| The Mental Health Effects of the First Two Months of Lockdown during the COVID-19 Pandemic in the UK | Banks, James; Xu, Xiaowei | 2020 | Fiscal Studies | 41 | 3 | 685-708 | Wrong study design |
| Reopening Scenarios | Baqaee, D; Farhi, E; Mina, M. J.; Stock, J. H. | 2020 |  |  |  |  | Wrong study design |
| Far from the eyes, close to the heart: Psychological Impact of COVID-19 in a Sample of Italian foreign workers | Barbato, M.; Thomas, J. | 2020 | Psychiatry Research | 290 (no pagination) | 113113 |  | Wrong study design |
| COVID-19 Is Also a Reallocation Shock | Barrero, J. M; Bloom, N; Davis, S. J. | 2020 |  |  |  |  | Wrong outcome |
| Estimating the costs and benefits of mandated business closures in a pandemic | Barrot, Jean-Noel; Grassi, B; Sauvagnat, J | 2020 |  |  |  |  | Wrong study design |
| Psychosocial Effects and Use of Communication Technologies during Home Confinement in the First Wave of the COVID-19 Pandemic in Italy and The Netherlands | Bastoni, S.; Wrede, C.; Ammar, A.; et al | 2021 | International Journal of Environmental Research & Public Health [Electronic Resource] | 18 | 5 | 5 | Wrong study design |
| A Comprehensive Public Health Evaluation of Lockdown as a Non-pharmaceutical Intervention on COVID-19 Spread in India: National Trends Masking State Level Variations | Basu, D.; Salvatore, M.; Ray, D.; et al | 2020 | MedRxiv : the Preprint Server for Health Sciences | 14 |  | 14 | Wrong study design |
| COVID Social Distancing and the Poor: An Analysis of the Evidence for England | Basu, P.; Bell, C.; Edwards, T. H. | 2021 | B.E. Journal of Macroeconomics | | |  | Wrong intervention |
| Going by the numbers: Learning and modeling COVID-19 disease dynamics | Basu, S.; Campbell, R. H. | 2020 | Chaos, Solitons and Fractals | 138 |  |  | Wrong intervention |
| The Coronavirus Stimulus Package: How large is the transfer multiplier? | Bayer, C; Born, B; Luetticke, R; Muller, G | 2020 |  |  |  |  | Wrong intervention |
| Impact of school closures for COVID-19 on the US health-care workforce and net mortality: a modelling study | Bayham, J.; Fenichel, E. P. | 2020 | The lancet. Public Health | 5 | 5 | e271-e278 | Wrong setting |
| Covid-19 health crisis and lockdown associated with high level of sleep complaints and hypnotic uptake at the population level | Beck, F.; Leger, D.; Fressard, L.; Peretti-Watel, P.; Verger, P.; Coconel, G | 2020 | Journal of Sleep Research | | | e13119 | Wrong outcome |
| Covid-19, Family Stress and Domestic Violence: Remote Work, Isolation and Bargaining Power | Beland, Louis-Philippe; Brodeur, A; Haddad, J; Mikola, D | 2020 |  |  |  | 38 pages | Wrong intervention |
| Teleworking in the context of the Covid-19 crisis | Belzunegui-Eraso, A.; Erro-Garcia, A. | 2020 | Sustainability (Switzerland) | 12 | 9 |  | Wrong study design |
| Inverse correlation between average monthly high temperatures and COVID-19-related death rates in different geographical areas | Benedetti, F.; Pachetti, M.; Marini, B.; Ippodrino, R.; Gallo, R. C.; Ciccozzi, M.; Zella, D. | 2020 | Journal of Translational Medicine | 18 | 1 | 251 | Wrong intervention |
| Stay-at-home orders due to the COVID-19 pandemic are associated with elevated depression and anxiety in younger, but not older adults: results from a nationwide community sample of adults from Germany | Benke, C.; Autenrieth, L. K.; Asselmann, E.; Pane-Farre, C. A. | 2020 | Psychological Medicine | |  | 2-Jan | Wrong study design |
| All things equal? Heterogeneity in policy effectiveness against COVID-19 spread in chile | Bennett, M. | 2021 | World Development | 137 |  | 105208 | Wrong study design |
| Not the Last Word: How Necessary COVID-19 Lockdowns Can Go Too Far | Bernstein, J. | 2020 | Clinical Orthopaedics & Related Research | 478 | 8 | 1719-1724 | Wrong study design |
| Preliminary reflections of CAMHS in COVID-19 lockdown | Bertz, S.; Olatoye, O.; O'Hanrahan, S.; Zaidi, N. | 2020 | Irish Journal of Psychological Medicine | | | 2-Jan | Wrong study design |
| A Rational-Choice Model of COVID-19 Transmission with Endogenous Quarantining and Two-Sided Prevention | Bhattacharya, J; Chakraborty, S; Yu, X | 2021 | Journal of Mathematical Economics | 93 |  |  | Wrong study design |
| Understanding COVID-19 transmission through Bayesian probabilistic modeling and GIS-based Voronoi approach: a policy perspective | Bherwani, H.; Anjum, S.; Kumar, S.; et al | 2020 | Environment, Development and Sustainability | | | | Wrong outcome |
| Valuation of air pollution externalities: comparative assessment of economic damage and emission reduction under COVID-19 lockdown | Bherwani, H.; Nair, M.; Musugu, K.; et al | 2020 | Air Quality, Atmosphere and Health | 13 | 6 | 683-694 | Wrong outcome |
| COVID-19: Persistence, precautions, diagnosis and challenges | Bhusare, B. P.; Zambare, V. P.; Naik, A. A. | 2020 | Journal of Pure and Applied Microbiology | 14 | 1 | 823-829 | Wrong study design |
| Accumulation of economic hardship and health during the COVID-19 pandemic: Social causation or selection? | Bierman, A; Upenieks, L; Glavin, P; Schieman, S | 2021 | Social Science & Medicine Vol 275 2021, ArtID 113774 | 275 |  |  | Wrong intervention |
| Social network-based distancing strategies to flatten the COVID-19 curve in a post-lockdown world | Block, P.; Hoffman, M.; Raabe, I. J.; et al | 2020 | Nature Human Behaviour | 4 | 6 | 588-596 | Wrong intervention |
| Predicting the spread of COVID-19 in Italy using machine learning: Do socio-economic factors matter? | Bloise, F.; Tancioni, M. | 2021 | Structural Change and Economic Dynamics | 56 |  | 310-329 | Wrong study design |
| Self-Isolation Compliance In The COVID-19 Era Influenced By Compensation: Findings From A Recent Survey In Israel | Bodas, M.; Peleg, K. | 2020 | Health Affairs | 39 | 6 | 936-941 | Wrong intervention |
| Social Distancing and Supply Disruptions in a Pandemic | Bodenstein, M; Corsetti, G; Guerrieri, L | 2020 |  |  |  | 52 p. | Wrong outcome |
| Psychosocial and Socio-Economic Crisis in Bangladesh Due to COVID-19 Pandemic: A Perception-Based Assessment | Bodrud-Doza, M.; Shammi, M.; Bahlman, L.; Islam, Armt; Rahman, M. M. | 2020 | Frontiers in Public Health | 8 |  | 341 | Wrong comparator |
| Predicted Effects of Stopping COVID-19 Lockdown on Italian Hospital Demand | Bollon, J.; Paganini, M.; Nava, C. R.; et al | 2020 | Disaster Medicine & Public Health Preparedness | | | 5-Jan | Wrong study design |
| Economic and social consequences of human mobility restrictions under COVID-19 | Bonaccorsi, G.; Pierri, F.; Cinelli, M.; et al | 2020 | Proceedings of the National Academy of Sciences of the United States of America | 117 | 27 | 15530-15535 | Wrong outcome |
| Global Supply Chains in the Pandemic | Bonadio, B; Huo, Z; Levchenko, A. A.; Pandalai-Nayar, N | 2020 |  |  |  |  | Wrong outcome |
| Effects of Coronavirus Disease (COVID-19) Related Contact Restrictions in Germany, March to May 2020, on the Mobility and Relation to Infection Patterns | Bonisch, S.; Wegscheider, K.; Krause, L.; et al | 2020 | Frontiers in Public Health | 8 |  | 568287 | Wrong outcome |
| After Less Than 2 Months, the Simulations That Drove the World to Strict Lockdown Appear to be Wrong, the Same of the Policies They Generated | Boretti, A. | 2020 | Health Services Research & Managerial Epidemiology | 7 |  | 2.33E+15 | Wrong study design |
| Business Closures, Stay-at-Home Restrictions, and COVID-19 Testing Outcomes in New York City | Borjas, G. J. | 2020 | Preventing Chronic Disease | 17 |  | E109 | Wrong outcome |
| The Adverse Effect of the COVID-19 Labor Market Shock on Immigrant Employment | Borjas, G. J.; Cassidy, H | 2020 |  |  |  |  | Wrong intervention |
| Do lockdowns work? A counterfactual for Sweden | Born, B; Dietrich, A; Muller, G | 2020 |  |  |  |  | Wrong study design |
| A hybrid multi-scale model of COVID-19 transmission dynamics to assess the potential of non-pharmaceutical interventions | Bouchnita, A.; Jebrane, A. | 2020 | Chaos, Solitons and Fractals | 138 |  |  | Wrong study design |
| Africa needs local solutions to face the COVID-19 pandemic | Boum, Y.; Bebell, L. M.; Bisseck, A. C. Z. K. | 2021 | The Lancet | 397 | 10281 | 1238-1240 | Wrong study design |
| Does lockdown work? A spatial analysis of the spread and concentration of Covid-19 in Italy | Bourdin, S.; Jeanne, L.; Nadou, F.; Noiret, G. | 2021 | Regional Studies | |  |  | Wrong intervention |
| Working from home in the time of COVID-19: how to best preserve occupational health? | Bouziri, H.; Smith, D. R. M.; Descatha, A.; Dab, W.; Jean, K. | 2020 | Occupational & Environmental Medicine | 77 | 7 | 509-510 | Wrong study design |
| Spring in London with Covid-19: a personal view | Brahams, D. | 2020 | Medico-Legal Journal | 88 | 2 | 57-64 | Wrong study design |
| A literature review of the economics of COVID-19 | Brodeur, A.; Gray, D.; Islam, A.; Bhuiyan, S. | 2021 | Journal of Economic Surveys | | |  | Wrong study design |
| COVID-19, Lockdowns and Well-being: Evidence from Google Trends | Brodeur, A; Clark, A. E.; Fleche, S; Powdthavee, N | 2020 |  |  |  |  | Wrong outcome |
| Life in lockdown: a telephone survey to investigate the impact of COVID-19 lockdown measures on the lives of older people (>=75 years) | Brown, L.; Mossabir, R.; Harrison, N.; Brundle, C.; Smith, J.; Clegg, A. | 2021 | Age & Ageing | 50 | 2 | 341-346 | Wrong population |
| Coronavirus and health disparities in construction | Brown, S.; Brooks, R. D.; Dong, X. S. |  |  |  |  | 7-Jan | Wrong intervention |
| The Cost of Staying Open: Voluntary Social Distancing and Lockdowns in the US | Brzezinski, A; Van Dijcke, D; Kecht, V | 2020 |  |  |  |  | Wrong outcome |
| Severe Mental Health Symptoms during COVID-19: A Comparison of the United Kingdom and Austria | Budimir, S.; Pieh, C.; Dale, R.; Probst, T. | 2021 | Healthcare | 9 | 2 | 9 | Wrong comparator |
| Social consequences of COVID-19 in a low resource setting in Sierra Leone, West Africa | Buonsenso, D.; Cinicola, B.; Raffaelli, F.; Sollena, P.; Iodice, F. | 2020 | International Journal of Infectious Diseases | 97 |  | 23-26 | Wrong comparator |
| Suicide risk during the lockdown due to coronavirus disease (COVID-19) in Colombia | Caballero-Dominguez, C. C.; Jimenez-Villamizar, M. P.; Campo-Arias, A. | 2020 | Death Studies | |  | 6-Jan | Wrong comparator |
| Size and timescale of epidemics in the SIR framework | Cadoni, M.; Gaeta, G. | 2020 | Physica D: Nonlinear Phenomena | 411 |  |  | Wrong intervention |
| COVID-19 and Emerging Markets: An Epidemiological Multi-Sector Model for a Small Open Economy with an Application to Turkey | Cakmakli, C; Demiralp, S; KalemlOzcan, S; Yesiltas, S; Yildirim, M. A. | 2020 |  |  |  |  | Wrong study design |
| International HRM insights for navigating the COVID-19 pandemic: Implications for future research and practice | Caligiuri, P.; De Cieri, H.; Minbaeva, D.; Verbeke, A.; Zimmermann, A. | 2020 | Journal of International Business Studies | 51 | 5 | 697-713 | Wrong study design |
| Investigating the effects of COVID-19 on global male sex work populations: a longitudinal study of digital data | Callander, D.; Meunier, E.; DeVeau, R.; et al | 2020 | Sexually Transmitted Infections | 26 |  | 26 | Wrong outcome |
| COVID-19 and Sick Leave: An Analysis of the Ibermutua Cohort of Over 1,651,305 Spanish Workers in the First Trimester of 2020 | Calvo-Bonacho, E.; Catalina-Romero, C.; Fernandez-Labandera, C.; et al | 2020 | Frontiers in Public Health | 8 |  | 580546 | Wrong intervention |
| A Simulation of a COVID-19 Epidemic Based on a Deterministic SEIR Model | Carcione, J. M.; Santos, J. E.; Bagaini, C.; Ba, J. | 2020 | Frontiers in Public Health | 8 |  | 230 | Wrong intervention |
| Effect of national and local lockdowns on the control of COVID-19 pandemic: a rapid review | Caristia, S.; Ferranti, M.; Skrami, E.; et al | 2020 | Epidemiologia e Prevenzione | 44 | 5-6 Suppl 2 | 60-68 | Wrong study design |
| Tracking the COVID-19 Crisis with High-Resolution Transaction Data | Carvalho, V; Garcia, J. R.; Hansen, S; et al | 2020 |  |  |  |  | Wrong outcome |
| Protecting Lives and Livelihoods with Early and Tight Lockdowns | Caselli, F.; Grigoli, F.; Sandri, D. | 2021 | B.E. Journal of Macroeconomics | | |  | Wrong outcome |
| Considerations and best practices for elite football officials return to play after COVID-19 confinement | Castagna, C.; Bizzini, M.; Leguizamon, A. P.; et al | 2020 | Managing Sport and Leisure | | | 8-Jan | Wrong study design |
| Technology-enhanced auditing in voluntary sustainability standards: The impact of COVID-19 | Castka, P.; Searcy, C.; Fischer, S. | 2020 | Sustainability (Switzerland) | 12 | 11 |  | Wrong outcome |
| Changes in sleep pattern, sense of time and digital media use during COVID-19 lockdown in Italy | Cellini, N.; Canale, N.; Mioni, G.; Costa, S. | 2020 | Journal of Sleep Research | | | e13074 | Wrong outcome |
| Changes in sleep timing and subjective sleep quality during the COVID-19 lockdown in Italy and Belgium: age, gender and working status as modulating factors | Cellini, N.; Conte, F.; De Rosa, O.; et al | 2021 | Sleep Medicine | 77 |  | 112-119 | Wrong outcome |
| Covid-19 Outbreak In Italy: Are We Ready for the Psychosocial and the Economic Crisis? Baseline Findings From the PsyCovid Study | Cerami, C.; Santi, G. C.; Galandra, C.; et al | 2020 | Frontiers in psychiatry Frontiers Research Foundation | 11 |  | 556 | Wrong intervention |
| Systematic rapid "living" review on rehabilitation needs due to COVID-19: update to March 31st, 2020 | Ceravolo, M. G.; de Sire, A.; Andrenelli, E.; Negrini, F.; Negrini, S. | 2020 | European journal of physical & rehabilitation medicine. | 56 | 3 | 347-353 | Wrong setting |
| A nation-wide survey on emotional and psychological impacts of COVID-19 social distancing | Cerbara, L.; Ciancimino, G.; Crescimbene, M.; et al | 2020 | European Review for Medical & Pharmacological Sciences | 24 | 12 | 7155-7163 | Wrong comparator |
| A Generic Approach for Allocating Movement Permits During/Outside Curfew Period during COVID-19 | Chaaban, Y. | 2021 | International Journal of Advanced Computer Science and Applications | 12 | 3 | 192-200 | Wrong outcome |
| Psychological impact of COVID-19 pandemic on general population in West Bengal: A cross-sectional study | Chakraborty, K.; Chatterjee, M. | 2020 | Indian Journal of Psychiatry | 62 | 3 | 266-272 | Wrong comparator |
| The mental health impact of COVID-19 and lockdown-related stressors among adults in the UK | Chandola, T.; Kumari, M.; Booker, C. L.; Benzeval, M. | 2020 | Psychological Medicine | |  | 10-Jan | Wrong comparator |
| Statistical Explorations and Univariate Timeseries Analysis on COVID-19 Datasets to Understand the Trend of Disease Spreading and Death | Chatterjee, A.; Gerdes, M. W.; Martinez, S. G. | 2020 | Sensors | 20 | 11 | 29 | Wrong intervention |
| Healthcare impact of COVID-19 epidemic in India: A stochastic mathematical model | Chatterjee, K.; Chatterjee, K.; Kumar, A.; Shankar, S. | 2020 | Medical Journal Armed Forces India | 2 |  | 2 | Wrong study design |
| Community preparedness for COVID-19 and frontline health workers in Chhattisgarh | Chatterjee, P. K. | 2020 | Indian Journal of Public Health | 64 | Supplement | S102-S104 | Wrong study design |
| Studying the progress of COVID-19 outbreak in India using SIRD model | Chatterjee, S.; Sarkar, A.; Chatterjee, S.; Karmakar, M.; Paul, R. | 2020 | Indian Journal of Physics | |  |  | Wrong study design |
| A Qualitative Investigation of the Impact of COVID-19 on Macauâ€™s Gaming Industry | Chau, K. Y.; Luo, J. M.; Duan, X. | 2021 | Journal of Quality Assurance in Hospitality and Tourism | | | | Wrong outcome |
| A country level analysis measuring the impact of government actions, country preparedness and socioeconomic factors on COVID-19 mortality and related health outcomes | Chaudhry, R.; Dranitsaris, G.; Mubashir, T.; Bartoszko, J.; Riazi, S. | 2020 | EClinicalMedicine | 25 |  | 100464 | Wrong comparator |
| Epidemiological Trends of Coronavirus Disease 2019 in China | Chen, B.; Zhong, H.; Ni, Y.; Liu, L.; Zhong, J.; Su, X. | 2020 | Frontiers in Medicine | 7 |  | 259 | Wrong comparator |
| Distribution of the COVID-19 epidemic and correlation with population emigration from Wuhan, China | Chen, Z. L.; Zhang, Q.; Lu, Y.; et al | 2020 | Chinese Medical Journal | 133 | 9 | 1044-1050 | Wrong comparator |
| The role of community-wide wearing of face mask for control of coronavirus disease 2019 (COVID-19) epidemic due to SARS-CoV-2 | Cheng, V. C.; Wong, S. C.; Chuang, V. W.; et al | 2020 | Journal of Infection | 81 | 1 | 107-114 | Wrong intervention |
| Causal impact of masks, policies, behavior on early covid-19 pandemic in the U.S | Chernozhukov, V.; Kasahara, H.; Schrimpf, P. | 2021 | Journal of Econometrics | 220 | 1 | 23-62 | Wrong study design |
| Back to basics: understanding the numbers behind COVID-19 | Chiba, M. D. | 2021 | Emerald Emerging Markets Case Studies | 11 | 1 | 15-Jan | Wrong study design |
| COVID-19 pandemic: health impact of staying at home, social distancing and 'lockdown' measures-a systematic review of systematic reviews | Chiesa, V.; Antony, G.; Wismar, M.; Rechel, B. | 2021 | Journal of Public Health | 13 |  | 13 | Wrong study design |
| Commentary on COVID-19 in Zimbabwe | Chigevenga, Rosemary | 2020 | Psychological Trauma: Theory, Research, Practice, and Policy | 12 | 5 | 562-564 | Wrong study design |
| Frequency of routine testing for SARS-CoV-2 to reduce transmission among workers | Chin, E. T.; Lo, N. C.; Huynh, B. Q.; Murrill, M.; Basu, S. | 2020 | MedRxiv : the Preprint Server for Health Sciences | 6 |  | 6 | Wrong intervention |
| COVID-19 virus outbreak forecasting of registered and recovered cases after sixty day lockdown in Italy: A data driven model approach | Chintalapudi, N.; Battineni, G.; Amenta, F. | 2020 | Journal of Microbiology, Immunology & Infection | 53 | 3 | 396-403 | Wrong study design |
| Long-term strategies to control COVID-19 in low and middle-income countries: an options overview of community-based, non-pharmacological interventions | Chowdhury, R.; Luhar, S.; Khan, N.; Choudhury, S. R.; Matin, I.; Franco, O. H. | 2020 | European Journal of Epidemiology | 13 |  | 13 | Wrong study design |
| 'Standing together“ at a distance': Documenting changes in mental-health indicators in Denmark during the COVID-19 pandemic | Clotworthy, A; Dissing, A. S; Nguyen, T; et al | 2021 | Scandinavian Journal of Public Health | 49 | 1 | 79-87 | Wrong outcome |
| Structural barriers to adhering to health behaviours in the context of the COVID-19 crisis: Considerations for low- and middle-income countries | Coetzee, B. J.; Kagee, A. | 2020 | Global Public Health | 15 | 8 | 1093-1102 | Wrong study design |
| A case study of the Westchester County New York's jail response to COVID-19: Controlling COVID while balancing service needs for the incarcerated-A national model for jails | Collica-Cox, K; Molina, L | 2020 | Victims & Offenders | 15 | 8-Jul | 1305-1316 | Wrong setting |
| Compensation effect between deaths from Covid-19 and crashes: The Italian case | Colonna, P.; Intini, P. | 2020 | Transportation Research Interdisciplinary Perspectives | 6 |  |  | Wrong outcome |
| Official statistics in the search for solutions for living with COVID-19 and its consequences | Cook, L.; Gray, A. | 2020 | Statistical Journal of the IAOS | 36 | 2 | 253-278 | Wrong study design |
| The Impacts of COVID-19 on Minority Unemployment: First Evidence from April 2020 CPS Microdata | Couch, K. A.; Fairlie, R. W.; Xu, H | 2020 |  |  |  |  | Wrong intervention |
| Estimating actual COVID-19 case numbers using cumulative death count-A method of measuring effectiveness of lockdown of non-essential activities: a South African case study | Cox, L.; Yah, C. S. | 2020 | The Pan African medical journal | 35 | Suppl 2 | 97 | Wrong study design |
| Coronavirus, domestic labour and care: Gendered roles locked down | Craig, L. | 2020 | Journal of Sociology | |  |  | Wrong study design |
| Dual-earner parent couplesâ€™ work and care during COVID-19 | Craig, L.; Churchill, B. | 2020 | Gender, Work and Organization | | |  | Wrong study design |
| Spreading of infections on random graphs: A percolation-type model for COVID-19 | Croccolo, F.; Roman, H. E. | 2020 | Chaos, Solitons and Fractals | 139 |  |  | Wrong study design |
| Evaluating approaches to designing effective Co-Created hand-hygiene interventions for children in India, Sierra Leone and the UK | Crosby, S; Younie, S; Williamson, I; Laird, K | 2020 | PLoS ONE Vol 15(9), 2020, ArtID e0239234 | 15 | 9 |  | Wrong population |
| Quantifying The Relationship Between Lockdowns, Mobility, and Effective Reproduction Number (Rt) During The COVID-19 Pandemic in The Greater Toronto Area | Dainton, C.; Hay, A. | 2021 | Research Square | 8 |  | 8 | Wrong intervention |
| Early Impact of the COVID-19 Outbreak on Sleep in a Large Spanish Sample | Dal Santo, F.; Gonzalez-Blanco, L.; Rodriguez-Revuelta, J.; et al | 2021 | Behavioral Sleep Medicine | | | 16-Jan | Wrong outcome |
| Psychological distress and adaptation to the COVID-19 crisis in the United States | Daly, M.; Robinson, E. | 2021 | Journal of Psychiatric Research | 136 |  | 603-609 | Wrong outcome |
| Depression reported by US adults in 2017-2018 and March and April 2020 | Daly, M.; Sutin, A. R.; Robinson, E. | 2021 | Journal of Affective Disorders | 278 |  | 131-135 | Wrong comparator |
| Economic interventions to ameliorate the impact of COVID-19 on the economy and health: an international comparison | Danielli, S.; Patria, R.; Donnelly, P.; Ashrafian, H.; Darzi, A. | 2020 | Journal of Public Health | 13 |  | 13 | Wrong study design |
| Differences in rapid increases in county-level COVID-19 incidence by implementation of statewide closures and mask mandates - United States, June 1-September 30, 2020 | Dasgupta, S.; Kassem, A. M.; Sunshine, G.; et al | 2021 | Annals of Epidemiology | 57 |  | 46-53 | Wrong comparator |
| Effects of non-pharmaceutical interventions on COVID-19 cases, deaths, and demand for hospital services in the UK: a modelling study | Davies, N. G.; Kucharski, A. J.; Eggo, R. M.; et al | 2020 | The lancet. Public Health | 5 | 7 | e375-e385 | Wrong study design |
| COVID-19: Psychological flexibility, coping, mental health, and wellbeing in the UK during the pandemic | Dawson, D. L.; Golijani-Moghaddam, N. | 2020 | Journal of Contextual Behavioral Science | 17 |  | 126-134 | Wrong outcome |
| Covid-19: European officials warn that exiting lockdown will be "very long" and will require cooperation | Day, M. | 2020 | BMJ | 369 |  | m1549 | Wrong study design |
| How COVID-19 and the Dutch â€˜intelligent lockdownâ€™ change activities, work and travel behaviour: Evidence from longitudinal data in the Netherlands | de Haas, M.; Faber, R.; Hamersma, M. | 2020 | Transportation Research Interdisciplinary Perspectives | 6 |  |  | Wrong outcome |
| Covid-19 Mortality: A Matter of Vulnerability Among Nations Facing Limited Margins of Adaptation | De Larochelambert, Q.; Marc, A.; Antero, J.; Le Bourg, E.; Toussaint, J. F. | 2020 | Frontiers in Public Health | 8 |  | 604339 | Wrong intervention |
| Positive impact of lockdown on COVID-19 outbreak in Thailand | Dechsupa, S.; Assawakosri, S.; Phakham, S.; Honsawek, S. | 2020 | Travel Medicine & Infectious Disease | | | 101802 | Wrong study design |
| Behavioral changes before lockdown and decreased retail and recreation mobility during lockdown contributed most to controlling COVID-19 in Western countries | Deforche, K.; Vercauteren, J.; Muller, V.; Vandamme, A. M. | 2021 | BMC Public Health | 21 | 1 | 654 | Wrong study design |
| Covid-19 outbreak control, example of ministry of health of turkey | Demirbilek, Y.; Pehlivanturk, G.; Ozguler, Z. O.; Mese, E. A. L. P. | 2020 | Turkish Journal of Medical Sciences | 50 | SI-1 | 489-494 | Wrong study design |
| The Sooner, the Better : The Early Economic Impact of Non-Pharmaceutical Interventions during the COVID-19 Pandemic | Demirguc-Kunt, Asli; Lokshin, Michael M.; Torre, Ivan | 2020 |  |  |  |  | Wrong study design |
| Analysis of COVID-19 Transmission Sources in France by Self-Assessment Before and After the Partial Lockdown: Observational Study | Denis, F.; Septans, A. L.; Le Goff, F.; Jeanneau, S.; Lescure, F. X. | 2021 | Journal of Medical Internet Research | 23 | 5 | e26932 | Wrong outcome |
| An integrative Total Worker Health framework for keeping workers safe and healthy during the COVID-19 pandemic | Dennerlein, J. T.; Burke, L.; Sabbath, E. L.; et al | | | 52 | 5 | 689-696 | Wrong study design |
| The effects of COVID-19 induced lockdown measures on maritime settings of a coastal region | Depellegrin, D.; Bastianini, M.; Fadini, A.; Menegon, S. | 2020 | Science of the Total Environment | 740 |  | 140123 | Wrong outcome |
| Early Effects of Lockdown in India: Gender Gaps in Job Losses and Domestic Work | Deshpande, A. | 2020 | Indian Journal of Labour Economics | | | 4-Jan | Wrong study design |
| Change in psychological distress in response to changes in reduced mobility during the early 2020 COVID-19 pandemic: Evidence of modest effects from the U.S | Devaraj, S.; Patel, P. C. | 2021 | Social Science & Medicine | 270 |  | 113615 | Wrong outcome |
| Conceptualising COVID-19â€™s impacts on household food security | Devereux, S.; BÃ©nÃ©, C.; Hoddinott, J. | 2020 | Food Security | |  |  | Wrong study design |
| COVID-19 pandemic lockdown-induced altered sleep/wake circadian rhythm, health complaints and stress among traffic police personnel in India | Dey, A.; Majumdar, P.; Saha, A.; Sahu, S. | 2021 | Chronobiology International | 38 | 1 | 140-148 | Wrong outcome |
| Psychological distress associated with the COVID-19 lockdown: A two-wave network analysis | Di Blasi, M.; Gullo, S.; Mancinelli, E.; et al | 2021 | Journal of Affective Disorders | 284 |  | 18-26 | Wrong comparator |
| Impact of lockdown on COVID-19 epidemic in Ile-de-France and possible exit strategies | Di Domenico, L.; Pullano, G.; Sabbatini, C. E.; Boelle, P. Y.; Colizza, V. | 2020 | BMC Medicine | 18 | 1 | 240 | Wrong study design |
| Psychological Aspects and Eating Habits during COVID-19 Home Confinement: Results of EHLC-COVID-19 Italian Online Survey | Di Renzo, L.; Gualtieri, P.; Cinelli, G.; et al | 2020 | Nutrients | 12 | 7 | 19 | Wrong comparator |
| Is the COVID-19 lockdown nudging people to be more active: a big data analysis | Ding, D.; Del Pozo Cruz, B.; Green, M. A.; Bauman, A. E. | 2020 | British Journal of Sports Medicine | 30 |  | 30 | Wrong outcome |
| Article mental health among adults during the covid-19 pandemic lockdown: A cross-sectional multi-country comparison | Ding, K.; Yang, J.; Chin, M. K.; et al | 2021 | International Journal of Environmental Research and Public Health | 18 | 5 | 16-Jan | Wrong comparator |
| An evaluation of COVID-19 in Italy: A data-driven modeling analysis | Ding, Y.; Gao, L. | 2020 | Infectious Disease Modelling | 5 |  | 495-501 | Wrong intervention |
| How Many Jobs Can be Done at Home? | Dingel, J; Neiman, B | 2020 |  |  |  |  | Wrong outcome |
| Epidemiological control measures and predicted number of infections for SARS-CoV-2 pandemic: case study Serbia march-april 2020 | Djurovic, I. | 2020 | Heliyon | 6 | 6 | e04238 | Wrong outcome |
| Privilege and burden of im-/mobility governance: On the reinforcement of inequalities during a pandemic lockdown | Dobusch, L; Kreissl, K | 2020 | Gender, Work and Organization | | | No Pagination Specified | Wrong study design |
| Anxiety and depression in Canada during the COVID-19 pandemic: A national survey | Dozois, D. J. | 2021 | Canadian Psychology/Psychologie canadienne | 62 | 1 | 136-142 | Wrong comparator |
| COVID-19 UK Lockdown Forecasts and R <sub>0</sub> | Dropkin, G. | 2020 | Frontiers in Public Health | 8 |  | 256 | Wrong study design |
| Aggregated COVID-19 suicide incidences in India: Fear of COVID-19 infection is the prominent causative factor | Dsouza, D. D.; Quadros, S.; Hyderabadwala, Z. J.; Mamun, M. A. | 2020 | Psychiatry Research | 290 |  | 113145 | Wrong comparator |
| COVID-19 cripples global restaurant and hospitality industry | Dube, K.; Nhamo, G.; Chikodzi, D. | 2020 | Current Issues in Tourism | | |  | Wrong outcome |
| Psychosocial impact of COVID-19 | Dubey, S.; Biswas, P.; Ghosh, R.; et al | 2020 | Diabetes & Metabolic Syndrome | 14 | 5 | 779-788 | Wrong study design |
| Prediction of CoVid-19 infection, transmission and recovery rates: A new analysis and global societal comparisons | Duffey, R. B.; Zio, E. | 2020 | Safety Science | 129 |  | 104854 | Wrong intervention |
| Timing social distancing to avert unmanageable COVID-19 hospital surges | Duque, D.; Morton, D. P.; Singh, B.; Du, Z.; Pasco, R.; Meyers, L. A. | 2020 | Proceedings of the National Academy of Sciences of the United States of America | 29 |  | 29 | Wrong outcome |
| Asocial capital: Civic culture and social distancing during COVID-19 | Durante, R; Guiso, L; Gulino, G | 2020 |  |  |  |  | Wrong outcome |
| COVID-19 among workers in meat and poultry processing facilities - 19 States, April 2020 | Dyal, J. W.; Grant, M. P.; Broadwater, K.; et al | | | 69 | 18 | 557-561 | Wrong outcome |
| Covid-19: Africa records over 10 000 cases as lockdowns take hold | Dyer, O. | 2020 | BMJ | 369 |  | m1439 | Wrong study design |
| Covid-19: Trump fumes as adviser speaks out against lifting of US lockdowns | Dyer, O. | 2020 | BMJ | 369 |  | m1993 | Wrong study design |
| COVID-19: a perspective for lifting lockdown in Zimbabwe | Dzobo, M.; Chitungo, I.; Dzinamarira, T. | 2020 | The Pan African medical journal | 35 | Suppl 2 | 13 | Wrong study design |
| Before and after case reporting: A comparison of the knowledge, attitude and practices of the Jordanian population towards COVID-19 | Elayeh, E; Aleidi, S. M.; Ya'acoub, R; Haddadin, R. N. | 2020 | PLoS ONE Vol 15(10), 2020, ArtID e0240780 | 15 | 10 |  | Wrong outcome |
| Collective resilience in times of crisis: Lessons from the literature for socially effective responses to the pandemic | Elcheroth, G.; Drury, J. | 2020 | British Journal of Social Psychology | 59 | 3 | 703-713 | Wrong study design |
| Assessment of anxiety and depression symptoms in the Albanian general population during the outbreak of COVID-19 pandemic | Elezi, F; Tafani, G; Sotiri, E; Agaj, H; Kola, K | 2020 | Indian Journal of Psychiatry | 62 |  | S470-S475 | Wrong comparator |
| Mental health support in Jordan for the general population and for the refugees in the Zaatari camp during the period of COVID-19 lockdown | El-Khatib, Z; Al Nsour, M; Khader, Y S.; Abu Khudair, M | 2020 | Psychological Trauma: Theory, Research, Practice, and Policy | 12 | 5 | 511-514 | Wrong study design |
| One Month into the Reinforcement of Social Distancing due to the COVID-19 Outbreak: Subjective Health, Health Behaviors, and Loneliness among People with Chronic Medical Conditions | Elran-Barak, R.; Mozeikov, M. | 2020 | International Journal of Environmental Research & Public Health [Electronic Resource] | 17 | 15 | 27 | Wrong comparator |
| Impact of the COVID-19 Pandemic on Mental Health and Social Support among Adult Egyptians | El-Zoghby, Safaa M.; Soltan, Enayat M.; Salama, Hend M. | 2020 | Journal of Community Health | 45 | 4 | 689-695 | Wrong intervention |
| Associations of Government-Mandated Closures and Restrictions With Aggregate Mobility Trends and SARS-CoV-2 Infections in Nigeria | Erim, D. O.; Oke, G. A.; Adisa, A. O.; et al | 2021 | JAMA Network Open | 4 | 1 | e2032101 | Wrong study design |
| COVID-19 and "natural" experiments arising from physical distancing: a hypothetical case study from chronobiology | Erren, T. C.; Lewis, P.; Shaw, D. M. | 2020 | Chronobiology International | | | 3-Jan | Wrong study design |
| Estimating COVID-19 prevalence and infection control practices among US dentists | Estrich, C. G.; Mikkelsen, M.; Morrissey, R.; et al | 2020 | Journal of the American Dental Association | 151 | 11 | 815-824 | Wrong population |
| Supporting practice based evidence in the covid-19 crisis: Three researcher-practitioners' stories | Evans, C; Sabucedo, P; Paz, C | 2020 | Counselling Psychology Quarterly | | | No Pagination Specified | Wrong study design |
| Psychological distress, anxiety, family violence, suicidality, and wellbeing in New Zealand during the COVID-19 lockdown: A cross-sectional study | Every-Palmer, S.; Jenkins, M.; Gendall, P.; et al | 2020 | PLoS ONE [Electronic Resource] | 15 | 11 | e0241658 | Wrong study design |
| A lockdown index to assess the economic impact of the coronavirus | Faber, M.; Ghisletta, A.; Schmidheiny, K. | 2020 | Swiss Journal of Economics and Statistics | 156 | 1 | 11 | Wrong intervention |
| Business as Usual | Fabre Uribe, Susana | 2020 | Social Anthropology / Anthropologie Sociale | 28 | 2 | 265-266 | Wrong study design |
| Vexing, Veiled, and Inequitable: Social Distancing and the "Rights" Divide in the Age of COVID-19 | Fairchild, A.; Gostin, L.; Bayer, R. | 2020 | American Journal of Bioethics | 20 | 7 | 55-61 | Wrong study design |
|  | Fang, H; Wang, L; Yang, Y | 2020 |  |  |  | 39 pages | Wrong outcome |
| Human Mobility Restrictions and the Spread of the Novel Coronavirus (2019-nCoV) in China | Fang, H; Wang, L; Yang, Y | 2020 |  |  |  |  | Wrong outcome |
| Restarting the economy while saving lives under Covid-19 | Favero, C. A.; Ichino, A; Rustichini, A | 2020 |  |  |  |  | Wrong intervention |
| Quantifying SARS-CoV-2 transmission suggests epidemic control with digital contact tracing | Ferretti, L.; Wymant, C.; Kendall, M.; et al | 6491 | Science | 368 | 6491 | 8 | Wrong intervention |
| Cognitive and mental health changes and their vulnerability factors related to COVID-19 lockdown in Italy | Fiorenzato, E.; Zabberoni, S.; Costa, A.; Cona, G. | 2021 | PLoS ONE [Electronic Resource] | 16 | 1 | e0246204 | Wrong study design |
| Effects of the lockdown on the mental health of the general population during the COVID-19 pandemic in Italy: Results from the COMET collaborative network | Fiorillo, A.; Sampogna, G.; Giallonardo, V.; et al | 2020 | European Psychiatry: the Journal of the Association of European Psychiatrists | 63 | 1 | e87 | Wrong comparator |
| A guide to the coronavirus job retention scheme | Fisher, E.; Harwood, M. | 2020 | In Practice | 42 | 4 | 238-242 | Wrong study design |
| Estimating the effects of non-pharmaceutical interventions on COVID-19 in Europe | Flaxman, S.; Mishra, S.; Gandy, A.; et al | 2020 | Nature | 8 |  | 8 | Wrong study design |
| Fear and agony of the pandemic leading to stress and mental illness: An emerging crisis in the novel coronavirus (COVID-19) outbreak | Fofana, N. K.; Latif, F.; Sarfraz, S.; Bilal,; Bashir, M. F.; Komal, B. | 2020 | Psychiatry Research | 291 |  | 113230 | Wrong study design |
| A quantitative framework for exploring exit strategies from the COVID-19 lockdown | Fokas, A. S.; Cuevas-Maraver, J.; Kevrekidis, P. G. | 2020 | Chaos Solitons & Fractals | 140 |  | 110244 | Wrong study design |
| COVID-19 pandemic in the italian population: Validation of a post-traumatic stress disorder questionnaire and prevalence of PTSD symptomatology | Forte, G.; Favieri, F.; Tambelli, R.; Casagrande, M. | 2020 | International Journal of Environmental Research and Public Health | 17 | 11 | 16-Jan | Wrong comparator |
| Corona and Coffee on your commute: A spatial analysis of COVID-19 mortality and commuting flows in England in 2020 | Francetic, I.; Munford, L. | 2021 | European Journal of Public Health | 19 |  | 19 | Wrong intervention |
| Mental health services for mood disorder outpatients in Milan during COVID-19 outbreak: The experience of the health care providers at San Raffaele hospital | Franchini, L.; Ragone, N.; Seghi, F.; Barbini, B.; Colombo, C. | 2020 | Psychiatry Research | 292 |  | 113317 | Wrong population |
| Stranger months: how SARS-CoV-2, fear of contagion, and lockdown measures impacted attendance and clinical activity during February and March 2020 at an urban Emergency Department in Milan | Franchini, S.; Spessot, M.; Landoni, G.; et al | 2020 | Disaster Medicine & Public Health Preparedness | | | 23-Jan | Wrong outcome |
| Depression, dependence and prices of the COVID-19-Crisis | Frank, A; Fatke, B; Frank, W; Forstl, H; Holzle, P | 2020 | Brain, Behavior, and Immunity | 87 |  | 99 | Wrong study design |
| A simple tool for comparing benefits and 'costs' of COVID-19 exit strategies | Frank, J.; Williams, A. J. | 2020 | Public Health | 188 |  | 7-Apr | Wrong intervention |
| Database of epidemic trends and control measures during the first wave of COVID-19 in mainland China | Fu, H.; Wang, H.; Xi, X.; et al | 2021 | International Journal of Infectious Diseases | 102 |  | 463-471 | Wrong study design |
| Daily Contacts Under Quarantine amid Limited Spread of COVID-19 in Taiwan | Fu, Y. C.; Lee, H. W. | 2020 | International Journal of Sociology | | |  | Wrong intervention |
| Coping behaviors associated with decreased anxiety and depressive symptoms during the COVID-19 pandemic and lockdown | Fullana, M. A.; Hidalgo-Mazzei, D.; Vieta, E.; Radua, J. | 2020 | Journal of Affective Disorders | 275 |  | 80-81 | Wrong comparator |
| Preventing internal COVID-19 outbreaks within businesses and institutions: A methodology based on social networks analysis for supporting occupational health and safety services decision making | Gallardo, E. C.; de Arroyabe, J. C. F.; Arranz, N. | 2020 | Sustainability (Switzerland) | 12 | 11 |  | Wrong outcome |
| Immediate and delayed psychological effects of province-wide lockdown and personal quarantine during the COVID-19 outbreak in China | Gan, Y.; Ma, J.; Wu, J.; Chen, Y.; Zhu, H.; Hall, B. J. | 2020 | Psychological Medicine | |  | 12-Jan | Wrong intervention |
| COVID-19 Infection Pandemic: From the Frontline in Italy | Gastaldelli, A.; Gastaldelli, M.; Bastianoni, S. | 2020 | Journal of the American College of Nutrition | 39 | 8 | 677-684 | Wrong outcome |
| COVID-19 as an occupational disease? | George, R.; George, A. | 2020 | South African Medical Journal | 110 | 4 | 260 | Wrong study design |
| COVID-19 mortality rates in the European Union, Switzerland, and the UK: effect of timeliness, lockdown rigidity, and population density | Gerli, A. G.; Centanni, S.; Miozzo, M. R.; et al | 2020 | Minerva Medica | 2 |  | 2 | Wrong study design |
| Emerging Adults and COVID-19: The Role of Individualism-Collectivism on Perceived Risks and Psychological Maladjustment | Germani, A.; Buratta, L.; Delvecchio, E.; Mazzeschi, C. | 2020 | International Journal of Environmental Research & Public Health [Electronic Resource] | 17 | 10 | 17 | Wrong intervention |
| Coronavirus-related opportunities for promoting occupational health and safety | Gharibi, V.; Cousins, R.; Jahangiri, M. | 2020 | Shiraz E Medical Journal | 21 | 7 | 2-Jan | Wrong study design |
| Impact of complete lockdown on total infection and death rates: A hierarchical cluster analysis | Ghosal, S.; Bhattacharyya, R.; Majumder, M. | 2020 | Diabetes & Metabolic Syndrome | 14 | 4 | 707-711 | Wrong study design |
| The Italian value chain in the pandemic: the inputâ€“output impact of Covid-19 lockdown | Giammetti, R.; Papi, L.; Teobaldelli, D.; Ticchi, D. | 2020 | Journal of Industrial and Business Economics | | | | Wrong outcome |
| Implications of COVID-19: The Effect of Working From Home on Financial and Mental Well-Being in the UK | Giovanis, E.; Ozdamar, O. | 2021 | International Journal of Health Policy & Management | 21 |  | 21 | Wrong outcome |
| Community pharmacist's professional adaptation amid Covid-19 emergency: a national survey on Italian pharmacists | Giua, C.; Paoletti, G.; Minerba, L.; et al | 2021 | International Journal of Clinical Pharmacy | 15 |  | 15 | Wrong setting |
| A framework for identifying and mitigating the equity harms of COVID-19 policy interventions | Glover, R. E.; van Schalkwyk, M. C. I.; Akl, E. A.; et al | 2020 | Journal of Clinical Epidemiology | 128 |  | 35-48 | Wrong study design |
| A framework for identifying and mitigating the equity harms of COVID-19 policy interventions | Glover, R. E.; van Schalkwyk, M. C.; Akl, E. A.; et al | 2020 | Journal of Clinical Epidemiology | 8 |  | 8 | Wrong study design |
| Covid-19: It's too soon to lift lockdown | Godlee, F. | 2020 | The BMJ | 369 (no pagination) | m2202 |  | Wrong study design |
| Covid-19: A wake-up call | Godlee, F. | 2020 | The BMJ | 369 (no pagination) | m2021 |  | Wrong study design |
| COVID-19, India, lockdown and psychosocial challenges: What next? | Golechha, M. | 2020 | International Journal of Social Psychiatry | | | 2.08E+13 | Wrong study design |
| Life under lockdown: Notes on Covid-19 in Silicon Valley | GonzAlez, R. J.; Marlovits, J. | 2020 | Anthropology Today | 36 | 3 | 15-Nov | Wrong study design |
| On the Optimal 'Lockdown' during an Epidemic | Gonzalez-Eiras, Martin; Niepelt, Dirk | 2020 |  |  |  |  | Wrong outcome |
| Modelling SARS-COV2 Spread in London: Approaches to Lift the Lockdown | Gosce, L.; Phillips, P. A.; Spinola, P.; Gupta, D. R. K.; Abubakar, P. I. | 2020 | Journal of Infection | 81 | 2 | 260-265 | Wrong study design |
| Deterioration of mental health and insufficient Covid-19 information among disadvantaged immigrants in the greater Paris area | Gosselin, A.; Melchior, M.; Carillon, S.; et al | 2021 | Journal of Psychosomatic Research | 146 |  | 110504 | Wrong population |
| COVID-19 Pandemic and Economic Performances of the States in India | Goswami, Binoy; Mandal, Raju; Nath, Hiranya K. | 2021 | Economic Analysis and Policy | 69 |  | 461-479 | Wrong outcome |
| 100 Days of COVID-19 in India: Current and future trends | Gouda, S.; Naveen, G.; Kukanur, F. S. | 2020 | Journal of Pure and Applied Microbiology | 14 | Supplement 1 | 1043-1052 | Wrong intervention |
| Homeless persons with mental illness and COVID pandemic: Collective efforts from India | Gowda, G. S.; Chithra, N. K.; Moirangthem, S.; Kumar, C. N.; Math, S. B. | 2020 | Asian Journal of Psychiatry | 54 |  | 102268 | Wrong study design |
| Effects of the COVID-19 Emergency and National Lockdown on Italian Citizens' Economic Concerns, Government Trust, and Health Engagement: Evidence From a Two-Wave Panel Study | Graffigna, G.; Palamenghi, L.; Savarese, M.; Castellini, G.; Barello, S. | 2021 | Milbank Quarterly | 6 |  | 6 | Wrong outcome |
| Feel Better, Work Better: The COVID-19 Perspective | Graham, M. M.; Higginson, L.; Brindley, P. G.; Jetly, R. | 2020 | Canadian Journal of Cardiology | 36 | 6 | 789-791 | Wrong study design |
| Changes in young adults' alcohol and marijuana use, norms, and motives from before to during the COVID-19 pandemic | Graupensperger, Scott; Fleming, Charles B.; Jaffe, Anna E.; et al | 2021 | Journal of Adolescent Health | 68 | 4 | 658-665 | Wrong intervention |
| Behavioral strategies for reducing disease transmission in the workplace | Gravina, N.; Nastasi, J. A.; Sleiman, A. A.; Matey, N.; Simmons, D. E. | 2020 | Journal of Applied Behavior Analysis | 53 | 4 | 1935-1954 | Wrong study design |
| The Influence of the COVID-19 Pandemic on Mental Well-Being and Psychological Distress: Impact Upon a Single Country | Gray, N. S.; O'Connor, C.; Knowles, J.; et al | 2020 | Frontiers in psychiatry Frontiers Research Foundation | 11 |  | 594115 | Wrong outcome |
| Agriculture, transportation, and the COVID-19 crisis | Gray, R. S. | 2020 | Canadian Journal of Agricultural Economics | 68 | 2 | 239-243 | Wrong study design |
| Pandemic Recession: L or V-Shaped? | Gregory, V; Menzio, G; Wiczer, D | 2020 |  |  |  |  | Wrong intervention |
| A Tale of Three Countries: What is the Relationship Between COVID-19, Lockdown and Happiness? | Greyling, T.; Rossouw, S.; Adhikari, T. | 2021 | South African Journal of Economics | 89 | 1 | 25-43 | Wrong outcome |
| Increases in health-related workplace absenteeism among workers in essential critical infrastructure occupations during the COVID-19 pandemic - United States, March-April 2020 | Groenewold, M. R.; Burrer, S. L.; Ahmed, F.; Uzicanin, A.; Free, H.; Luckhaupt, S. E. | | | 69 | 27 | 853-858 | Wrong intervention |
| Cabin crew health and fitness-to-fly: Opportunities for re-evaluation amid COVID-19 | Grout, A.; Leggat, P. A. | 2021 | Travel Medicine & Infectious Disease | 40 |  | 101973 | Wrong study design |
| Effects of Covid-19 Lockdown on Mental Health and Sleep Disturbances in Italy | Gualano, M. R.; Lo Moro, G.; Voglino, G.; Bert, F.; Siliquini, R. | 2020 | International Journal of Environmental Research & Public Health [Electronic Resource] | 17 | 13 | 2 | Wrong comparator |
| They are Essential Workers Now, and Should Continue to Be: Social Workers and Home Health Care Workers during COVID-19 and Beyond | Guerrero, L. R.; Avgar, A. C.; Phillips, E.; Sterling, M. R. | 2020 | Journal of Gerontological Social Work | | | 3-Jan | Wrong study design |
| COVID-19 lockdown and penalty of joblessness on income and remittances: A study of inter-state migrant labourers from Assam, India | Guha, P.; Islam, B.; Hussain, M. A. | 2020 | Journal of Public Affairs | |  | e2470 | Wrong study design |
| Going through the lockdown: a longitudinal study on the psychological consequences of the coronavirus pandemic | Gullo, S.; Misici, I.; Teti, A.; Liuzzi, M.; Chiara, E. | 2020 | Research in Psychotherapy | 23 | 3 | 494 | Wrong comparator |
| Suicide risk and prevention during the COVID-19 pandemic | Gunnell, D; Appleby, L; Arensman, E; et al | 2020 | The Lancet Psychiatry | 7 | 6 | 468-471 | Wrong study design |
| A Mixed-method Study of Individual, Couple and Parental Functioning During the State-regulated COVID-19 Lockdown in Spain | Gunther-Bel, C.; Vilaregut, A.; Carratala, E.; Torras-Garat, S.; Perez-Testor, C. | 2020 | Family Process | 17 |  | 17 | Wrong comparator |
| Coping with COVID-19: Exposure to COVID-19 and Negative Impact on Livelihood Predict Elevated Mental Health Problems in Chinese Adults | Guo, J.; Feng, X. L.; Wang, X. H.; van, IJzendoorn M. H. | 2020 | International Journal of Environmental Research & Public Health [Electronic Resource] | 17 | 11 | 29 | Wrong comparator |
| Psychological impact of 'Lockdown' due to COVID-19 pandemic in Nepal: An online survey | Gupta, A. K.; Sahoo, S.; Mehra, A.; Grover, S. | 2020 | Asian Journal of Psychiatry | 54 |  | 102243 | Wrong comparator |
| Lockdown-the only solution to defeat COVID-19 | Gupta, A.; Singla, M.; Bhatia, H.; Sharma, V. | 2020 | International Journal Of Diabetes In Developing Countries | | | 2-Jan | Wrong study design |
| COVID-19 outbreak and Urban dynamics: regional variations in India | Gupta, D.; Biswas, D.; Kabiraj, P. | 2021 | GeoJournal |  |  | 19-Jan | Wrong intervention |
| Changes in sleep pattern and sleep quality during COVID-19 lockdown | Gupta, R.; Grover, S.; Basu, A.; et al | 2020 | Indian Journal of Psychiatry | 62 | 4 | 370-378 | Wrong outcome |
| Association of State-Issued Mask Mandates and Allowing On-Premises Restaurant Dining with County-Level COVID-19 Case and Death Growth Rates - United States, March 1-December 31, 2020 | Guy, G. P., Jr.; Lee, F. C.; Sunshine, G.; et al | 2021 | MMWR - Morbidity & Mortality Weekly Report | 70 | 10 | 350-354 | Wrong intervention |
| The impact of government measures and human mobility trend on COVID-19 related deaths in the UK | Hadjidemetriou, G. M.; Sasidharan, M.; Kouyialis, G.; Parlikad, A. K. | 2020 | Transportation Research Interdisciplinary Perspectives | 6 |  |  | Wrong study design |
| COVID-19 Mitigation With Appropriate Safety Measures in an Essential Workplace: Lessons for Opening Work Settings in the United States During COVID-19 | Haigh, K. Z.; Gandhi, M. | 2021 | Open Forum Infectious Diseases | 8 | 4 | ofab086 | Wrong outcome |
| Excess mortality during the COVID-19 pandemic in Israel, March-November 2020: when, where, and for whom? | Haklai, Z.; Aburbeh, M.; Goldberger, N.; Gordon, E. S. | 2021 | Israel Journal of Health Policy Research | 10 | 1 | 17 | Wrong intervention |
| How COVID-19 redefines the concept of sustainability | Hakovirta, M.; Denuwara, N. | 2020 | Sustainability (Switzerland) | 12 | 9 |  | Wrong study design |
| Effects of COVID-19 pandemic in daily life | Haleem, A.; Javaid, M.; Vaishya, R. | 2020 | Current Medicine Research and Practice | 10 | 2 | 78-79 | Wrong study design |
| COVID-19 Case and Contact Investigation in an Office Workspace | Hall, M. T.; Bui, H. Q.; Rowe, J.; Do, T. A. | 2020 | Military Medicine | 17 |  | 17 | Wrong intervention |
| Older Workers in the Time of COVID-19: The Senior Community Service Employment Program and Implications for Social Work | Halvorsen, C. J.; Yulikova, O. | 2020 | Journal of Gerontological Social Work | | | 12-Jan | Wrong study design |
| Assessment of preventive measures application against Covid-19 in the workplace | Hamadouche, M.; Allouche, S. | 2020 | Tunisie Medicale | 98 | 9-Aug | 625-632 | Wrong outcome |
| Disparities in COVID-19 Mortality by County Racial Composition and the Role of Spring Social Distancing Measures | Hamman, Mary K. | 2021 | Economics and Human Biology | 41 |  |  | Wrong intervention |
| Depression Following COVID-19 Lockdown in Severely, Moderately, and Mildly Impacted Areas in China | Han, X.; Chen, S.; Bi, K.; Yang, Z.; Sun, P. | 2021 | Frontiers in psychiatry Frontiers Research Foundation | 12 |  | 596872 | Wrong comparator |
| Biological security of the SARS-CoV-2 (COVID-19) infection in large workplaces outside the healthcare sector - an epidemiologist's point of view | Hanke, W.; Pietrzak, P. | 2021 | Medycyna Pracy | 72 | 1 | 89-97 | Wrong study design |
| Locked and lonely? A longitudinal assessment of loneliness before and during the COVID-19 pandemic in Norway | Hansen, T.; Nilsen, T. S.; Yu, B.; et al | 2021 | Scandinavian Journal of Public Health | | | 1.4E+15 | Wrong outcome |
| [Preventing Covid-19 after lockdown: For a rapid comeback to "life before"] | Hanslik, T.; Flahault, A. | 2020 | Revue de Medecine Interne | 41 | 6 | 358-359 | Language not English; |
| Do psychiatric patients experience more psychiatric symptoms during COVID-19 pandemic and lockdown? A case-control study with service and research implications for immunopsychiatry | Hao, F.; Tan, W.; Jiang, L.; et al | 2020 | Brain, Behavior, & Immunity | 87 |  | 100-106 | Wrong comparator |
| COVID-19, Bar Crowding, and the Wisconsin Supreme Court: A Non-linear Tale of Two Counties | Harris, Jeffrey E. | 2020 | Research in International Business and Finance | 54 |  |  | Wrong study design |
| Social distancing and the use of PPE by community pharmacy personnel: Does evidence support these measures? | Hasan, S. S.; Kow, C. S.; Zaidi, S. T. R. | 2020 | Research In Social & Administrative Pharmacy | 1 |  | 1 | Wrong study design |
| Jobs' Amenability to Working from Home : Evidence from Skills Surveys for 53 Countries | Hatayama, M; Viollaz, M; Winkler, H. J | 2020 |  |  |  |  | Wrong outcome |
| A US Population Health Survey on the Impact of COVID-19 Using the EQ-5D-5L | Hay, J. W.; Gong, C. L.; Jiao, X.; et al | 2021 | Journal of General Internal Medicine | 8 |  | 8 | Wrong intervention |
| The COVID-19 Pandemic: A Month of Bioethics in Finland | Hayry, M. | 2020 | Cambridge Quarterly of Healthcare Ethics | | | 9-Jan | Wrong study design |
| Changes in Body Weight, Physical Activity, and Lifestyle During the Semi-lockdown Period After the Outbreak of COVID-19 in China: An Online Survey | He, M.; Xian, Y.; Lv, X.; He, J.; Ren, Y. | 2020 | Disaster Medicine & Public Health Preparedness | | | 6-Jan | Wrong outcome |
| Commentary: Assessing the global impact of the COVID-19 pandemic on prison populations | Heard, Catherine | 2020 | Victims & Offenders | 15 | 8-Jul | 848-861 | Wrong study design |
| Adapt to a "new normal" during uncertain times | Hennessy, M. | 2020 | Drug Topics | 164 | 6 | 1 | Wrong study design |
| The first months of the COVID-19 pandemic in Spain | Henriquez, J.; Gonzalo-Almorox, E.; Garcia-Goni, M.; Paolucci, F. | 2020 | Health Policy & Technology | 9 | 4 | 560-574 | Wrong study design |
| Which jobs are done from home? Evidence from the American Time Use Survey | Hensvik, L; Le Barbanchon, T; Rathelot, R | 2020 |  |  |  |  | Wrong outcome |
| Effects of COVID-19 Lockdown on Physical Activity, Sedentary Behavior, and Satisfaction with Life in Qatar: A Preliminary Study | Hermassi, S.; Sellami, M.; Salman, A.; et al | 2021 | International Journal of Environmental Research & Public Health [Electronic Resource] | 18 | 6 | 17 | Wrong outcome |
| Protecting health during COVID-19 and beyond: A global examination of paid sick leave design in 193 countries | Heymann, J.; Raub, A.; Waisath, W.; et al | 2020 | Global Public Health | 15 | 7 | 925-934 | Wrong outcome |
| Novel fractional order SIDARTHE mathematical model of COVID-19 pandemic | Higazy, M. | 2020 | Chaos Solitons & Fractals | 138 |  | 110007 | Wrong intervention |
| Lab life - rebuild it better after coronavirus lockdowns ease | Hirschey, M. | 2020 | Nature | 582 | 7811 | 184 | Wrong study design |
| Education, health behavior, and working conditions during the pandemic: evidence from a German sample | Hoenig, K.; Wenz, S. E. | 2021 | European Societies | 23 | S1 | S275-S288 | Wrong outcome |
| A stochastic agent-based model of the SARS-CoV-2 epidemic in France | Hoertel, N.; Blachier, M.; Blanco, C.; et al | 2020 | Nature Medicine | 14 |  | 14 | Wrong study design |
| Lockdown exit strategies and risk of a second epidemic peak: a stochastic agent-based model of SARS-CoV-2 epidemic in France | Hoertel, N.; Blachier, M.; Blanco, C.; et al | 2020 | MedRxiv : the Preprint Server for Health Sciences | 5 |  | 5 | Wrong intervention |
| Effect of preventive measures in the containment of SARS-CoV-2 epidemic: a comparative study | Hoguane, A. M.; Priya, K. L.; Haddout, S.; Ljubenkov, I. | 2021 | Health Care for Women International | | | 16-Jan | Wrong study design |
| Effectiveness of Corona Lockdowns: Evidence for a Number of Countries | Homburg, S. | 2020 | Economists' Voice | |  |  | Wrong study design |
| Implications of Stochastic Transmission Rates for Managing Pandemic Risks | Hong, Harrison; Wang, Neng; Yang, Jinqiang | 2020 |  |  |  |  | Wrong intervention |
| Effect of COVID-19 Non-Pharmaceutical Interventions and the Implications for Human Rights | Hong, S. H.; Hwang, H.; Park, M. H. | 2020 | International Journal of Environmental Research & Public Health [Electronic Resource] | 18 | 1 | 30 | Wrong study design |
| Offline: COVID-19-bewilderment and candour | Horton, R. | 2020 | The Lancet | 395 | 10231 | 1178 | Wrong study design |
| Understanding epidemic data and statistics: A case study of COVID-19 | Hoseinpour Dehkordi, A.; Alizadeh, M.; Derakhshan, P.; Babazadeh, P.; Jahandideh, A. | 2020 | Journal of Medical Virology | 92 | 7 | 868-882 | Wrong intervention |
| Lockdown-type containment measures for COVID-19 prevention and control: a descriptive ecological study with data from South Africa, Germany, Brazil, Spain, United States, Italy and New Zealand, February - August 2020 | Houvessou, G. M.; Souza, T. P.; Silveira, M. F. D. | 2021 | Epidemiologia e Servicos de Saude | 30 | 1 | e2020513 | Wrong study design |
| The Outcome and Implications of Public Precautionary Measures in Taiwan-Declining Respiratory Disease Cases in the COVID-19 Pandemic | Hsieh, C. C.; Lin, C. H.; Wang, W. Y. C.; Pauleen, D. J.; Chen, J. V. | 2020 | International Journal of Environmental Research & Public Health [Electronic Resource] | 17 | 13 | 6 | Wrong intervention |
| Analysis of COVID-19 Cases and Public Measures in China | Hu, C. S. | 2020 | SN Comprehensive Clinical Medicine | | | 7-Jan | Wrong intervention |
| Intersecting ethnic and nativeâ€“migrant inequalities in the economic impact of the COVID-19 pandemic in the UK | Hu, Y. | 2020 | Research in Social Stratification and Mobility | 68 |  |  | Wrong study design |
| Measures Undertaken in China to Avoid COVID-19 Infection: Internet-Based, Cross-Sectional Survey Study | Huang, Y.; Wu, Q.; Wang, P.; et al | 2020 | Journal of Medical Internet Research | 22 | 5 | e18718 | Wrong intervention |
| Timing matters: the impact of response measures on COVID-19-related hospitalization and death rates in Germany and Switzerland | Huber, M.; Langen, H. | 2020 | Swiss Journal of Economics and Statistics | 156 | 1 | 10 | Wrong comparator |
| Covid-19: a comprehensive review of a formidable foe and the road ahead | Hussain, M. A.; Yadav, S.; Hadda, V.; et al | 2020 | Expert Review of Respiratory Medicine | | | 11-Jan | Wrong study design |
| A sharp increase in the number of COVID-19 cases and case fatality rates after lifting the lockdown in Kurdistan region of Iraq | Hussein, N. R.; Naqid, I. A.; Saleem, Z. S. M.; Almizori, L. A.; Musa, D. H.; Ibrahim, N. | 2020 | Annals of Medicine and Surgery | 57 |  | 140-142 | Wrong comparator |
| Emotional exhaustion among the South Korean workforce before and after COVID-19 | Hwang, H.; Hur, W. M.; Shin, Y. | 2020 | Psychology & Psychotherapy: Theory, Research & Practice | 27 |  | 27 | Wrong outcome |
| Covid-19: Cycle of 50 day lockdowns and 30 day relaxations could be effective, study finds | Iacobucci, G. | 2020 | BMJ | 369 |  | m2037 | Wrong study design |
| Lockdown, one, two, none, or smart. Modeling containing covid-19 infection. A conceptual model | Ibarra-Vega, D. | 2020 | Science of the Total Environment | 730 |  | 138917 | Wrong study design |
| Benchmarking SARS CoV-2 Infection in the Workplace to Support Continuity of Operations | Iddins, B. O.; Waugh, M. H.; Buck, B.; et al | 2021 | Journal of Occupational & Environmental Medicine | 19 |  | 19 | Wrong intervention |
| Effectiveness of the non-pharmaceutical public health interventions against COVID-19; a protocol of a systematic review and realist review | Iezadi, S.; Azami-Aghdash, S.; Ghiasi, A.; et al | 2020 | PLoS ONE [Electronic Resource] | 15 | 9 | e0239554 | Wrong study design |
| Analysing governmental response to the COVID-19 pandemic | Imtyaz, A.; Abid, Haleem; Javaid, M. | 2020 | Journal of Oral Biology & Craniofacial Research | 10 | 4 | 504-513 | Wrong intervention |
| Psychological Intervention and COVID-19: What We Know So Far and What We Can Do | Inchausti, F.; MacBeth, A.; Hasson-Ohayon, I.; Dimaggio, G. | 2020 | Journal of Contemporary Psychotherapy. | | | | Wrong study design |
| COVID-19 lockdown: implication for food security | Inegbedion, H. E. | 2020 | Journal of Agribusiness in Developing and Emerging Economies | | | | Wrong outcome |
| Coronavirus disease (COVID-19) pandemic: Furnishing experiences from India | Iqbal, N.; Dar, K. A. | 2020 | Psychological Trauma:Theory, Pesearch, Practice and Policy | 12 | S1 | S33-S34 | Wrong study design |
| Working from Home, Wages, and Regional Inequality in the Light of COVID-19 | Irlacher, Michael; Koch, Michael | 2020 |  |  |  |  | Wrong outcome |
| COVID-19 outbreak in post-soviet states: Modeling the best and worst possible scenarios | Issanov, A.; Amanbek, Y.; Abbay, A.; et al | 2020 | Electronic Journal of General Medicine | 17 | 6 | 12-Jan | Wrong intervention |
| Changes in mental well-being of adult Poles in the early period of the COVID-19 pandemic with reference to their occupational activity and remote work | Izdebski, Z. W.; Mazur, J. | 2021 | International Journal of Occupational Medicine & Environmental Health | 17 |  | 17 | Wrong outcome |
| Inequality in learning opportunities during Covid-19: Evidence from library takeout | JÃ¦ger, M. M.; BlaabÃ¦k, E. H. | 2020 | Research in Social Stratification and Mobility | 68 |  |  | Wrong population |
| Statewide COVID-19 Stay-at-Home Orders and Population Mobility in the United States | Jacobsen, G. D.; Jacobsen, K. H. | 2020 | World Medical and Health Policy. | | |  | Wrong outcome |
| Attendance at London workplaces after symptom onset: a retrospective cohort study of staff members with confirmed COVID-19 | Jain, V.; Waghorn, M.; Thorn-Heathcock, R.; Lamb, P.; Bell, A.; Addiman, S. | 2021 | Journal of Public Health | 12 |  | 12 | Wrong outcome |
| Analysis of COVID-19 burden, epidemiology and mitigation strategies in Muslim majority countries | Jardine, R.; Wright, J.; Samad, Z.; Bhutta, Z. A. | 2020 | Eastern Mediterranean Health Journal | 26 | 10 | 1173-1183 | Wrong intervention |
| Quantifying the impact of physical distance measures on the transmission of COVID-19 in the UK | Jarvis, C. I.; Van Zandvoort, K.; Gimma, A.; et al | 2020 | BMC Medicine | 18 | 1 | 124 | Wrong outcome |
| COVID-19 in New Zealand and the impact of the national response: a descriptive epidemiological study | Jefferies, S.; French, N.; Gilkison, C.; et al | 2020 | The lancet. Public Health | 5 | 11 | e612-e623 | Wrong study design |
| Lockdown contained the spread of 2019 novel coronavirus disease in Huangshi city, China: Early epidemiological findings | Ji, T.; Chen, H. L.; Xu, J.; Wu, L. N.; Li, J. J.; Chen, K.; Qin, G. | 2020 | Clinical Infectious Diseases | 7 |  | 7 | Wrong comparator |
| Influence of population mobility on the novel coronavirus disease (COVID-19) epidemic: based on panel data from Hubei, China | Jiang, J.; Luo, L. | 2020 | Global Health Research and Policy | 5 |  | 30 | Wrong outcome |
| Accounting for Global COVID-19 Diffusion Patterns, January-April 2020 | Jinjarak, Y; Ahmed, R; Nair-Desai, S; Xin, W; Aizenman, J | 2020 |  |  |  |  | Wrong intervention |
| Covid-19 in occupational settings: Lessons from 100 years ago and addressing the disease today | Joseph, B.; Kallumkal, M. G. | 2020 | Indian Journal of Community Health | 32 | 2 Special Issue | 255-260 | Wrong study design |
| Indian economy amid COVID-19 lockdown: A prespective | Joshi, A.; Bhaskar, P.; Gupta, P. K. | 2020 | Journal of Pure and Applied Microbiology | 14 |  | 957-961 | Wrong study design |
| European SMEs amidst the COVID-19 crisis: assessing impact and policy responses | Juergensen, J.; GuimÃ³n, J.; Narula, R. | 2020 | Journal of Industrial and Business Economics | | | | Wrong study design |
| Herd immunity or suppression strategy to combat COVID-19 | Jung, F.; Krieger, V.; Hufert, F. T.; Kupper, J. H. | 2020 | Clinical Hemorheology & Microcirculation | 75 | 1 | 13-17 | Wrong study design |
| How we should respond to the Coronavirus SARS-CoV-2 outbreak: A German perspective | Jung, F.; Krieger, V.; Hufert, F. T.; Kupper, J. H. | 2020 | Clinical Hemorheology & Microcirculation | 74 | 4 | 363-372 | Wrong study design |
| Coronavirus infections and deaths by poverty status: The effects of social distancing | Jung, J; Manley, J; Shrestha, V | 2021 | Journal of Economic Behavior & Organization | 182 |  | 311-330 | Wrong intervention |
| Labor Demand in the time of COVID-19: Evidence from vacancy postings and UI claims | Kahn, L; Lange, F; Wiczer, Dd | 2020 |  |  |  |  | Wrong intervention |
| Mental Distress and Psychological Disorders Related to COVID-19 Mandatory Lockdown | Kakaje, A.; Fadel, A.; Makki, L.; Ghareeb, A.; Al Zohbi, R. | 2021 | Frontiers in Public Health | 9 |  | 585235 | Wrong outcome |
| The impact of the COVID-19 pandemic on marginalized populations in the United States: A research agenda | Kantamneni, N. | 2020 | Journal of Vocational Behavior | | | 103439 | Wrong study design |
| A Simple Decision Analysis of a Mandatory Lockdown Response to the COVID-19 Pandemic | Karnon, J. | 2020 | Applied Health Economics & Health Policy | 18 | 3 | 329-331 | Wrong study design |
| Exploring the Growth of COVID-19 Cases using Exponential Modelling Across 42 Countries and Predicting Signs of Early Containment using Machine Learning | Kasilingam, D.; Prabhakaran, S. P. S.; Dinesh Kumar, R.; Rajagopal, V.; Santhosh Kumar, T.; Soundararaj, A. | 2020 | Transboundary & Emerging Diseases | 4 |  | 4 | Wrong outcome |
| Comparing Associations of State Reopening Strategies with COVID-19 Burden | Kaufman, B. G.; Whitaker, R.; Mahendraratnam, N.; Smith, V. A.; McClellan, M. B. | 2020 | Journal of General Internal Medicine | 35 | 12 | 3627-3634 | Wrong intervention |
| Understanding COVID-19 transmission, health impacts and mitigation: timely social distancing is the key | Kaur, S.; Bherwani, H.; Gulia, S.; Vijay, R.; Kumar, R. | 2020 | Environment, Development and Sustainability | | | | Wrong study design |
| Anticipating the Novel Coronavirus Disease (COVID-19) Pandemic | Kaur, T.; Sarkar, S.; Chowdhury, S.; Sinha, S. K.; Jolly, M. K.; Dutta, P. S. | 2020 | Frontiers in Public Health | 8 |  | 569669 | Wrong intervention |
| Dysphonia and Vocal Tract Discomfort While Working From Home During COVID-19 | Kenny, C. | 2020 | Journal of Voice | 16 |  | 16 | Wrong outcome |
| Forecasting the daily and cumulative number of cases for the COVID-19 pandemic in India | Khajanchi, S.; Sarkar, K. | 2020 | Chaos | 30 | 7 |  | Wrong comparator |
| COVID-19 pandemic: Lessons learned and future directions | Khanna, R. C.; Cicinelli, M. V.; Gilbert, S. S.; Honavar, S. G.; Murthy, G. S. V. | 2020 | Indian Journal of Ophthalmology | 68 | 5 | 703-710 | Wrong study design |
| Are Lockdown Measures Effective Against COVID-19? | Kharroubi, S.; Saleh, F. | 2020 | Frontiers in Public Health | 8 |  | 549692 | Wrong study design |
| Failure in initial stage containment of global COVID-19 epicenters | Khosrawipour, V.; Lau, H.; Khosrawipour, T.; Kocbach, P.; Ichii, H.; Bania, J.; Mikolajczyk, A. | 2020 | Journal of Medical Virology | 92 | 7 | 863-867 | Wrong intervention |
| Development of severe psychological distress among low-income individuals during the COVID-19 pandemic: Longitudinal study | Kikuchi, H; Machida, M; Nakamura, I; et al | 2021 | BJPsych Open Vol 7 2021, ArtID e50 | 7 |  |  | Wrong outcome |
| Why lockdown? Why national unity? Why global solidarity? Simplified arithmetic tools for decision-makers, health professionals, journalists and the general public to explore containment options for the 2019 novel coronavirus | Killeen, G. F.; Kiware, S. S. | 2020 | Infectious Disease Modelling | 5 |  | 442-458 | Wrong study design |
| Suicidal ideation during the COVID-19 pandemic: The role of insomnia | Killgore, W. D. S.; Cloonan, S. A.; Taylor, E. C.; Fernandez, F.; Grandner, M. A.; Dailey, N. S. | 2020 | Psychiatry Research | 290 |  | 113134 | Wrong comparator |
| Psychological resilience during the COVID-19 lockdown | Killgore, W. D. S.; Taylor, E. C.; Cloonan, S. A.; Dailey, N. S. | 2020 | Psychiatry Research | 291 |  | 113216 | Wrong comparator |
| Evaluating the Mental Health Impacts of the COVID-19 Pandemic in Urban South Africa: Perceived Risk of COVID-19 Infection and Childhood Trauma Predict Adult Depressive Symptoms | Kim, A. W.; Nyengerai, T.; Mendenhall, E. | 2020 | MedRxiv : the Preprint Server for Health Sciences | 16 |  | 16 | Wrong comparator |
| COVID-19 restrictions and mental distress among American adults: evidence from Corona Impact Survey (W1 and W2) | Kim, Harris Hyun-Soo; Laurence, James | 2020 | Journal of Public Health | 42 | 4 | 704-711 | Wrong comparator |
| Work Environment Surrounding COVID-19 Outbreak in Call Center, South Korea | Kim, T. | 2020 | Emerging Infectious Diseases | 26 | 10 | 29 | Wrong study design |
| Covid-19 pandemic, uganda's story | Kitara, D. L.; Ikoona, E. N. | 2020 | Pan African Medical Journal | 35 | Supplement 2 | 3-Jan | Wrong study design |
| A behavioral economic risk aversion experiment in the context of the COVID-19 pandemic | Kluwe-Schiavon, B.; Viola, T. W.; Bandinelli, L. P.; et al | 2021 | PLoS ONE [Electronic Resource] | 16 | 1 | e0245261 | Wrong outcome |
| Lockdown of 1.3 billion people in India during Covid-19 pandemic: A survey of its impact on mental health | Kochhar, A. S.; Bhasin, R.; Kochhar, G. K.; Dadlani, H.; Mehta, V. V.; Kaur, R.; Bhasin, C. K. | 2020 | Asian Journal of Psychiatry | 54 |  | 102213 | Wrong comparator |
| Disentangling Policy Effects Using Proxy Data: Which Shutdown Policies Affected Unemployment during the COVID-19 Pandemic? | Kong, Edward; Prinz, Daniel | 2020 | Journal of Public Economics | 189 |  |  | Wrong outcome |
| Mental Health Effects of COVID-19 Pandemia: A Review of Clinical and Psychological Traits | Kontoangelos, K.; Economou, M.; Papageorgiou, C. | 2020 | Psychiatry Investigation | 17 | 6 | 491-505 | Wrong study design |
| Interventions to mitigate early spread of SARS-CoV-2 in Singapore: a modelling study | Koo, J. R.; Cook, A. R.; Park, M.; et al | 2020 | The Lancet Infectious Diseases | 20 | 6 | 678-688 | Wrong study design |
| Business disruptions from social distancing | Koren, M.; Peto, R. | 2020 | PLoS ONE [Electronic Resource] | 15 | 9 | e0239113 | Wrong outcome |
| â€œSmartâ€ quarantine and â€œblanketâ€ quarantine: the Czech response to the COVID-19 pandemic | KouÅ™il, P.; FerenÄuhovÃ¡, S. | 2020 | Eurasian Geography and Economics | | |  | Wrong study design |
| Does the coronavirus pandemic level the gender inequality curve? (It doesn't) | Kristal, T.; Yaish, M. | 2020 | Research in Social Stratification and Mobility | 68 |  |  | Wrong study design |
| A causal framework to determine the effectiveness of dynamic quarantine policy to mitigate COVID-19 | Kristjanpoller, W.; Michell, K.; Minutolo, M. C. | 2021 | Applied Soft Computing | 104 |  | 107241 | Wrong study design |
| Macroeconomic Dynamics and Reallocation in an Epidemic | Krueger, Dirk; Uhlig, Harald; Xie, Taojun | 2020 |  |  |  |  | Wrong intervention |
| Drawing inference from nationwide lockdown as a response towards novel Cornavirus-19 (CoVID-19) epidemic in India | Kumar, D.; Raina, S. K.; Chauhan, R.; Kumar, P.; Sharma, S. | 2020 | Journal of Family Medicine & Primary Care | 9 | 9 | 4507-4511 | Wrong study design |
| The psychological impact of COVID-19 pandemic and lockdown on the migrant workers: A cross-sectional survey | Kumar, K.; Mehra, A.; Sahoo, S.; Nehra, R.; Grover, S. | 2020 | Asian Journal of Psychiatry | 53 |  | 102252 | Wrong comparator |
| Bringing the developmental state back in: explaining South Koreaâ€™s successful management of COVID-19 | Kumar, R. | 2021 | Third World Quarterly | |  |  | Wrong study design |
| The chronicle of COVID-19: possible strategies to curb the pandemic | Kumar, R.; Harilal, S.; Al-Sehemi, A. G.; Mathew, G. E.; Carradori, S.; Mathew, B. | 2020 | Current Medicinal Chemistry | 2 |  | 2 | Wrong study design |
| Impact of lockdown measures during COVID-19 on air quality- A case study of India | Kumari, P.; Toshniwal, D. | 2020 | International Journal of Environmental Health Research | | | 8-Jan | Wrong outcome |
| Estimating effectiveness of preventing measures for 2019 novel coronavirus diseases (COVID-19) | Kurahashi, S. | 2020 | Transactions of the Japanese Society for Artificial Intelligence | 35 | 3 |  | Language not English |
| Checking the Path Towards Recovery from the COVID-19 Isolation Response | Kydland, Finn E.; Martinez-Garcia, Enrique | 2020 |  |  |  | 34-34 | Wrong outcome |
| Effectiveness of preventive measures against COVID-19: A systematic review of In Silico modeling studies in indian context | Lahiri, A.; Jha, S. S.; Bhattacharya, S.; Ray, S.; Chakraborty, A. | 2020 | Indian Journal of Public Health | 64 | Supplement | S156-S167 | Wrong study design |
| Mental Health Nurses locked out while Australia locks down | Lakeman, R. | 2020 | Journal of psychiatric and mental health nursing. | 17 |  |  | Wrong study design |
| Predicting optimal lockdown period with parametric approach using three-phase maturation SIRD model for COVID-19 pandemic | Lalwani, S.; Sahni, G.; Mewara, B.; Kumar, R. | 2020 | Chaos, Solitons and Fractals | 138 |  |  | Wrong study design |
| Association between SARS-CoV-2 infection, exposure risk and mental health among a cohort of essential retail workers in the USA | Lan, F. Y.; Suharlim, C.; Kales, S. N.; Yang, J. | 2021 | Occupational & Environmental Medicine | 78 | 4 | 237-243 | Wrong study design |
| 24th Collegium Ramazzini Statement: Prevention of Work-Related Infection in the COVID-19 Pandemic | Landrigan, P. | 2020 | Journal of Occupational & Environmental Medicine | 19 |  | 19 | Wrong study design |
| COVID-19 does not stop at open borders: Spatial contagion among local authority districts during England's first wave | Laroze, D.; Neumayer, E.; Plumper, T. | 2021 | Social Science & Medicine | 270 |  | 113655 | Wrong intervention |
| Timing of Community Mitigation and Changes in Reported COVID-19 and Community Mobility - Four U.S. Metropolitan Areas, February 26-April 1, 2020 | Lasry, A.; Kidder, D.; Hast, M.; et al | 2020 | MMWR - Morbidity & Mortality Weekly Report | 69 | 15 | 451-457 | Wrong comparator |
| The positive impact of lockdown in Wuhan on containing the COVID-19 outbreak in China | Lau, H.; Khosrawipour, V.; Kocbach, P.; Mikolajczyk, A.; Schubert, J.; Bania, J.; Khosrawipour, T. | 2020 | Journal of Travel Medicine | 27 | 3 | 18 | Wrong intervention |
| Covid-19: Concerns and behaviours in croatia | Lauri Korajlija, Anita; Jokic-Begic, Natasa | 2020 | British Journal of Health Psychology | | | No Pagination Specified | Wrong comparator |
| The heterogeneous age-mixing model of estimating the covid cases of different local government units in the National Capital Region, Philippines | Laurio Dizon, R. | 2020 | Clinical Epidemiology and Global Health. | | | | Wrong comparator |
| First three months of COVID-19 in Croatia, Slovenia, Serbia and Federation of Bosnia and Herzegovina - comparative assessment of disease control measures | Lazic, N.; Lazic, V.; Kolaric, B. | 2020 | Infektoloski Glasnik | 40 | 2 | 43-49 | Wrong study design |
| Overview of rapid mitigating strategies in Singapore during the COVID-19 pandemic | Lee, W. C.; Ong, C. Y. | 2020 | Public Health | 185 |  | 15-17 | Wrong study design |
| Association of Country-wide Coronavirus Mortality with Demographics, Testing, Lockdowns, and Public Wearing of Masks | Leffler, C. T.; Ing, E.; Lykins, J. D.; Hogan, M. C.; McKeown, C. A.; Grzybowski, A. | 2020 | American Journal of Tropical Medicine & Hygiene | 103 | 6 | 2400-2411 | Wrong comparator |
| Global socio-economic losses and environmental gains from the Coronavirus pandemic | Lenzen, M.; Li, M.; Malik, A.; Pomponi, F.; et al | 2020 | PLoS ONE [Electronic Resource] | 15 | 7 | e0235654 | Wrong outcome |
| Filling the gaps on stroke research: Focus on inflammation and immunity | Levard, D; Buendia, I; Lanquetin, A; Glavan, M; Vivien, D; Rubio, M | 2021 | Brain, Behavior, and Immunity | 91 |  | 649-667 | Wrong outcome |
| COVID-19: Preliminary data on the impact of social distancing on loneliness and mental health | Lewis, Katie | 2020 | Journal of Psychiatric Practice | 26 | 5 | 400-404 | Wrong outcome |
| Disparities in COVID-19 Incidence, Hospitalizations, and Testing, by Area-Level Deprivation - Utah, March 3-July 9, 2020 | Lewis, N. M.; Friedrichs, M.; Wagstaff, S.; et al | 2020 | MMWR - Morbidity & Mortality Weekly Report | 69 | 38 | 1369-1373 | Wrong intervention |
| Confidence in coping with COVID-19 and its related factors among the public in Taiwan | Li, Dian-Jeng; Ko, Nai-Ying; Chen, Yi-Lung; Wang, Peng-Wei; Chang, Yu-Ping; Yen, Cheng-Fang | 2020 | Psychiatry and Clinical Neurosciences | 74 | 11 | 608-610 | Wrong outcome |
| Effects of sources of social support and resilience on the mental health of different age groups during the COVID-19 pandemic | Li, Fugui; Luo, Sihui; Mu, Weiqi; et al | 2021 | BMC Psychiatry Vol 21 2021, ArtID 16 | 21 |  |  | Wrong intervention |
| Basic reproduction number and predicted trends of coronavirus disease 2019 epidemic in the mainland of China | Li, Y.; Wang, L. W.; Peng, Z. H.; Shen, H. B. | 2020 | Infectious Diseases of Poverty | 9 | 1 | 94 | Wrong study design |
| Causal Analysis of Health Interventions and Environments for Influencing the Spread of COVID-19 in the United States of America | Li, Z.; Xu, T.; Zhang, K.; Deng, H. W.; Boerwinkle, E.; Xiong, M. | 2021 | Frontiers in Applied Mathematics and Statistics | 6 |  |  | Wrong intervention |
| Effects of policies and containment measures on control of COVID19 epidemic in Chongqing | Liang, X. H.; Tang, X.; Luo, Y. T.; Zhang, M.; Feng, Z. P. | 2020 | World Journal of Clinical Cases | 8 | 14 | 2959-2976 | Wrong comparator |
| The neglected health of international migrant workers in the COVID-19 epidemic | Liem, A.; Wang, C.; Wariyanti, Y.; Latkin, C. A.; Hall, B. J. | 2020 | The Lancet Psychiatry | 7 | 4 | e20 | Wrong study design |
| Analysis of the SARS-CoV-2 epidemic in Italy: The role of local and interventional factors in the control of the epidemic | Lilleri, D.; Zavaglio, F.; Gabanti, E.; Gerna, G.; Arbustini, E. | 2020 | PLoS ONE [Electronic Resource] | 15 | 11 | e0242305 | Wrong outcome |
| A conceptual model for the coronavirus disease 2019 (COVID-19) outbreak in Wuhan, China with individual reaction and governmental action | Lin, Q.; Zhao, S.; Gao, D.; Lou, Y.; et al | 2020 | International Journal of Infectious Diseases | 93 |  | 211-216 | Wrong intervention |
| Spread and Impact of COVID-19 in China: A Systematic Review and Synthesis of Predictions From Transmission-Dynamic Models | Lin, Y. F.; Duan, Q.; Zhou, Y.; et al | 2020 | Frontiers in Medicine | 7 |  | 321 | Wrong study design |
| Health risks and potential remedies during prolonged lockdowns for coronavirus disease 2019 (COVID-19) | Lippi, G.; Henry, B. M.; Bovo, C.; Sanchis-Gomar, F. | 2020 | Diagnosis | 7 | 2 | 85-90 | Wrong study design |
| COVID-19 Isolation in Healthy Population in Israel: Challenges in Daily Life, Mental Health, Resilience, and Quality of Life | Lipskaya-Velikovsky, L. | 2021 | International Journal of Environmental Research & Public Health [Electronic Resource] | 18 | 3 | 23 | Wrong comparator |
| COVID-19 in older people: a rapid clinical review | Lithander, Fiona E.; Neumann, Sandra; Tenison, Emma; et al | 2020 | Age & Ageing | 49 | 4 | 501-515 | Wrong study design |
| A new SAIR model on complex networks for analysing the 2019 novel coronavirus (COVID-19) | Liu, C.; Wu, X.; Niu, R.; Wu, X.; Fan, R. | 2020 | Nonlinear Dynamics | |  |  | Wrong intervention |
| Sustainable COVID-19 Mitigation: Wuhan Lockdowns, Health Inequities, and Patient Evacuation | Liu, L. | 2020 | International Journal of Health Policy & Management | 28 |  | 28 | Wrong intervention |
| COVID-19 pandemic: Experiences in China and implications for its prevention and treatment worldwide | Liu, N. N.; Tan, J. C.; Li, J.; Li, S.; Cai, Y.; Wang, H. | 2020 | Current Cancer Drug Targets | 20 | 6 | 410-416 | Wrong study design |
| COVID-19 pandemic: every day feels like a weekday to most | Liu, T.; Meyerhoff, J.; Mohr, D. C.; Ungar, L. H.; Kording, K. P. | 2020 | MedRxiv : the Preprint Server for Health Sciences | 18 |  | 18 | Wrong intervention |
| Pandemic, Mobile Payment, and Household Consumption: Micro-Evidence from China | Liu, T.; Pan, B.; Yin, Z. | 2020 | Emerging Markets Finance and Trade | 56 | 10 | 2378-2389 | Wrong intervention |
| What are the Underlying Transmission Patterns of COVID-19 Outbreak? - An Age-specific Social Contact Characterization | Liu, Y.; Gu, Z.; Xia, S.; Shi, B.; Zhou, X. N.; Shi, Y.; Liu, J. | 2020 | EClinicalMedicine | |  | 100354 | Wrong study design |
| Infection Density and Epidemic Size of COVID-19 in China outside the Hubei province | Liu, Y.; Qin, J.; Fan, Y.; Zhou, Y.; Follmann, D. A.; Huang, C. Y. | 2020 | MedRxiv : the Preprint Server for Health Sciences | 28 |  | 28 | Wrong outcome |
| Association between state stay-at-home orders and risk reduction behaviors and mental distress amid the COVID-19 pandemic | Liu, Ying; Mattke, Soeren | 2020 | Preventive Medicine: An International Journal Devoted to Practice and Theory Vol 141 2020, ArtID 106299 | 141 |  |  | Wrong study design |
| Estimates of the ongoing need for social distancing and control measures post-"lockdown" from trajectories of COVID-19 cases and mortality | Lonergan, M.; Chalmers, J. D. | 2020 | European Respiratory Journal | 56 | 1 | 7 | Wrong intervention |
| Psychological well-being among older adults during the COVID-19 outbreak: a comparative study of the young-old and the old-old adults | Lopez, J.; Perez-Rojo, G.; Noriega, C.; et al | 2020 | International Psychogeriatrics | | | 6-Jan | Wrong comparator |
| The end of social confinement and COVID-19 re-emergence risk | Lopez, L.; Rodo, X. | 2020 | Nature Human Behaviour | 4 | 7 | 746-755 | Wrong study design |
| Psychological distress associated with the COVID-19 pandemic and suppression measures during the first wave in Belgium | Lorant, V.; Smith, P.; Van den Broeck, K.; Nicaise, P. | 2021 | BMC Psychiatry | 21 | 1 | 112 | Wrong study design |
| COVID-19 Outbreak Associated with Air Conditioning in Restaurant, Guangzhou, China, 2020 | Lu, J.; Gu, J.; Li, K.; Xu, C.; Su, W.; Lai, Z.; Zhou, D.; Yu, C.; Xu, B.; Yang, Z. | 2020 | Emerging Infectious Diseases | 26 | 7 | 1628-1631 | Wrong intervention |
| The psychological and mental impact of coronavirus disease 2019 (COVID-19) on medical staff and general public - A systematic review and meta-analysis | Luo, M.; Guo, L.; Yu, M.; Jiang, W.; Wang, H. | 2020 | Psychiatry Research | 291 |  | 113190 | Wrong comparator |
| CHINA AND WORLD OUTPUT IMPACT OF THE HUBEI LOCKDOWN DURING THE CORONAVIRUS OUTBREAK | Luo, S.; Tsang, K. P. | 2020 | Contemporary Economic Policy | | |  | Wrong outcome |
| A Class Analysis of the Expansion of COVID-19 in Peru: The Case of Metropolitan Lima | Lust, J. | 2021 | Critical Sociology | |  |  | Wrong outcome |
| Measuring "Fearonomic Effects" in Valuing Therapies: An Application to COVID-19 in China | Ma, S.; Kim, D. D.; Cohen, J. T.; Neumann, P. J. | 2020 | Value in Health. | |  |  | Wrong study design |
| Epidemic Trend of COVID-19 Transmission in India During Lockdown-1 Phase | Mahajan, Pooja; Kaushal, Jyotsna | 2020 | Journal of Community Health | 45 | 6 | 1291-1300 | Wrong outcome |
| Covid-19: Leicester's lockdown to ease this weekend as infection rates fall | Mahase, E. | 2020 | BMJ | 370 |  | m2897 | Wrong study design |
| Covid-19: How does local lockdown work, and is it effective? | Mahase, E. | 2020 | BMJ | 370 |  | m2679 | Wrong study design |
| A COVID-19 Risk Assessment for the US Labor Force | Maher, S.; Hill, A. E.; Britton, P.; et al | 2020 | MedRxiv : the Preprint Server for Health Sciences | 17 |  | 17 | Wrong intervention |
| Forecasting epidemic spread of COVID-19 in India using arima model and effectiveness of lockdown | Maheshwari, H.; Yadav, D.; Chandra, U.; Rai, D. S. | 2020 | Advances in Mathematics: Scientific Journal | 9 | 6 | 3417-3430 | Wrong comparator |
| A motivational standpoint of job insecurity effects on organizational citizenship behaviors: A generational study | Mahmoud, A. B.; Reisel, W. D.; Fuxman, L.; Mohr, I. | 2021 | Scandinavian Journal of Psychology | 62 | 2 | 267-275 | Wrong intervention |
| COVID-19 pandemic and lockdown: cause of sleep disruption, depression, somatic pain, and increased screen exposure of office workers and students of India | Majumdar, P.; Biswas, A.; Sahu, S. | 2020 | Chronobiology International | | | 10-Jan | Wrong outcome |
| Social distancing: A non-pharmacological intervention for COVID-19 | Mal, P. R.; Suneel, P.; Shomeeta, P. | 2020 | Journal of the Pakistan Medical Association | 70 | 5 | S21-S24 | Wrong study design |
| Overcoming the security risks of remote working | Malecki, F. | 2020 | Computer Fraud and Security | 2020 | 7 | 12-Oct | Wrong study design |
| Effectiveness of contact tracing and quarantine on reducing COVID-19 transmission: a retrospective cohort study | Malheiro, R.; Figueiredo, A. L.; Magalhaes, J. P.; et al | 2020 | Public Health | 189 |  | 54-59 | Wrong intervention |
| The Potential Impact of COVID-19 on GDP and Trade : A Preliminary Assessment | Maliszewska, Maryla; Mattoo, Aaditya; Van Der Mensbrugghe, Dominique | 2020 |  |  |  |  | Wrong study design |
| Determinants of Social Distancing and Economic Activity during COVID-19 : A Global View | Maloney, William F.; Taskin, Temel | 2020 |  |  |  |  | Wrong outcome |
| A model based study on the dynamics of COVID-19: Prediction and control | Mandal, M.; Jana, S.; Nandi, S. K.; Khatua, A.; Adak, S.; Kar, T. K. | 2020 | Chaos Solitons & Fractals | |  | 109889 | Wrong intervention |
| A Body Tracking-Based Low-Cost Solution for Monitoring Workers' Hygiene Best Practices during Pandemics | Manghisi, V. M.; Fiorentino, M.; Boccaccio, A.; Gattullo, M.; et al | 2020 | Sensors | 20 | 21 | 29 | Wrong outcome |
| COVID-19 pandemic sheds light on the importance of food safety practices: risks, global recommendations, and perspectives | Maragoni-Santos, C.; Serrano Pinheiro de Souza, T.; Matheus, J. R. V.; et al | 2021 | Critical Reviews in Food Science & Nutrition | | | 13-Jan | Wrong study design |
| Pathways to COVID-19 'community protection' | Marais, B. J.; Sorrell, T. C. | 2020 | International Journal of Infectious Diseases | 96 |  | 496-499 | Wrong study design |
| Adherence to behavioral Covid-19 mitigation measures strongly predicts mortality | Margraf, J.; Brailovskaia, J.; Schneider, S. | 2021 | PLoS ONE [Electronic Resource] | 16 | 3-Mar |  | Wrong intervention |
| COVID-19 and Inequity: a Comparative Spatial Analysis of New York City and Chicago Hot Spots | Maroko, A. R.; Nash, D.; Pavilonis, B. T. | 2020 | Journal of Urban Health | 97 | 4 | 461-470 | Wrong intervention |
| Quantifying the effects of quarantine using an IBM SEIR model on scalefree networks | Marquioni, V. M.; de Aguiar, M. A. M. | 2020 | Chaos, Solitons and Fractals | 138 |  |  | Wrong intervention |
| Analysis of the evolution of the Sars-Cov-2 in Italy, the role of the asymptomatics and the success of Logistic model | Martelloni, G.; Martelloni, G. | 2020 | Chaos Solitons & Fractals | 140 |  | 110150 | Wrong study design |
| The essential business conundrum in COVID-19 hotspots | Martin, Christine I.; Moverman, Michael A.; Menendez, Mariano E. | 2020 | Journal of the National Medical Association | 112 | 6 | 619-620 | Wrong study design |
| Lockdown measures and relative changes in the age-specific incidence of SARS-CoV-2 in Spain | Martinez de Salazar, P.; Gomez-Barroso, D.; Pampaka, D.; et al | 2020 | MedRxiv : the Preprint Server for Health Sciences | 2 |  | 2 | Wrong outcome |
| The impact of time to impose lockdown on COVID-19 cases and deaths in European countries | Martinez-Valero, C.; Miranda, J. D.; Martin-Sanchez, F. J. | 2020 | Medicina Clinica. | 155 | 10 | 459-460 | Wrong study design |
| Modeling the Novel Coronavirus (SARS-CoV-2) Outbreak in Sicily, Italy | Maugeri, A.; Barchitta, M.; Battiato, S.; Agodi, A. | 2020 | International Journal of Environmental Research & Public Health [Electronic Resource] | 17 | 14 | 9 | Wrong study design |
| Lockdown-type measures look effective against covid-19 | May, T. | 2020 | BMJ | 370 |  | m2809 | Wrong study design |
| SARS-CoV-2 epidemic in India: epidemiological features and in silico analysis of the effect of interventions | Mazumder, A.; Arora, M.; Bharadiya, V.; et al | 2020 | F1000Research | 9 |  | 315 | Wrong study design |
| Mitigating lockdown challenges in response to COVID-19 in Sub-Saharan Africa | Mboera, L. E. G.; Akipede, G. O.; Banerjee, A.; et al | 2020 | International Journal of Infectious Diseases | 96 |  | 308-310 | Wrong study design |
| Notes From the Field: Vape Shop Business Operations Compliance in the Wake of COVID-19 | Medel, D.; Meza, L.; Galimov, A.; Baezconde-Garbanati, L.; Sussman, S. | 2020 | Evaluation & the Health Professions | 43 | 2 | 135-137 | Wrong outcome |
| Changes in lifestyle behaviours during the COVID-19 confinement in Spanish children: A longitudinal analysis from the MUGI project | Medrano, M; Cadenas-Sanchez, C; Oses, M; Arenaza, L; Amasene, M; Labayen, I | 2021 | Pediatric Obesity Vol 16(4), 2021, ArtID e12731 | 16 | 4 |  | Wrong population |
| A longitudinal study of the influence of concerns about contagion on negative affect during the COVID-19 lockdown in adults: The moderating effect of gender and resilience | Megias-Robles, A.; Gutierrez-Cobo, M. J.; Cabello, R.; Gomez-Leal, R.; Fernandez-Berrocal, P. | 2021 | Journal of Health Psychology | | | 1.36E+15 | Wrong outcome |
| Alternating quarantine for sustainable epidemic mitigation | Meidan, D.; Schulmann, N.; Cohen, R.; et al | 2021 | Nature communications | 12 | 1 | 220 | Wrong study design |
| Intermittent occupancy combined with ventilation: An efficient strategy for the reduction of airborne transmission indoors | Melikov, A. K.; Ai, Z. T.; Markov, D. G. | 2020 | Science of the Total Environment | 744 |  | 140908 | Wrong study design |
| Should governments continue lockdown to slow the spread of covid-19? | Melnick, E. R.; Ioannidis, J. P. A. | 2020 | BMJ | 369 |  | m1924 | Wrong study design |
| Impact of lockdown on COVID-19 prevalence and mortality during 2020 pandemic: observational analysis of 27 countries | Meo, S. A.; Abukhalaf, A. A.; Alomar, A. A.; AlMutairi, F. J.; Usmani, A. M.; Klonoff, D. C. | 2020 | European Journal of Medical Research | 25 | 1 | 56 | Wrong study design |
| Evaluation of the COVID-19 pandemic using an algorithm based on the Bateman function: Prediction of disease progression using observational data for the city of Heidelberg, Germany | Merle, U.; Lasmann, A.; Dressel, A. R.; Braun, P. | 2020 | International Journal of Clinical Pharmacology & Therapeutics | 58 | 7 | 366-374 | Wrong population |
| Mortality impacts of the coronavirus disease (COVID-19) outbreak by sex and age: rapid mortality surveillance system, Italy, 1 February to 18 April 2020 | Michelozzi, P.; de'Donato, F.; Scortichini, M.; et al | 2020 | Euro Surveillance: Bulletin Europeen sur les Maladies Transmissibles = European Communicable Disease Bulletin | 25 | 19 | 5 | Wrong intervention |
| CL4 Concerns, Quality of Life, Access to Care and Productivity of the General Population during the First 8 WEEKS of the Coronavirus Lockdown in Belgium and the Netherlands | Michels, R.; Goossens, L. M. A.; Bruin, R.; van Ballegooijen, H.; Krol, M. | 2020 | Value in Health | 23 (Supplement 2) | | S400 | Wrong study design |
| Optimal Epidemic Suppression under an ICU Constraint | Miclo, Laurent; Spiro, Daniel; Weibull, Jorgen | 2020 |  |  |  |  | Wrong setting |
| Psychological aspects of employment instability during the COVID-19 pandemic | Mimoun, E.; Ben Ari, A.; Margalit, D. | 2020 | Psychological Trauma:Theory, Pesearch, Practice and Policy | 12 | S1 | S183-S185 | Wrong intervention |
| Analysing the behaviour of doubling rates in 8 major countries affected by COVID-19 virus | Mishra, D.; Haleem, A.; Javaid, M. | 2020 | Journal of Oral Biology & Craniofacial Research | 10 | 4 | 478-483 | Wrong comparator |
| Understanding heterogeneity to inform the public health response to COVID-19 in Canada | Mishra, S.; Kwong, J. C.; Chan, A. K.; Baral, S. D. | 2020 | Cmaj | 192 | 25 | E684-E685 | Wrong study design |
| The effectiveness of social distancing in containing Covid-19 | Moosa, I. A. | 2020 | Applied Economics | |  | 14-Jan | Wrong outcome |
| Differences and similarities between the impact of the first and the second COVID-19-lockdown on mental health and safety behaviour in Germany | Moradian, S.; Bauerle, A.; Schweda, A.; et al | 2021 | Journal of Public Health | 26 |  | 26 | Wrong comparator |
| How can airborne transmission of COVID-19 indoors be minimised? | Morawska, L.; Tang, J. W.; Bahnfleth, W.; et al | 2020 | Environment International | 142 |  | 105832 | Wrong study design |
| Characterization of Home Working Population during COVID-19 Emergency: A Cross-Sectional Analysis | Moretti, A.; Menna, F.; Aulicino, M.; Paoletta, M.; Liguori, S.; Iolascon, G. | 2020 | International Journal of Environmental Research & Public Health [Electronic Resource] | 17 | 17 | 28 | Wrong comparator |
| Lockdown During COVID-19: The Greek Success | Moris, D.; Schizas, D. | 2020 | In Vivo | 34 | 3 Suppl | 1695-1699 | Wrong intervention |
| 2020: The Year of the COVID-19 Pandemic | Morley, J. E. | 2021 | Journal of Nutrition, Health and Aging | 25 | 1 |  | Wrong study design |
| A model for COVID-19 transmission in Connecticut | Morozova, O.; Li, Z. R.; Crawford, F. W. | 2020 | MedRxiv : the Preprint Server for Health Sciences | 16 |  | 16 | Wrong outcome |
| A Preliminary Evaluation of the Public Risk Perception Related to the COVID-19 Health Emergency in Italy | Motta Zanin, G.; Gentile, E.; Parisi, A.; Spasiano, D. | 2020 | International Journal of Environmental Research & Public Health [Electronic Resource] | 17 | 9 | 27 | Wrong outcome |
| Impact of the coronavirus outbreak on mental health in the different Spanish regions | Moya-Lacasa, C.; Alvarez-Vazquez, C. M.; Gonzalez-Blanco, L.; et al | 2021 | Actas Espanolas de Psiquiatria | 49 | 2 | 64-70 | Wrong comparator |
| Strategies to control and prevent novel coronavirus 2019: A quick overview | Muhammad, A.; Ali, J.; Manan, A.; Khan, H.; Owais, M. | 2020 | Journal of the Liaquat University of Medical and Health Sciences | 19 | 1 | 5-Jan | Wrong study design |
| Disparities, desperation, and divisiveness: Coping with COVID-19 in India | Mukherjee, S. | 2020 | Psychological Trauma:Theory, Pesearch, Practice and Policy | 12 | 6 | 582-584 | Wrong study design |
| Agony of the laborers and daily wagers during the COVID-19 induced lockdown in India | Mukhra, R.; Krishan, K.; Kanchan, T. | 2020 | Acta Bio-Medica de l Ateneo Parmense | 91 | 4 | e2020141 | Wrong study design |
| Psychological health during the coronavirus disease 2019 pandemic outbreak | Mukhtar, S. | 2020 | International Journal of Social Psychiatry | 66 | 5 | 512-516 | Wrong study design |
| A science-based response to COVID-19 | Muliyil, J. P. | 2020 | Indian Journal of Public Health | 64 | Supplement | S90 | Wrong study design |
| COVID-19 Control: Can Germany Learn From China? | Muller, O.; Lu, G.; Jahn, A.; Razum, O. | 2020 | International Journal of Health Policy & Management | 27 |  | 27 | Wrong study design |
| COVID-19 Awareness and Preparedness of Minnesota and Wisconsin Dairy Farms | Mung Ting Yung, S.; Vazquez, R. C.; Liebman, A.; et al | 2021 | Journal of Agromedicine | 9 |  | 9 | Wrong outcome |
| Covid-19-Beyond virology: Potentials for maintaining mental health during lockdown | Munk, A. J. L.; Schmidt, N. M.; Alexander, N.; Henkel, K.; Hennig, J. | 2020 | PLoS ONE [Electronic Resource] | 15 | 8 | e0236688 | Wrong comparator |
| Does social isolation aggravate psychiatric problem - popular perception in covid-19 lockdown | Muralidharan, V. A.; Lakshmanan, G.; dr.Gheena, S. | 2020 | European Journal of Molecular and Clinical Medicine | 7 | 1 | 2438-2450 | Wrong intervention |
| On the role of governmental action and individual reaction on COVID-19 dynamics in South Africa: A mathematical modelling study | Mushayabasa, S.; Ngarakana-Gwasira, E. T.; Mushanyu, J. | 2020 | Informatics in Medicine Unlocked | 20 (no pagination) | 100387 |  | Wrong study design |
| From Easing Lockdowns to Scaling-Up Community-Based COVID-19 Screening, Testing, and Contact Tracing in Africa - Shared Approaches, Innovations, and Challenges to Minimize Morbidity and Mortality | Nachega, J. B.; Grimwood, A.; Mahomed, H.; et al | 2020 | Clinical Infectious Diseases | 31 |  | 31 | Wrong study design |
| Strategies to deal with the COVID-19 pandemic | Nadanovsky, P.; Santos, Appd | 2020 | Pesquisa Odontologica Brasileira = Brazilian Oral Research | 34 |  | e068 | Wrong study design |
| Pakistan's response to COVID-19 pandemic and efficacy of quarantine and partial lockdown: A review | Nafees, M.; Khan, F. | 2020 | Electronic Journal of General Medicine | 17 (6) (no pagination) | em240 |  | Wrong intervention |
| COVID-19 and On-site Dining in Tokyo: A Time-series Analysis Using Mobile Phone Location Data | Nakanishi, M.; Shibasaki, R.; Yamasaki, S.; et al | 2021 | JMIR MHealth and UHealth | 11 |  | 11 | Wrong intervention |
| Are countries with lockdown and social distancing implementation showing a decline in COVID-19 cases? | Nayak, S. B.; Chaudhary, O. | 2020 | Journal of Clinical and Diagnostic Research | 14 | 7 | LE01-LE03 | Wrong study design |
| Early lessons from a second COVID-19 lockdown in Leicester, UK | Nazareth, J.; Minhas, J. S.; Jenkins, D. R.; et al | 2020 | Lancet | 396 | 10245 | e4-e5 | Wrong study design |
| Delay discounting of compliance with containment measures during the COVID-19 outbreak: a survey of the Italian population | Nese, M.; Riboli, G.; Brighetti, G.; et al | 2020 | Journal of Public Health | |  |  | Wrong comparator |
| Acute mental health responses during the COVID-19 pandemic in Australia | Newby, J. M.; O'Moore, K; Tang, S; Christensen, H; Faasse, K | 2020 | PLoS ONE Vol 15(7), 2020, ArtID e0236562 | 15 | 7 |  | Wrong comparator |
| To lockdown? When to peak? Will there be an end? A macroeconomic analysis on COVID-19 epidemic in the United States | Ng, W. L. | 2020 | Journal of Macroeconomics | 65 |  | 103230 | Wrong study design |
| Non-pharmaceutical strategies win coronavirus disease 2019 battle in New Zealand | Nhamo, G.; Kandawasvika, G. Q.; Sibanda, M. | 2020 | Jamba | 12 | 1 | 1010 | Wrong study design |
| Mental health and health behaviours before and during the initial phase of the COVID-19 lockdown: longitudinal analyses of the UK Household Longitudinal Study | Niedzwiedz, C. L.; Green, M. J.; Benzeval, M.; et al | 2021 | Journal of Epidemiology & Community Health | 75 | 3 | 224-231 | Wrong outcome |
| Psycho-social factors associated with the nationwide lockdown in India during COVID- 19 pandemic | Nilima, N.; Kaushik, S.; Tiwary, B.; Pandey, P. K. | 2020 | Clinical Epidemiology and Global Health. | | | | Wrong outcome |
| A mathematical model to guide the re-opening of economies during the COVID-19 pandemic | Noorbhai, H. | 2020 | Annals of Medicine & Surgery | 57 |  | 6-May | Wrong study design |
| Risk Factors Underlying COVID-19 Lockdown-Induced Mental Distress | Novotny, J. S.; Gonzalez-Rivas, J. P.; Kunzova, S.; et al | 2020 | Frontiers in psychiatry Frontiers Research Foundation | 11 |  | 603014 | Wrong comparator |
| Impact of the Burden of COVID-19 in Italy: Results of Disability-Adjusted Life Years (DALYs) and Productivity Loss | Nurchis, M. C.; Pascucci, D.; Sapienza, M.; et al | 2020 | International Journal of Environmental Research & Public Health [Electronic Resource] | 17 | 12 | 13 | Wrong intervention |
| Quarantine alone or in combination with other public health measures to control COVIDâ€19: a rapid review | Nussbaumer-Streit, B.; Mayr, V.; Dobrescu, AIulia; et al | 2020 | Cochrane Database of Systematic Reviews | | 4 |  | Wrong intervention |
| A rapid review of mental and physical health effects of working at home: how do we optimise health? | Oakman, J.; Kinsman, N.; Stuckey, R.; Graham, M.; Weale, V. | 2020 | BMC Public Health | 20 | 1 | 1825 | Wrong study design |
| Modeling the lockdown relaxation protocols of the Philippine government in response to the COVID-19 pandemic: An intuitionistic fuzzy DEMATEL analysis | Ocampo, L.; Yamagishi, K. | 2020 | Socio-Economic Planning Sciences | | |  | Wrong outcome |
| COVID-19 lockdown impact on lifestyle habits of Italian adults | Odone, A.; Lugo, A.; Amerio, A.; et al | 2020 | Acta Bio-Medica de l Ateneo Parmense | 91 | 9-S | 87-89 | Wrong comparator |
| Covid-19: England could need another lockdown in winter, say government's chief advisers | O'Dowd, A. | 2020 | BMJ | 370 |  | m2909 | Wrong study design |
| Covid-19: Easing lockdown could risk second spike, say public health doctors | O'Dowd, A. | 2020 | BMJ | 369 |  | m2193 | Wrong study design |
| Evolutionary trends of the COVID-19 epidemic and effectiveness of government interventions in Nigeria: A data-driven analysis | Odukoya, O. O.; Adeleke, I. A.; Jim, C. S.; et al | 2020 | MedRxiv : the Preprint Server for Health Sciences | 2 |  | 2 | Wrong outcome |
| Puppy love in the time of Corona: Dog ownership protects against loneliness for those living alone during the COVID-19 lockdown | Oliva, J. L.; Johnston, K. L. | 2020 | International Journal of Social Psychiatry | | | 2.08E+13 | Wrong comparator |
| Asymptomatic SARS-CoV-2 infection | Ooi, E. E.; Low, J. G. | 2020 | The Lancet Infectious Diseases | 20 | 9 | 996-998 | Wrong study design |
| Spatially explicit models for exploring COVID-19 lockdown strategies | O'Sullivan, D.; Gahegan, M.; Exeter, D. J.; Adams, B. | 2020 | Transactions in GIS | |  |  | Wrong outcome |
| Socially optimal lockdown and travel restrictions for fighting communicable virus including COVID-19 | Oum, T. H.; Wang, K. | 2020 | Transport Policy | 96 |  | 94-100 | Wrong study design |
| Impact of lockdown on Covid-19 case fatality rate and viral mutations spread in 7 countries in Europe and North America | Pachetti, M.; Marini, B.; Giudici, F.; Benedetti, F.; et al | 2020 | Journal of Translational Medicine | 18 | 1 | 338 | Wrong outcome |
| Investigating the dynamics of COVID-19 pandemic in India under lockdown | Pai, C.; Bhaskar, A.; Rawoot, V. | 2020 | Chaos Solitons & Fractals | 138 |  | 109988 | Wrong study design |
| Inter nation social lockdown versus medical care against COVID-19, a mild environmental insight with special reference to India | Paital, B.; Das, K.; Parida, S. K. | 2020 | Science of the Total Environment | 728 |  | 138914 | Wrong study design |
| The moderating roles of psychological flexibility and inflexibility on the mental health impacts of COVID-19 pandemic and lockdown in Italy | Pakenham, K. I.; Landi, G.; Boccolini, G.; Furlani, A.; Grandi, S.; Tossani, E. | 2020 | Journal of Contextual Behavioral Science | 17 |  | 109-118 | Wrong comparator |
| Association of Public Health Interventions With the Epidemiology of the COVID-19 Outbreak in Wuhan, China | Pan, A.; Liu, L.; Wang, C.; et al | 2020 | Jama | 10 |  | 10 | Wrong study design |
| Transmission dynamics and control strategies of covid-19 in wuhan, china | Pang, L.; Liu, S.; Zhang, X.; Tian, T.; Zhao, Z. | 2020 | Journal of Biological Systems | (no pagination) | 2020 |  | Wrong study design |
| Americans' COVID-19 Stress, Coping, and Adherence to CDC Guidelines | Park, C. L.; Russell, B. S.; Fendrich, M.; Finkelstein-Fox, L.; Hutchison, M.; Becker, J. | 2020 | Journal of General Internal Medicine | 35 | 8 | 2296-2303 | Wrong comparator |
| Mental Health Through the COVID-19 Quarantine: A Growth Curve Analysis on Italian Young Adults | Parola, A.; Rossi, A.; Tessitore, F.; Troisi, G.; Mannarini, S. | 2020 | Frontiers in Psychology | 11 |  | 567484 | Wrong population |
| [Covid-19: factors associated with emotional distress and psychological morbidity in spanish population.] | Parrado-Gonzalez, A.; Leon-Jariego, J. C. | 2020 | Revista Espanola de Salud Publica | 94 |  | 8 | Language not English |
| The economic cost of COVID-19: A potential pandemic impact on Indian economy | Parth, K. | 2020 | International Journal of Advanced Science and Technology | 29 | 6 Special Issue | 2182-2192 | Wrong study design |
| Impact of nonpharmacological interventions on COVID-19 transmission dynamics in India | Patel, P.; Athotra, A.; Vaisakh, T. P.; et al | 2020 | Indian Journal of Public Health | 64 | Supplement | S142-S146 | Wrong intervention |
| COVID-19: Recent updates on SARS-CoV-2 and preventing its community transmission in India by 21 days lockdown | Pawar, S. K.; Mohite, S. T. | 2020 | Journal of Pure and Applied Microbiology | 14 | Supplement 1 | 921-929 | Wrong study design |
| Outbreak dynamics of COVID-19 in China and the United States | Peirlinck, M.; Linka, K.; Sahli Costabal, F.; Kuhl, E. | 2020 | Biomechanics & Modeling in Mechanobiology | 27 |  | 27 | Wrong intervention |
| Linking key intervention timings to rapid declining effective reproduction number to quantify lessons against COVID-19 | Peng, Z.; Song, W.; Ding, Z.; et al | 2020 | Fronteras en Medicina | 4 |  | 4 | Wrong study design |
| COVID-19 outbreak response, a dataset to assess mobility changes in Italy following national lockdown | Pepe, E.; Bajardi, P.; Gauvin, L.; et al | 2020 | Scientific Data | 7 | 1 | 230 | Wrong intervention |
| Wearable Activity Trackers for Monitoring Adherence to Home Confinement During the COVID-19 Pandemic Worldwide: Data Aggregation and Analysis | Pepin, J. L.; Bruno, R. M.; Yang, R. Y.; Vercamer, V.; Jouhaud, P.; Escourrou, P.; Boutouyrie, P. | 2020 | Journal of Medical Internet Research | 22 | 6 | e19787 | Wrong outcome |
| Bergamo and Covid-19: How the Dark Can Turn to Light | Perico, N.; Fagiuoli, S.; Di Marco, F.; et al | 2021 | Frontiers in Medicine | 8 |  | 609440 | Wrong intervention |
| Non-pharmaceutical interventions during the COVID-19 pandemic: A review | Perra, N. | 2021 | Physics Reports Review Section of Physics Letters | 13 |  | 13 | Wrong study design |
| Are we all in this together? | Peters, R. C. | 2020 | Pharmaceutical Technology | 44 | 6 | 10 | Wrong study design |
| COVID-19-We urgently need to start developing an exit strategy | Petersen, E.; Wasserman, S.; Lee, S. S.; et al | 2020 | International Journal of Infectious Diseases | 96 |  | 233-239 | Wrong study design |
| Universal weekly testing as the UK COVID-19 lockdown exit strategy | Peto, J.; Alwan, N. A.; Godfrey, K. M.; et al | 2020 | Lancet | 395 | 10234 | 1420-1421 | Wrong study design |
| Weekly COVID-19 testing with household quarantine and contact tracing is feasible and would probably end the epidemic | Peto, J.; Carpenter, J.; Smith, G. D.; et al | 2020 | Royal Society Open Science | 7 | 6 | 200915 | Wrong study design |
| The first 100 days of SARS-CoV-2 control in Vietnam | Pham, Q. T.; Rabaa, M. A.; Duong, H. L.; et al | 2020 | Clinical Infectious Diseases | 1 |  | 1 | Wrong comparator |
| Country Responses and the Reaction of the Stock Market to COVID-19â€”a Preliminary Exposition | Phan, D. H. B.; Narayan, P. K. | 2020 | Emerging Markets Finance and Trade | 56 | 10 | 2138-2150 | Wrong outcome |
| COVID-19 Emergency Sick Leave Has Helped Flatten The Curve In The United States | Pichler, S.; Wen, K.; Ziebarth, N. R. | 2020 | Health Affairs | 39 | 12 | 2197-2204 | Wrong comparator |
| Covid-19: which country has the most effective lockdown? | Pick, A. | 2020 | BMJ | 370 |  | m3036 | Wrong study design |
| The effect of age, gender, income, work, and physical activity on mental health during coronavirus disease (COVID-19) lockdown in Austria | Pieh, C.; Budimir, S.; Probst, T. | 2020 | Journal of Psychosomatic Research | 136 |  | 110186 | Wrong comparator |
| Mental health before and during the COVID-19 pandemic: a longitudinal probability sample survey of the UK population | Pierce, M.; Hope, H.; Ford, T.; et al | 2020 | The Lancet. Psychiatry. | 21 |  | 21 | Wrong outcome |
| Mental health before and during the COVID-19 pandemic: a longitudinal probability sample survey of the UK population | Pierce, M.; Hope, H.; Ford, T.; et al | 2020 | The Lancet Psychiatry | 7 | 10 | 883-892 | Wrong outcome |
| Nudges against pandemics: Swedenâ€™s COVID-19 containment strategy in perspective | Pierre, J. | 2020 | Policy and Society | 39 | 3 | 478-493 | Wrong study design |
| Dramatic reduction of psychiatric emergency consultations during lockdown linked to COVID-19 in Paris and suburbs | Pignon, B.; Gourevitch, R.; Tebeka, S.; et al | 2020 | Psychiatry & Clinical Neurosciences | 1 |  | 1 | Wrong outcome |
| The effect of lockdown regulations on SARS-CoV-2 infectivity in Gauteng Province, South Africa | Pillai, J.; Motloba, P.; Motaung, K. S. C.; et al | 2020 | South African Medical Journal | 110 | 11 | 1119-1123 | Wrong study design |
| Tracing and analysis of 288 early SARS-CoV-2 infections outside China: A modeling study | Pinotti, F.; Di Domenico, L.; Ortega, E.; et al | 2020 | PLoS Medicine / Public Library of Science | 17 | 7 | e1003193 | Wrong intervention |
| Staying home, staying safe? A short-term analysis of covid-19 on dallas domestic violence | Piquero, Alex R.; Riddell, Jordan R.; Bishopp, Stephen A.; et al | 2020 | American Journal of Criminal Justice | | | No Pagination Specified | Wrong outcome |
| Nepal's Response to Contain COVID-19 Infection | Piryani, R. M.; Piryani, S.; Shah, J. N. | 2020 | Journal of Nepal Health Research Council | 18 | 1 | 128-134 | Wrong study design |
| The Post-Lockdown Era: What Is Next in Italy? | Pomara, C.; Li Volti, G.; Cappello, F. | 2020 | Frontiers in Pharmacology | 11 (no pagination) | 1074 |  | Wrong study design |
| Job loss and mental health during the COVID-19 lockdown: Evidence from South Africa | Posel, D.; Oyenubi, A.; Kollamparambil, U. | 2021 | PLoS ONE [Electronic Resource] | 16 | 3 | e0249352 | Wrong intervention |
| Community prevalence of SARS-CoV-2 in England from April to November, 2020: results from the ONS Coronavirus Infection Survey | Pouwels, K. B.; House, T.; Pritchard, E.; Robotham, J. V.; et al | 2021 | The lancet. Public Health | 6 | 1 | e30-e38 | Wrong intervention |
| Organizational climate, opportunities, challenges and psychological wellbeing of the remote working employees during covid-19 pandemic: A general linear model approach with reference to information technology industry in Hyderabad | Prasad, K. D. V.; Mangipudi, M. R.; Vaidya, R. W.; Muralidhar, B. | 2020 | International Journal of Advanced Research in Engineering and Technology | 11 | 4 | 372-389 | Wrong comparator |
| The psychological impact of COVID-19 pandemic lockdowns: a review and meta-analysis of longitudinal studies and natural experiments | Prati, G.; Mancini, A. D. | 2021 | Psychological Medicine | 51 | 2 | 201-211 | Wrong study design |
| The effect of control strategies to reduce social mixing on outcomes of the COVID-19 epidemic in Wuhan, China: a modelling study | Prem, K.; Liu, Y.; Russell, T. W.; et al | 2020 | The lancet. Public Health | 5 | 5 | e261-e270 | Wrong study design |
| Brazilian child protection professionals' resilient behavior during the COVID-19 pandemic | Priolo Filho, S. R.; Goldfarb, D; Zibetti, M. R.; Aznar-Blefari, C | 2020 | Child Abuse & Neglect Vol 110(Part 2), 2020, ArtID 104701 | 110 | Part 2 |  | Wrong outcome |
| 'Distancers' and 'non-distancers'? The potential social psychological impact of moralizing COVID-19 mitigating practices on sustained behaviour change | Prosser, A. M. B.; Judge, M.; Bolderdijk, J. W.; Blackwood, L.; Kurz, T. | 2020 | British Journal of Social Psychology | 59 | 3 | 653-662 | Wrong study design |
| Covid-19: India imposes lockdown for 21 days and cases rise | Pulla, P. | 2020 | BMJ | 368 |  | m1251 | Wrong study design |
| A GPS Data-Based Index to Determine the Level of Adherence to COVID-19 Lockdown Policies in India | Puppala, H.; Bheemaraju, A.; Asthana, R. | 2021 | Journal Of Healthcare Informatics Research | | | 17-Jan | Wrong outcome |
| Develop risk and assesment procedure for anticipating covid-19 in food industries | Purwanto, A.; Supono, J.; Rahayu, P.; Ponda, H.; Fatma, N. F.; Fahlevi, M. | 2020 | Journal of Critical Reviews | 7 | 15 | 1991-2004 | Wrong outcome |
| Application of seir model in covid-19 and the effect of lockdown on reducing the number of active cases | Putra, Z. A.; Abidin, S. A. Z. | 2020 | Indonesian Journal of Science and Technology | 5 | 2 | 185-192 | Wrong intervention |
| Physical Activity, Screen Time, and Emotional Well-Being during the 2019 Novel Coronavirus Outbreak in China | Qin, F.; Song, Y.; Nassis, G. P.; et al | 2020 | International Journal of Environmental Research & Public Health [Electronic Resource] | 17 | 14 | 17 | Wrong comparator |
| Impacts of social and economic factors on the transmission of coronavirus disease 2019 (COVID-19) in China | Qiu, Y.; Chen, X.; Shi, W. | 2020 | Journal of Population Economics | | | Jan-46 | Wrong study design |
| Living With COVID-19: A Systemic and Multi-Criteria Approach to Enact Evidence-Based Health Policy | Raboisson, D.; Lhermie, G. | 2020 | Frontiers in Public Health | 8 |  | 294 | Wrong study design |
| Assessment of the COVID-19 pandemic situation: Data from two countries with different security measures taken by authorities (Belarus and Russia) | Radchikova, N. P.; Odintsova, M. A. | 2021 | Data in Brief | 35 |  | 106917 | Wrong study design |
| Epidemiology of sars-cov-2 in Egypt | Radwan, G. N. | 2020 | Eastern Mediterranean Health Journal | 26 | 7 | 768-773 | Wrong intervention |
| A Review of the Strategies and Studies on the Prevention and Control of the New Coronavirus in Workplaces | Rafeemanesh, E.; Ahmadi, F.; Memarzadeh, M. | 2020 | Archives of Bone & Joint Surgery | 8 | Suppl1 | 242-246 | Wrong study design |
| Data-driven dynamic clustering framework for mitigating the adverse economic impact of Covid-19 lockdown practices | Rahman, M. A.; Zaman, N.; Asyhari, A. T.; et al | 2020 | Sustainable Cities and Society | 62 |  |  | Wrong outcome |
| Working from home-Who is happy? A survey of Lithuania's employees during the COVID-19 quarantine period | RaiÅ¡iene, A. G.; Rapuano, V.; VarkuleviÄiute, K.; StachovÃ¡, K. | 2020 | Sustainability (Switzerland) | 12 | 13 |  | Wrong comparator |
| A dynamic modeling tool for estimating healthcare demand from the COVID19 epidemic and evaluating population-wide interventions | Rainisch, G.; Undurraga, E. A.; Chowell, G. | 2020 | International Journal of Infectious Diseases | 96 |  | 376-383 | Wrong study design |
| Probable Exit Strategy Against COVID-19 of Low Resource Country like Nepal: Open Floor Discussion | Rajbhandari, B.; Gurung, M.; Poudel, L.; Shrestha, A.; Karmacharya, B. M. | 2020 | Jnma, Journal of the Nepal Medical Association | 58 | 224 | 286-292 | Wrong study design |
| An insight into the mental health needs of the common public of Tamil Nadu, India, subjected to COVID-19 lockdown, a rare unprecedented crisis | Rajendran, K. P.; Govindarajulu, S.; Adaikalam, J. M.; et al | 2021 | Annales Medico Psychologiques. | | |  | Wrong comparator |
| COVID-19 and mental health: A review of the existing literature | Rajkumar, R. P. | 2020 | Asian Journal of Psychiatry | 52 (no pagination) | 102066 |  | Wrong comparator |
| Tackling Corona Virus Disease 2019 (COVID 19) in Workplaces | Ramesh, N.; Siddaiah, A.; Joseph, B. | 2020 | The Indian Journal of Occupational & Environmental Medicine | 24 | 1 | 16-18 | Wrong study design |
| The Economic Implications of COVID-19 in the United States | Ramgobin, D.; Benson, J.; Kalayanamitra, R.; et al | 2020 | South Dakota Medicine: The Journal of the South Dakota State Medical Association | 73 | 5 | 218-222 | Wrong study design |
| A longitudinal study of mental health before and during COVID-19 lockdown in the French population | Ramiz, L.; Contrand, B.; Rojas Castro, M. Y.; et al | 2021 | Global Health | 17 | 1 | 29 | Wrong population |
| Navigating disruptive crises through service-led growth: The impact of COVID-19 on Italian manufacturing firms | Rapaccini, M.; Saccani, N.; Kowalkowski, C.; Paiola, M.; Adrodegari, F. | 2020 | Industrial Marketing Management | 88 |  | 225-237 | Wrong comparator |
| How and When to End the COVID-19 Lockdown: An Optimization Approach | Rawson, T.; Brewer, T.; Veltcheva, D.; Huntingford, C.; Bonsall, M. B. | 2020 | Frontiers in Public Health | 8 |  | 262 | Wrong intervention |
| Predictions, role of interventions and effects of a historic national lockdown in India's response to the COVID-19 pandemic: data science call to arms | Ray, D.; Salvatore, M.; Bhattacharyya, R.; et al | 2020 | Harvard Data Science Review | 1 |  |  | Wrong study design |
| India's Lockdown : An Interim Report | Ray, Debraj; Subramanian, S. | 2020 |  |  |  |  | Wrong study design |
| The â€œEye of the Hurricaneâ€ Paradox: An Unexpected and Unequal Rise of Well-Being During the Covid-19 Lockdown in France | Recchi, E.; Ferragina, E.; Helmeid, E.; et al | 2020 | Research in Social Stratification and Mobility | 68 |  |  | Wrong intervention |
| Depression, Anxiety and Stress Among Indians in Times of Covid-19 Lockdown | Rehman, U.; Shahnawaz, M. G.; Khan, N. H.; et al | 2020 | Community Mental Health Journal | 23 |  | 23 | Wrong comparator |
| Pandemic and lockdown: a territorial approach to COVID-19 in China, Italy and the United States | Ren, X. | 2020 | Eurasian Geography and Economics | | |  | Wrong study design |
| Mental Health During the Covid-19 Outbreak in China: a Meta-Analysis | Ren, X.; Huang, W.; Pan, H.; Huang, T.; Wang, X.; Ma, Y. | 2020 | Psychiatric Quarterly | 8 |  | 8 | Wrong comparator |
| Predicting the second wave of COVID-19 in Washtenaw County, MI | Renardy, M.; Kirschner, D. E. | 2020 | MedRxiv : the Preprint Server for Health Sciences | 7 |  | 7 | Wrong study design |
| Forecasting COVID-19-Associated Hospitalizations under Different Levels of Social Distancing in Lombardy and Emilia-Romagna, Northern Italy: Results from an Extended SEIR Compartmental Model | Reno, C.; Lenzi, J.; Navarra, A.; et al | 2020 | Journal of Clinical Medicine | 9 | 5 | 15 | Wrong comparator |
| Coping and tolerance of uncertainty: Predictors and mediators of mental health during the COVID-19 pandemic | Rettie, H.; Daniels, J. | 2020 | American Psychologist | 3 |  | 3 | Wrong comparator |
| Changing workplace geographies in the COVID-19 crisis | Reuschke, D.; Felstead, A. | 2020 | Dialogues in Human Geography | 10 | 2 | 208-212 | Wrong study design |
| Estimates of the Potential Impact of the COVID-19 Pandemic on Sexual and Reproductive Health In Low- and Middle-Income Countries | Riley, T.; Sully, E.; Ahmed, Z.; Biddlecom, A. | 2020 | International perspectives on sexual & reproductive health | 46 |  | 73-76 | Wrong study design |
| Covid-19: UK prime minister announces relaxation of lockdown and social distancing rules | Rimmer, A. | 2020 | BMJ | 369 |  | m2526 | Wrong study design |
| Adjusting working conditions and evaluating the risk of infection during the COVID-19 pandemic in different workplace settings in Germany: a study protocol for an explorative modular mixed methods approach | Rind, E.; Kimpel, K.; Preiser, C.; et al | 2020 | BMJ Open | 10 | 11 | e043908 | Wrong study design |
| Associations of Social Isolation with Anxiety and Depression During the Early COVID-19 Pandemic: A Survey of Older Adults in London, UK | Robb, C. E.; de Jager, C. A.; Ahmadi-Abhari, S.; et al | 2020 | Frontiers in psychiatry Frontiers Research Foundation | 11 |  | 591120 | Wrong population |
| Clinicians, cooks, and cashiers: Examining health equity and the COVID-19 risks to essential workers | Roberts, J. D.; Dickinson, K. L.; Koebele, E.; et al | 2020 | Toxicology & Industrial Health | 36 | 9 | 689-702 | Wrong comparator |
| Why is it difficult to accurately predict the COVID-19 epidemic? | Roda, W. C.; Varughese, M. B.; Han, D.; Li, M. Y. | 2020 | Infectious Disease Modelling | 5 |  | 271-281 | Wrong study design |
| [Psychosocial Impact of Quarantine Measures During Serious Coronavirus Outbreaks: A Rapid Review] | Rohr, S.; Muller, F.; Jung, F.; Apfelbacher, C.; Seidler, A.; Riedel-Heller, S. G. | 2020 | Psychiatrische Praxis | 47 | 4 | 179-189 | Language not English |
| Is the Cure Worse than the Problem Itself? Immediate Labor Market Effects of COVID-19 Case Rates and School Closures in the U.S | Rojas, F. L; Jiang, X; Montenovo, L; Simon, K. I.; Weinberg, B. A.; Wing, C | 2020 |  |  |  |  | Wrong intervention |
| A Predictive Study of Resilience and Its Relationship with Academic and Work Dimensions during the COVID-19 Pandemic | Roman-Mata, S. S.; Zurita-Ortega, F.; Puertas-Molero, P.; Badicu, G.; Gonzalez-Valero, G. | 2020 | Journal of Clinical Medicine | 9 | 10 | 12 | Wrong comparator |
| Health and economic measures in response to the COVID-19 pandemic- Effect on street vendors | Romero-Michel, J. C.; Mokay-Ramirez, K. A.; Delgado-Machuca, M.; et al | 2021 | Journal of Infection in Developing Countries | 15 | 2 | 198-203 | Wrong intervention |
| Real-time forecasts of the COVID-19 epidemic in China from February 5th to February 24th, 2020 | Roosa, K.; Lee, Y.; Luo, R.; et al | 2020 | Infectious Disease Modelling | 5 |  | 256-263 | Wrong intervention |
| Impact of Lockdown on the Epidemic Dynamics of COVID-19 in France | Roques, L.; Klein, E. K.; Papaix, J.; Sar, A.; Soubeyrand, S. | 2020 | Frontiers in Medicine | 7 |  | 274 | Wrong comparator |
| Early Phase of the COVID-19 Outbreak in Hungary and Post-Lockdown Scenarios | Rost, G.; Bartha, F. A.; Bogya, N.; et al | 2020 | Viruses | 12 | 7 | 30 | Wrong study design |
| Association of job loss, income loss, and financial burden with adverse mental health outcomes during coronavirus disease 2019 pandemic in Thailand: A nationwide cross-sectional study | Ruengorn, C.; Awiphan, R.; Wongpakaran, N.; et al | 2021 | Depression & Anxiety | 1 |  | 1 | Wrong intervention |
| [Health statistics and invisibility by sex and gender during the COVID-19 epidemic] | Ruiz Cantero, M. T. | 2020 | Gaceta Sanitaria | 4 |  | 4 | Language not English; |
| Assessing the impact of coordinated COVID-19 exit strategies across Europe | Ruktanonchai, N. W.; Floyd, J. R.; Lai, S.; Ruktanonchai, C. W.; et al | 2020 | Science | 17 |  | 17 | Wrong study design |
| Effect of Nonpharmaceutical Interventions on Transmission of Severe Acute Respiratory Syndrome Coronavirus 2, South Korea, 2020 | Ryu, S.; Ali, S. T.; Jang, C.; Kim, B.; Cowling, B. J. | 2020 | Emerging Infectious Diseases | 26 | 10 | 2 | Wrong intervention |
| COVID-19 Pandemic: How to Use Artificial Intelligence to Choose Non-Vulnerable Workers for Positions with the Highest Possible Levels of Exposure to the Novel Coronavirus | S, A. R. M.; S, M. J. M.; H, P. | 2020 | Journal of Biomedical Physics & Engineering | 10 | 3 | 383-386 | Wrong study design |
| Analysing the Covid-19 Cases in Kerala: a Visual Exploratory Data Analysis Approach | S, J.; Sreedharan, S. | 2020 | SN Comprehensive Clinical Medicine | | | 12-Jan | Wrong study design |
| Socioeconomic Determinants of Covid-19 Infections and Mortality: Evidence from England and Wales | Sa, Filipa | 2020 |  |  |  |  | Wrong intervention |
| Employee norm-violations in the service encounter during the corona pandemic and their impact on customer satisfaction | SÃ¶derlund, M. | 2020 | Journal of Retailing and Consumer Services | 57 |  |  | Wrong intervention |
| Understanding the effects of COVID-19 on the health and safety of immigrant hospitality workers in the United States | SÃ¶nmez, S.; Apostolopoulos, Y.; Lemke, M. K.; Hsieh, Y. C. J. | 2020 | Tourism Management Perspectives | 35 |  |  | Wrong study design |
| United but divided: Policy responses and people's perceptions in the EU during the COVID-19 outbreak | Sabat, I.; Neuman-Bohme, S.; Varghese, N. E.; et al | 2020 | Health Policy | 22 |  | 22 | Wrong comparator |
| Lockdown for COVID-19 and its impact on community mobility in India: An analysis of the COVID-19 Community Mobility Reports, 2020 | Saha, J.; Barman, B.; Chouhan, P. | 2020 | Children and Youth Services Review | 116 |  |  | Wrong outcome |
| A data driven epidemic model to analyse the lockdown effect and predict the course of COVID-19 progress in India | Sahoo, B. K.; Sapra, B. K. | 2020 | Chaos, Solitons and Fractals | 139 |  |  | Wrong study design |
| COVID-19 Outbreak in Jordan: A 150Â Days of Successful Response and Re-Open Strategy | Saidan, M. N.; Al-Weshah, R. A.; Masaâ€™deh, R.; Saidan, H.; Kheirallah, K. A. | 2021 | Studies in Systems, Decision and Control | 334 |  | 81-93 | Wrong study design |
| Considerations for an Individual-Level Population Notification System for Pandemic Response: A Review and Prototype | Sakib, M. N.; Butt, Z. A.; Morita, P. P.; Oremus, M.; Fong, G. T.; Hall, P. A. | 2020 | Journal of Medical Internet Research | 22 | 6 | e19930 | Wrong study design |
| Estimating the burden of SARS-CoV-2 in France | Salje, H.; Tran Kiem, C.; Lefrancq, N.; et al | 2020 | Science | 369 | 6500 | 208-211 | Wrong outcome |
| Community Transmission of SARS-CoV-2 Associated with a Local Bar Opening Event - Illinois, February 2021 | Sami, S.; Turbyfill, C. R.; Daniel-Wayman, S.; et al | 2021 | MMWR - Morbidity & Mortality Weekly Report | 70 | 14 | 528-532 | Wrong intervention |
| Nowcasting Economic Activity in Times of COVID-19 : An Approximation from the Google Community Mobility Report | Bravo, S,; Ezequiel, J.R. ; Jooste, C | 2020 |  |  |  |  | Wrong intervention |
| An Efficient COVID-19 Prediction Model Validated with the Cases of China, Italy and Spain: Total or Partial Lockdowns? | Sanchez-Caballero, S.; Selles, M. A.; Peydro, M. A.; Perez-Bernabeu, E. | 2020 | Journal of Clinical Medicine | 9 | 5 | 20 | Wrong study design |
| [COVID-19: Quarantine, isolation, social distancing and lockdown: Are they the same?] | Sanchez-Villena, A. R.; de La Fuente-Figuerola, V. | 2020 | Anales de Pediatria | 93 | 1 | 73-74 | Language not English |
| Less social emergency departments: implementation of workplace contact reduction during COVID-19 | Sangal, R. B.; Scofi, J. E.; Parwani, V.; Pickens, A. T.; Ulrich, A.; Venkatesh, A. K. | 2020 | Emergency Medicine Journal | 24 |  | 24 | Wrong study design |
| A multicriteria approach for risk assessment of Covid-19 in urban district lockdown | Sangiorgio, V.; Parisi, F. | 2020 | Safety Science | 130 |  | 104862 | Wrong study design |
| Changes in work and life patterns associated with depressive symptoms during the COVID-19 pandemic: an observational study of health app (CALO mama) users | Sato, K.; Sakata, R.; Murayama, C.; Yamaguchi, M.; Matsuoka, Y.; Kondo, N. | 2021 | Occupational & Environmental Medicine | 22 |  | 22 | Wrong study design |
| COVID-19 and work from home: Digital transformation of the workforce | SaviÄ‡, D. | 2020 | Grey Journal | 16 | 2 | 101-104 | Wrong study design |
| A study on impact of Covid-19 over employee mental wellness | Saxena, A. | 2021 | Turkish Journal of Computer and Mathematics Education | 12 | 5 | 1585-1590 | Wrong study design |
| Canada needs to rapidly escalate public health interventions for its COVID-19 mitigation strategies | Scarabel, F.; Pellis, L.; Bragazzi, N. L.; Wu, J. | 2020 | Infectious Disease Modelling | 5 |  | 316-322 | Wrong intervention |
| Accountability for SRHR in the context of the COVID-19 pandemic | Schaaf, M.; Boydell, V.; Van Belle, S.; Brinkerhoff, D. W.; George, A. | 2020 | Sexual and Reproductive Health Matters | | | Jan-00 | Wrong study design |
| A less objectionable greed? Work-life conflict and unjust pay during a pandemic | Schieman, S.; Narisada, A. | 2021 | Research in Social Stratification & Mobility | 71 |  | 100564 | Wrong intervention |
| Agile Working during COVID-19 Pandemic | Schmidtner, M.; Doering, C.; Timinger, H. | 2021 | IEEE Engineering Management Review | | | | Wrong outcome |
| Psychological Impact of Corona Lockdown in Germany: Changes in Need Satisfaction, Well-Being, Anxiety, and Depression | Schwinger, M.; Trautner, M.; Karchner, H.; Otterpohl, N. | 2020 | International Journal of Environmental Research & Public Health [Electronic Resource] | 17 | 23 | 5 | Wrong study design |
| Cell Phone Activity in Categories of Places and Associations With Growth in Cases of COVID-19 in the US | Sehra, S. T.; George, M.; Wiebe, D. J.; Fundin, S.; Baker, J. F. | 2020 | JAMA Internal Medicine | 180 | 12 | 1614-1620 | Wrong comparator |
| COVID-19 And Racial/Ethnic Disparities In Health Risk, Employment, And Household Composition | Selden, T. M.; Berdahl, T. A. | 2020 | Health Affairs | |  | 101377hlthaff202000897 | Wrong outcome |
| Strategic measures for food processing and manufacturing facilities to combat coronavirus pandemic (COVID-19) | Shahbaz, M.; Bilal, M.; Akhlaq, M.; Moiz, A.; Zubair, S.; Iqbal, H. M. N. | 2020 | Journal of Pure and Applied Microbiology | 14 | 2 | 1087-1094 | Wrong study design |
| Food safety and COVID-19: Precautionary measures to limit the spread of Coronavirus at food service and retail sector | Shahbaz, M.; Bilal, M.; Moiz, A.; Zubair, S.; Iqbal, H. M. N. | 2020 | Journal of Pure and Applied Microbiology | 14 | 1 | 749-756 | Wrong study design |
| Covid-19: An australian perspective | Shakespeare-Finch, J; Bowen-Salter, H; Cashin, M; et al | 2020 | Journal of Loss and Trauma | | | No Pagination Specified | Wrong study design |
| Strategic assessment of COVID-19 pandemic in Bangladesh: comparative lockdown scenario analysis, public perception, and management for sustainability | Shammi, M.; Bodrud-Doza, M.; Islam, A. R. M. T.; Rahman, M. M. | 2020 | Environment, Development and Sustainability | | | | Wrong outcome |
| COVID-19 pandemic, socioeconomic crisis and human stress in resource-limited settings: A case from Bangladesh | Shammi, M.; Bodrud-Doza, M.; Towfiqul Islam, A. R. M.; Rahman, M. M. | 2020 | Heliyon | 6 | 5 | e04063 | Wrong outcome |
| Emotional distress in young adults during the COVID-19 pandemic: evidence of risk and resilience from a longitudinal cohort study | Shanahan, L.; Steinhoff, A.; Bechtiger, L.; et al | 2020 | Psychological Medicine | |  | 10-Jan | Wrong intervention |
| Coronavirus disease 2019 in India: Post-lockdown scenarios and provisioning for health care | Shankar, S.; Chatterjee, K.; Yadav, A. K. | 2020 | Medical Journal Armed Forces India. | | |  | Wrong outcome |
| Effect of Threat Control Management Strategies on Number Infected by COVID-19 | Sharieh, A.; Khurmah, R. A.; Masadeh, R.; Alzaqebah, A.; Alsharman, N.; Sharieh, F. | 2021 | Studies in Systems, Decision and Control | 334 |  | 15-41 | Wrong study design |
| Assessment of COVID-19 Pandemic in Nepal: A Lockdown Scenario Analysis | Sharma, K.; Banstola, A.; Parajuli, R. R. | 2021 | Frontiers in Public Health | 9 |  | 599280 | Wrong study design |
| The effects of the COVID-19 crisis on the subjective well-being of the Israeli population-monitored phase by phase | Shavit, T.; Sherman, A.; Aisenberg, D. | 2021 | Current Psychology | |  | 8-Jan | Wrong outcome |
| Working in a bubble: How can businesses reopen while limiting the risk of COVID-19 outbreaks? | Shaw, J.; Day, T.; Malik, N.; et al | 2020 | CMAJ Canadian Medical Association Journal | 192 | 44 | E1362-E1366 | Wrong study design |
| Novel approaches to estimate compliance with lockdown measures in the COVID-19 pandemic | Sheikh, A.; Sheikh, Z.; Sheikh, A. | 2020 | Journal of Global Health | 10 | 1 | 10348 | Wrong study design |
| A systematic approach to estimating the effectiveness of multi-scale IAQ strategies for reducing the risk of airborne infection of SARS-CoV-2 | Shen, J.; Kong, M.; Dong, B.; Birnkrant, M. J.; Zhang, J. | 2021 | Building & Environment | |  | 107926 | Wrong study design |
| Anxiety, depression, traumatic stress and COVID-19-related anxiety in the UK general population during the COVID-19 pandemic | Shevlin, M; McBride, O; Murphy, J; et al | 2020 | BJPsych Open Vol 6 2020, ArtID e125 | 6 |  |  | Wrong study design |
| Prevalence of and Risk Factors Associated With Mental Health Symptoms Among the General Population in China During the Coronavirus Disease 2019 Pandemic | Shi, L.; Lu, Z. A.; Que, J. Y.; et al | 2020 | JAMA Network Open | 3 | 7 | e2014053 | Wrong intervention |
| Effective control of SARS-CoV-2 transmission in Wanzhou, China | Shi, Q.; Hu, Y.; Peng, B.; et al | 2021 | Nature Medicine | 27 | 1 | 86-93 | Wrong study design |
| The Luxury of Lockdown | Shiva, M.; Molana, H. | 2021 | European Journal of Development Research | | | 21-Jan | Wrong study design |
| Effects of the COVID-19 pandemic and nationwide lockdown on trust, attitudes toward government, and well-being | Sibley, C. G.; Greaves, L. M.; Satherley, N.;et al | 2020 | American Psychologist | 75 | 5 | 618-630 | Wrong outcome |
| Occupational risks for SARS-CoV-2 infection: the Polish experience | Sierpinski, R.; Pinkas, J.; Jankowski, M.; Juszczyk, G.; Topor-Madry, R.; Szumowski, L. | 2020 | International Journal of Occupational Medicine & Environmental Health | 22 |  | 22 | Wrong setting |
| COVID-19 in Italy: impact of containment measures and prevalence estimates of infection in the general population | Signorelli, C.; Scognamiglio, T.; Odone, A. | 2020 | Acta Bio-Medica de l Ateneo Parmense | 91 | 3-S | 175-179 | Wrong study design |
| COVID-ABS: An agent-based model of COVID-19 epidemic to simulate health and economic effects of social distancing interventions | Silva, P. C. L.; Batista, P. V. C.; Lima, H. S.; Alves, M. A.; GuimarÃ£es, F. G.; Silva, R. C. P. | 2020 | Chaos, Solitons and Fractals | 139 |  |  | Wrong study design |
| Coping with COVID-19 in an international border region: health and economy | Silva-Sobrinho, R. A.; Zilly, A.; Silva, Rmmd; et al | 2021 | Revista Latino-Americana de Enfermagem | 29 |  | e3398 | Wrong intervention |
| Efforts to Minimize the Impact of Lockdown on Migrant Workers in India During the COVID-19 Pandemic | Singh, G. P.; Arun, P.; Chavan, B. S. | 2020 | The Primary Care Companion to CNS Disorders | 22 | 3 | 11 | Wrong study design |
| Contagion effect of COVID-19 outbreak: Another recipe for disaster on Indian economy | Singh, M. K.; Neog, Y. | 2020 | Journal of Public Affairs | |  |  | Wrong study design |
| Study of ARIMA and least square support vector machine (LS-SVM) models for the prediction of SARS-CoV-2 confirmed cases in the most affected countries | Singh, S.; Parmar, K. S.; Makkhan, S. J. S.; Kaur, J.; Peshoria, S.; Kumar, J. | 2020 | Chaos, Solitons and Fractals | 139 |  |  | Wrong intervention |
| The effect of lockdown on the outcomes of COVID-19 in Spain: An ecological study | Siqueira, C; Freitas, Y. N. L.; Cancela, M. C.; Carvalho, M.; Oliveras-Fabregas, A.; de Souza, D. L. B. | 2020 | PLoS ONE [Electronic Resource] | 15 | 7 | e0236779 | Wrong comparator |
| Only strict quarantine measures can curb the coronavirus disease (COVID-19) outbreak in Italy, 2020 | Sjodin, H.; Wilder-Smith, A.; Osman, S.; Farooq, Z.; Rocklov, J. | 2020 | Euro Surveillance: Bulletin Europeen sur les Maladies Transmissibles = European Communicable Disease Bulletin | 25 | 13 | 4 | Wrong outcome |
| Modelling Strong Control Measures for Epidemic Propagation with Networks - A COVID-19 Case Study | Small, M.; Small, M.; Small, M.; Cavanagh, D. | 2020 | IEEE Access | 8 |  | 109719-109731 | Wrong study design |
| Social relationships and depression during the COVID-19 lockdown: longitudinal analysis of the COVID-19 Social Study | Sommerlad, A.; Marston, L.; Huntley, J.; et al | 2021 | Psychological Medicine | |  | 10-Jan | Wrong intervention |
| Individual-based simulation model for COVID-19 transmission in Daegu,Korea | Son, W. S.; Team, R. | 2020 | Epidemiology and health | |  | e2020042 | Wrong intervention |
| The Impact of the Non-essential Business Closure Policy on Covid-19 Infection Rates | Song, H; McKenna, R. M.; Chen, A. T.; David, G; Smith-McLallen, A | 2021 |  |  |  |  | Wrong outcome |
| Mental Health and Work Attitudes among People Resuming Work during the COVID-19 Pandemic: A Cross-Sectional Study in China | Song, L.; Wang, Y.; Li, Z.; Yang, Y.; Li, H. | 2020 | International Journal of Environmental Research & Public Health [Electronic Resource] | 17 | 14 | 14 | Wrong comparator |
| COVID-19 Lockdown and the Behavior Change on Physical Exercise, Pain and Psychological Well-Being: An International Multicentric Study | Sonza, A.; da Cunha de Sa-Caputo, D.; Sartorio, A.; Tamini, S.; et al | 2021 | International Journal of Environmental Research & Public Health [Electronic Resource] | 18 | 7 | 6 | Wrong study design |
| Early assessment of the impact of mitigation measures to control COVID-19 in 22 French metropolitan areas, October to November 2020 | Spaccaferri, G.; Larrieu, S.; Pouey, J.; et al | 2020 | Euro Surveillance: Bulletin Europeen sur les Maladies Transmissibles = European Communicable Disease Bulletin | 25 | 50 | 12 | Wrong study design |
| A changing world | Spencer, W. | 2020 | Zentralsterilisation - Central Service | 28 | 2 | 99 | Wrong study design |
| Parents' Stress and Children's Psychological Problems in Families Facing the COVID-19 Outbreak in Italy | Spinelli, M.; Lionetti, F.; Pastore, M.; Fasolo, M. | 2020 | Frontiers in Psychology | 11 |  | 1713 | Wrong population |
| Flexible employment relationships and careers in times of the COVID-19 pandemic | Spurk, D.; Straub, C. | 2020 | Journal of Vocational Behavior | | | 103435 | Wrong study design |
| Changes in sleep schedule and chronotype due to COVID-19 restrictions and home office | Staller, N.; Randler, C. | 2020 | Somnologie |  |  | 7-Jan | Wrong outcome |
| A coming wave: Suicide and gender after covid-19 | Standish, Katerina | 2020 | Journal of Gender Studies | | | No Pagination Specified | Wrong study design |
| A phased approach to unlocking during the COVID-19 pandemic-Lessons from trend analysis | Stedman, M.; Davies, M.; Lunt, M.; Verma, A.; Anderson, S. G.; Heald, A. H. | 2020 | International Journal of Clinical Practice | | | e13528 | Wrong intervention |
| Distancing Bonus Or Downscaling Loss? The Changing Livelihood of Us Online Workers in Times of COVID-19 | Stephany, F.; Dunn, M.; Sawyer, S.; Lehdonvirta, V. | 2020 | Tijdschrift voor Economische en Sociale Geografie | 111 | 3 | 561-573 | Wrong intervention |
| South Africa: Challenges and successes of the COVID-19 lockdown | Stiegler, N.; Bouchard, J. P. | 2020 | Annales Medico Psychologiques. | | |  | Wrong outcome |
| Breaking the chain of transmission within a tertiary health service: An approach to contact tracing during the COVID-19 pandemic | Stuart, R. L.; Zhu, W.; Morand, E. F.; Stripp, A. | 2021 | Infection, Disease & Health | 26 | 2 | 118-122 | Wrong setting |
| Examining the Impact of COVID-19 Lockdown in Wuhan and Lombardy: A Psycholinguistic Analysis on Weibo and Twitter | Su, Y.; Xue, J.; Liu, X.; et al | 2020 | International Journal of Environmental Research & Public Health [Electronic Resource] | 17 | 12 | 24 | Wrong outcome |
| A real-time survey on the psychological impact of mild lockdown for COVID-19 in the Japanese population | Sugaya, N.; Yamamoto, T.; Suzuki, N.; Uchiumi, C. | 2020 | Scientific Data | 7 | 1 | 372 | Wrong comparator |
| Community pharmacy response to infection control during COVID-19. A cross-sectional survey | Sum, Z. Z.; Ow, C. J. W. | 2021 | Research In Social & Administrative Pharmacy | 17 | 1 | 1845-1852 | Wrong setting |
| Transmission dynamics of COVID-19 in Wuhan, China: effects of lockdown and medical resources | Sun, G. Q.; Wang, S. F.; Li, M. T.; et al | 2020 | Nonlinear Dynamics | |  |  | Wrong study design |
| Psychological reactions and insomnia in adults with mental health disorders during the COVID-19 outbreak | Sun, Q; Qin, Q; Basta, M; Chen, B; Li, Y | 2021 | BMC Psychiatry Vol 21 2021, ArtID 19 | 21 |  |  | Wrong study design |
| Impacts of geographic factors and population density on the COVID-19 spreading under the lockdown policies of China | Sun, Z.; Zhang, H.; Yang, Y.; Wan, H.; Wang, Y. | 2020 | Science of the Total Environment | 746 (no pagination) | 141347 |  | Wrong intervention |
| Socioeconomic disparities in subway use and COVID-19 outcomes in New York City | Sy, K. T. L.; Martinez, M. E.; Rader, B.; White, L. F. | 2020 | MedRxiv : the Preprint Server for Health Sciences | 30 |  | 30 | Wrong intervention |
| Effects of Social Distancing Measures during the First Epidemic Wave of Severe Acute Respiratory Syndrome Infection, Greece | Sypsa, V.; Roussos, S.; Paraskevis, D.; Lytras, T.; Tsiodras, S.; Hatzakis, A. | 2021 | Emerging Infectious Diseases | 27 | 2 | 452-462 | Wrong study design |
| Abilities, Motivations, and Opportunities of Furloughed Employees in the Context of Covid-19: Preliminary Evidence From the UK | Szulc, J. M.; Smith, R. | 2021 | Frontiers in Psychology | 12 |  | 635144 | Wrong study design |
| Mental health outcomes of the CoViD-19 pandemic | Talevi, D.; Socci, V.; Carai, M.; et al | 2020 | Rivista di Psichiatria | 55 | 3 | 137-144 | Wrong study design |
| Mental health of family, friends, and co-workers of COVID-19 patients in Japan | Tanoue, Y.; Nomura, S.; Yoneoka, D.; et al | 2020 | Psychiatry Research | 291 |  | 113067 | Wrong intervention |
| A glimmer of hope beyond the spring for Europe | The Lancet Infectious, Diseases | 2021 | The Lancet Infectious Diseases | 21 | 4 | 439 | Wrong study design |
| Sustaining containment of COVID-19 in China | The, Lancet | 2020 | The Lancet | 395 | 10232 | 1230 | Wrong study design |
| The plight of essential workers during the COVID-19 pandemic | The, Lancet | 2020 | The Lancet | 395 | 10237 | 1587 | Wrong study design |
| India under COVID-19 lockdown | The, Lancet | 2020 | Lancet | 395 | 10233 | 1315 | Wrong study design |
| Death in the time of coronavirus | Thomas, G. | 2020 | Indian Journal of Medical Ethics | V | 2 | 3-Jan | Wrong study design |
| Education, the science of learning, and the COVID-19 crisis | Thomas, M. S. C.; Rogers, C. | 2020 | Prospects |  |  |  | Wrong study design |
| COVID-19: Leaving lockdown-Of Schrodinger, cats, testing and masks | Thomson, G. A. | 2020 | International Journal of Clinical Practice | | | e13519 | Wrong study design |
| Covid-19: A&E visits in England fall by 25% in week after lockdown | Thornton, J. | 2020 | BMJ | 369 |  | m1401 | Wrong study design |
| Modeling analysis of COVID-19 based on morbidity data in Anhui, China | Tian, J.; Wu, J.; Bao, Y.; et al | 2020 | Mathematical Biosciences and Engineering | 17 | 4 | 2842-2852 | Wrong intervention |
| Effect of timing of implementation of containment measures on Covid-19 epidemic. The case of the first wave in Italy | Timelli, L.; Girardi, E. | 2021 | PLoS ONE [Electronic Resource] | 16 | 1 | e0245656 | Wrong study design |
| Prediction for the spread of COVID-19 in India and effectiveness of preventive measures | Tomar, A.; Gupta, N. | 2020 | Science of the Total Environment | 728 |  | 138762 | Wrong intervention |
| Covid-19: Home testing programme across England aims to help define way out of lockdown | Torjesen, I. | 2020 | BMJ | 369 |  | m1799 | Wrong study design |
| Covid-19: England plan to ease lockdown is "confusing" and "risky," say doctors | Torjesen, I. | 2020 | BMJ | 369 |  | m1877 | Wrong study design |
| Characterize health and economic vulnerabilities of workers to control the emergence of COVID-19 in an industrial zone in Vietnam | Tran, B. X.; Vu, G. T.; Latkin, C. A.; et al | 2020 | Safety Science | |  | 104811 | Wrong study design |
| A â€œnew normalityâ€ for small-scale artisanal Fishers? The case of unregulated fisheries during the COVID-19 pandemic in the BahÃ­a Blanca estuary (SW Atlantic Ocean) | Truchet, D. M.; Buzzi, N. S.; Noceti, M. B. | 2021 | Ocean and Coastal Management | 206 |  |  | Wrong outcome |
| Mental distress among U.S. adults during the COVID-19 pandemic | Twenge, Jean M.; Joiner, Thomas E. | 2020 | Journal of Clinical Psychology | 76 | 12 | 2170-2182 | Wrong outcome |
| Modeling the impact of non-pharmaceutical interventions on the dynamics of novel coronavirus with optimal control analysis with a case study | Ullah, S.; Khan, M. A. | 2020 | Chaos, Solitons and Fractals | 139 |  |  | Wrong intervention |
| Ensuring availability of food for child nutrition amidst the covid â€“ 19 pandemic: Challenges and way forward | Upadhyay, M. K.; Patra, S.; Khan, A. M. | 2020 | Indian Journal of Community Health | 32 | 2 Special Issue | 251-254 | Wrong study design |
| Impact of COVID-19 pandemic on the global economy: An evaluative study | Usha, A. A.; Dâ€™couto, R. L. | 2020 | International Journal of Psychosocial Rehabilitation | 24 | 5 | 7797-7809 | Wrong study design |
| Risk of a second wave of Covid-19 infections: using artificial intelligence to investigate stringency of physical distancing policies in North America | Vaid, S.; McAdie, A.; Kremer, R.; Khanduja, V.; Bhandari, M. | 2020 | International Orthopaedics | 44 | 8 | 1581-1589 | Wrong study design |
| [COVID19-Tracker: a shiny app to analise data on SARS-CoV-2 epidemic in Spain] | Valls, J.; Tobias, A.; Satorra, P.; Tebe, C. | 2020 | Gaceta Sanitaria | 27 |  | 27 | Language not English |
| Anxiety and depression symptoms, and lack of emotional support among the general population before and during the COVID-19 pandemic. A prospective national study on prevalence and risk factors | van der Velden, Peter G.; Contino, Carlo; Das, Marcel; van Loon, Peter; Bosmans, Mark W. | 2020 | Journal of Affective Disorders | 277 |  | 540-548 | Wrong intervention |
| Disruptions and General Distress for Essential and Nonessential Employees During the COVID-19 Pandemic | van Zoonen, W.; Ter Hoeven, C. L. | 2021 | Journal of Business & Psychology | | | 16-Jan | Wrong outcome |
| Excess mortality during the Covid-19 pandemic: Early evidence from England and Wales | Vandoros, S. | 2020 | Social Science & Medicine | 258 |  | 113101 | Wrong intervention |
| Corona citizens' science project-repeated surveys of the Irish response to COVID-19 and subsequent lockdown and restrictive measures | Vellinga, A.; Mellotte, M.; Mealy, P. J.; et al | 2021 | Irish Journal of Medical Science | 24 |  | 24 | Wrong outcome |
| Indications for healthcare surge capacity in European countries facing an exponential increase in coronavirus disease (COVID-19) cases, March 2020 | Verelst, F.; Kuylen, E.; Beutels, P. | 2020 | Euro Surveillance: Bulletin Europeen sur les Maladies Transmissibles = European Communicable Disease Bulletin | 25 | 13 | 4 | Wrong setting |
| Depression, anxiety, and stress and socio-demographic correlates among general Indian public during COVID-19 | Verma, S.; Mishra, A. | 2020 | International Journal of Social Psychiatry | | | 2.08E+13 | Wrong comparator |
| Early assessment of the impact of mitigation measures on the COVID-19 outbreak in Italy | Vicentini, C.; Bordino, V.; Gardois, P.; Zotti, C. M. | 2020 | Public Health | 185 |  | 99-101 | Wrong setting |
| Simulating the spread of COVID-19 via a spatially-resolved susceptibleâ€“exposedâ€“infectedâ€“recoveredâ€“deceased (SEIRD) model with heterogeneous diffusion | Viguerie, A.; Lorenzo, G.; Auricchio, F.; et al | 2021 | Applied Mathematics Letters | 111 |  |  | Wrong study design |
| Stress resilience during the coronavirus pandemic | Vinkers, C. H.; van Amelsvoort, T.; Bisson, J. I.; et al | 2020 | European Neuropsychopharmacology | 35 |  | 16-Dec | Wrong study design |
| Under the COVID-19 lockdown: Rapid review about the unique case of North Cyprus | Volkan, E.; Volkan, E. | 2020 | Psychological Trauma:Theory, Pesearch, Practice and Policy | 12 | 5 | 539-541 | Wrong study design |
| Coronavirus Disease 2019 (COVID-19) Epidemic and Mental Health Status in the General Adult Population of Serbia: A Cross-Sectional Study | Vujcic, I.; Safiye, T.; Milikic, B.; et al | 2021 | International Journal of Environmental Research & Public Health [Electronic Resource] | 18 | 4 | 17 | Wrong comparator |
| Covid-19: Decisive action is the hallmark of South Africa's early success against coronavirus | Wadvalla, B. A. | 2020 | The BMJ | 369 (no pagination) | m1623 |  | Wrong study design |
| Social distancing merely stabilized COVID-19 in the US | Wagner, A. B.; Hill, E. L.; Ryan, S. E.; et al | 2020 | Stat : The ISI's Journal For Rapid Dissemination of Statistics Research | | | e302 | Wrong intervention |
| COVID-19 Intervention Scenarios for a Long-term Disease Management | Wallentin, G.; Kaziyeva, D.; Reibersdorfer-Adelsberger, E. | 2020 | International Journal of Health Policy & Management | 26 |  | 26 | Wrong comparator |
| Update: COVID-19 Among Workers in Meat and Poultry Processing Facilities - United States, April-May 2020 | Waltenburg, M. A.; Victoroff, T.; Rose, C. E.; et al | 2020 | MMWR - Morbidity & Mortality Weekly Report | 69 | 27 | 887-892 | Wrong comparator |
| Socioeconomic status and well-being during COVID-19: A resource-based examination | Wanberg, C. R.; Csillag, B; Douglass, R. P.; Zhou, L; Pollard, M. S. | 2020 | Journal of Applied Psychology | 105 | 12 | 1382-1396 | Wrong intervention |
| Achieving Effective Remote Working During the COVID-19 Pandemic: A Work Design Perspective | Wang, B.; Liu, Y.; Qian, J.; Parker, S. K. | 2020 | Applied Psychology | 5 |  | 5 | Wrong outcome |
| Achieving Effective Remote Working During the COVIDâ€19 Pandemic: A Work Design Perspective | Wang, Bin; Liu, Yukun; Qian, Jing; Parker, Sharon K. | 2021 | Applied Psychology: An International Review | 70 | 1 | 16-59 | Wrong outcome |
| Depressive, anxiety, and insomnia symptoms between population in quarantine and general population during the COVID-19 pandemic: A case-controlled study | Wang, C; Song, W; Hu, X; et al | 2021 | BMC Psychiatry Vol 21 2021, ArtID 99 | 21 |  |  | Wrong intervention |
| Quantitative evaluation on control measures for an epidemic: A case study of COVID-19 | Wang, G.; Huang, N. E.; Qiao, F. | 2020 | Kexue Tongbao/Chinese Science Bulletin | 65 | 11 | 1009-1015 | Language not English |
| Improved epidemic dynamics model and its prediction for COVID-19 in Italy | Wang, H.; Xu, K.; Li, Z.; Pang, K.; He, H. | 2020 | Applied Sciences (Switzerland) | 10 | 14 |  | Wrong study design |
| Strengths, weaknesses, opportunities and threats (Swot) analysis of china's prevention and control strategy for the covid-19 epidemic | Wang, J.; Wang, Z. | 2020 | International Journal of Environmental Research and Public Health | 17 (7) (no pagination) | 2235 |  | Wrong study design |
| Epidemiology of 2019 novel coronavirus in Jiangsu Province, China after wartime control measures: A population-level retrospective study | Wang, K. W.; Gao, J.; Wang, H.; et al | 2020 | Travel Medicine & Infectious Disease | 35 |  | 101654 | Wrong intervention |
| Real-time estimation of the reproduction number of the novel coronavirus disease (COVID-19) in China in 2020 based on incidence data | Wang, K.; Zhao, S.; Li, H.; Song, Y.; et al | 2020 | Annals of Translational Medicine | 8 | 11 | 689 | Wrong intervention |
| Survival-Convolution Models for Predicting COVID-19 Cases and Assessing Effects of Mitigation Strategies | Wang, Q.; Xie, S.; Wang, Y.; Zeng, D. | 2020 | MedRxiv : the Preprint Server for Health Sciences | 13 |  | 13 | Wrong study design |
| A four-compartment model for the COVID-19 infection-implications on infection kinetics, control measures, and lockdown exit strategies | Wang, T.; Wu, Y.; Lau, J. Y. N.; et al | 2020 | Precision Clinical Medicine | 3 | 2 | 104-112 | Wrong study design |
| Analysis of Policies Based on the Multi-Fuzzy Regression Discontinuity, in Terms of the Number of Deaths in the Coronavirus Epidemic | Wang, X.; Chen, C.; Du, Y.; Zhang, Y.; Wu, C. | 2021 | Healthcare | 9 | 2 | 22 | Wrong intervention |
| Bidirectional Influence of the COVID-19 Pandemic Lockdowns on Health Behaviors and Quality of Life among Chinese Adults | Wang, X.; Lei, S. M.; Le, S.; Yang, Y.; Zhang, B.; Yao, W.; Gao, Z.; Cheng, S. | 2020 | International Journal of Environmental Research & Public Health [Electronic Resource] | 17 | 15 | 2 | Wrong outcome |
| Coping with COVID-19: Core Elements of Lockdown Wuhan City Policy | Wang, X.; Shi, L.; Zhang, Y.; Chen, H.; Sun, G. | 2021 | Journal of Health Care for the Poor & Underserved | 32 | 1 | 373-385 | Wrong study design |
| How to improve adherence with quarantine: rapid review of the evidence | Webster, R. K.; Brooks, S. K.; Smith, L. E.; Woodland, L.; Wessely, S.; Rubin, G. J. | 2020 | Public Health (Elsevier) | 182 |  | 163-169 | Wrong intervention |
| Does respiratory co-infection facilitate dispersal of SARS-CoV-2? investigation of a super-spreading event in an open-space office | Weissberg, D.; Boni, J.; Rampini, S. K.; et al | 2020 | Antimicrobial Resistance & Infection Control | 9 | 1 | 191 | Wrong study design |
| Advice from a systems-biology model of the corona epidemics | Westerhoff, H. V.; Kolodkin, A. N. | 2020 | Npj Systems Biology & Applications | 6 | 1 | 18 | Wrong outcome |
| A phenomenological approach to assessing the effectiveness of COVID-19 related nonpharmaceutical interventions in Germany | Wieland, T. | 2020 | Safety Science | 131 (no pagination) | 104924 |  | Wrong study design |
| A phenomenological approach to assessing the effectiveness of COVID-19 related nonpharmaceutical interventions in Germany | Wieland, T. | 2020 | Safety Science | 131 |  | 104924 | Wrong intervention |
| Reduction in effective reproduction number of COVID-19 is higher in countries employing active case detection with prompt isolation | Wilasang, C.; Sararat, C.; Jitsuk, N. C.; et al | 2020 | Journal of Travel Medicine | 8 |  | 8 | Wrong study design |
| Lockdown to contain COVID-19 is a window of opportunity to prevent the second wave | Wilder-Smith, A.; Bar-Yam, Y.; Fisher, D. | 2020 | Journal of Travel Medicine | 30 |  | 30 | Wrong study design |
| COVID-19 lockdown during field workâ€”Challenges and strategies in continuing the ReGES study | Will, G.; Becker, R.; Weigand, D. | 2020 | Survey Research Methods | 14 | 2 | 247-252 | Wrong study design |
| SOCRATES: an online tool leveraging a social contact data sharing initiative to assess mitigation strategies for COVID-19 | Willem, L.; Van Hoang, T.; Funk, S.; Coletti, P.; Beutels, P.; Hens, N. | 2020 | BMC Research Notes | 13 | 1 | 293 | Wrong outcome |
| Public perceptions and experiences of social distancing and social isolation during the COVID-19 pandemic: a UK-based focus group study | Williams, S. N.; Armitage, C. J.; Tampe, T.; Dienes, K. | 2020 | BMJ Open | 10 | 7 | e039334 | Wrong comparator |
| Evaluation of the english version of the fear of covid-19 scale and its relationship with behavior change and political beliefs | Winter, T; Riordan, B. C.; Pakpour, A. H.; et al | 2020 | International Journal of Mental Health and Addiction | | | No Pagination Specified | Wrong comparator |
| Covid-19: UK needs flexible local plans to come out of lockdown, say public health experts | Wise, J. | 2020 | BMJ | 369 |  | m1704 | Wrong study design |
| The great pause: a minor theory exploration of COVID-19 response in Switzerland | Wolfe, S. D. | 2020 | Eurasian Geography and Economics | | |  | Wrong study design |
| Temporal changes in psychobehavioural responses during the early phase of the COVID-19 pandemic in Malaysia | Wong, Li Ping; Alias, Haridah | 2021 | Journal of Behavioral Medicine | 44 | 1 | 18-28 | Wrong study design |
| Estimation of effects of contact tracing and mask adoption on COVID-19 transmission in San Francisco: a modeling study | Worden, L.; Wannier, R.; Blumberg, S.; Ge, A. Y.; Rutherford, G. W.; Porco, T. C. | 2020 | MedRxiv : the Preprint Server for Health Sciences | 11 |  | 11 | Wrong intervention |
| Are we all in this together? Longitudinal assessment of cumulative adversities by socioeconomic position in the first 3 weeks of lockdown in the UK | Wright, L.; Steptoe, A.; Fancourt, D. | 2020 | Journal of Epidemiology & Community Health | 5 |  | 5 | Wrong comparator |
| Workforce Survival: Tracking Potential COVID-19 Exposure Amid Socioeconomic Activities Using Automatic Log-Keeping Apps | Xia, Y. | 2020 | Population Health Management | 4 |  | 4 | Wrong study design |
| Linking key intervention timing to rapid decline of the COVID-19 effective reproductive number to quantify lessons from mainland China | Xiao, Y.; Tang, B.; Wu, J.; Cheke, R. A.; Tang, S. | 2020 | International Journal of Infectious Diseases | 97 |  | 296-298 | Wrong study design |
| Analysis of prevention measures in Shenzhen based on SIQR model during the novel coronavirus pneumonia | Xu, R.; Wang, J.; Ye, S.; Wang, X. | 2020 | Shenzhen Daxue Xuebao (Ligong Ban)/Journal of Shenzhen University Science and Engineering | 37 | 3 | 257-264 | Language not English |
| Research on COVID-19 based on ARIMA model<sup>DELTA</sup>-Taking Hubei, China as an example to see the epidemic in Italy | Yang, Q.; Wang, J.; Ma, H.; Wang, X. | 2020 | Journal of Infection and Public Health | 20 |  | 20 | Wrong intervention |
| Effectiveness of non-pharmaceutical interventions to contain COVID-19: a case study of the 2020 spring pandemic wave in New York City | Yang, W.; Shaff, J.; Shaman, J. | 2021 | Journal of the Royal Society Interface | 18 | 175 | 20200822 | Wrong study design |
| COVID-19 and restaurant demand: Early effects of the pandemic and stay-at-home orders | Yang, Yang; Liu, Hongbo; Chen, Xiang | 2020 | International Journal of Contemporary Hospitality Management | 32 | 12 | 3809-3834 | Wrong intervention |
| Globalisation in the time of COVID-19: repositioning Africa to meet the immediate and remote challenges | Yaya, S.; Otu, A.; Labonte, R. | 2020 | Global Health | 16 | 1 | 51 | Wrong study design |
| How did chinese government implement unconventional measures against COVID-19 pneumonia | Yu, X.; Li, N. | 2020 | Risk Management and Healthcare Policy | 13 |  | 491-499 | Wrong study design |
| Analysis of second outbreak of COVID-19 after relaxation of control measures in India | Yu, X.; Qi, G.; Hu, J. | 2020 | Nonlinear Dynamics | |  | 19-Jan | Wrong study design |
| Comparison of the Indicators of Psychological Stress in the Population of Hubei Province and Non-Endemic Provinces in China During Two Weeks During the Coronavirus Disease 2019 (COVID-19) Outbreak in February 2020 | Yuan, S.; Liao, Z.; Huang, H.; et al | 2020 | Medical Science Monitor | 26 |  | e923767 | Wrong comparator |
| Modelling the effects of Wuhan's lockdown during COVID-19, China | Yuan, Z.; Xiao, Y.; Dai, Z.; Huang, J.; Zhang, Z.; Chen, Y. | 2020 | Bulletin of the World Health Organization | 98 | 7 | 484-494 | Wrong intervention |
| The Covid-19 lockdown in the United Kingdom and subjective well-being: Have the self-employed suffered more due to hours and income reductions? | Yue, W.; Cowling, M. | 2021 | International Small Business Journal: Researching Entrepreneurship | 39 | 2 | 93-108 | Wrong outcome |
| The impact of covid-19 on community life in the province of Bali, Indonesia | Yuniti, I. G. A. D.; Sasmita, N.; Komara, L. L.; Purba, J. H.; Pandawani, N. P. | 2020 | International Journal of Psychosocial Rehabilitation | 24 | 10 | 1918-1929 | Wrong comparator |
| The mortality and psychological burden caused by response to COVID-19 outbreak | Yusuf, E.; Tisler, A. | 2020 | Medical Hypotheses | 143 |  | 110069 | Wrong study design |
| A Perspective of International Collaboration Through Web-Based Telecommunication-Inspired by COVID-19 Crisis | Zaer, H.; Fan, W.; Orlowski, D.; et al | 2020 | Frontiers in Human Neuroscience | 14 |  | 577465 | Wrong study design |
| Testing and lockdown; how much & to what extent?-an epidemiological dilemma | Zaman, F. A.; Kundapur, R. | 2020 | Indian Journal of Community Health | 32 | 2 Special Issue | 228-230 | Wrong study design |
| Social distancing and inequality in the United States amid COVID-19 outbreak | Zhai, W.; Liu, M.; Peng, Z. R. | 2020 | Environment and Planning A | | |  | Wrong study design |
| Integrating IAQ control strategies to reduce the risk of asymptomatic SARS CoV-2 infections in classrooms and open plan offices | Zhang, J. | 2020 | Science and Technology for the Built Environment | | | 6-Jan | Wrong study design |
| Changes in contact patterns shape the dynamics of the COVID-19 outbreak in China | Zhang, J.; Litvinova, M.; Liang, Y.; et al | 2020 | Science | 368 | 6498 | 1481-1486 | Wrong outcome |
| Unprecedented disruption of lives and work: Health, distress and life satisfaction of working adults in China one month into the COVID-19 outbreak | Zhang, S. X.; Wang, Y.; Rauch, A.; Wei, F. | 2020 | Psychiatry Research | 288 |  | 112958 | Wrong intervention |
| Assessment of Coronavirus Disease 2019 Community Containment Strategies in Shenzhen, China | Zhang, X. M.; Zhou, H. E.; Zhang, W. W.; et al | 2020 | JAMA Network Open | 3 | 6 | e2012934 | Wrong intervention |
| Predicting turning point, duration and attack rate of COVID-19 outbreaks in major Western countries | Zhang, X.; Ma, R.; Wang, L. | 2020 | Chaos Solitons & Fractals | |  | 109829 | Wrong comparator |
| Impact of COVID-19 on China's macroeconomy and agri-food system â€“ an economy-wide multiplier model analysis | Zhang, Y.; Diao, X.; Chen, K. Z.; Robinson, S.; Fan, S. | 2020 | China Agricultural Economic Review | | |  | Wrong outcome |
| [Epidemiological investigation on a cluster epidemic of COVID-19 in a collective workplace in Tianjin] | Zhang, Y.; Su, X.; Chen, W.; et al | 2020 | Chung-Hua Liu Hsing Ping Hsueh Tsa Chih Chinese Journal of Epidemiology | 41 | 5 | 648-652 | Language not English; |
| Applicability of time fractional derivative models for simulating the dynamics and mitigation scenarios of COVID-19 | Zhang, Y.; Yu, X.; Sun, H.; Tick, G. R.; Wei, W.; Jin, B. | 2020 | Chaos, Solitons and Fractals | 138 |  |  | Wrong comparator |
| Staggered release policies for COVID-19 control: Costs and benefits of relaxing restrictions by age and risk | Zhao, H.; Feng, Z. | 2020 | Mathematical Biosciences | 326 |  | 108405 | Wrong study design |
| COVID-19 infection outbreak increases anxiety level of general public in China: Involved mechanisms and influencing factors | Zhao, H; He, X; Fan, G; et al | 2020 | Journal of Affective Disorders | 276 |  | 446-452 | Wrong study design |
| Imitation dynamics in the mitigation of the novel coronavirus disease (COVID-19) outbreak in Wuhan, China from 2019 to 2020 | Zhao, S.; Stone, L.; Gao, D.; et al | 2020 | Annals of Translational Medicine | 8 | 7 | 448 | Wrong intervention |
| Analysis of the Transmissibility Change of 2019-Novel Coronavirus Pneumonia and Its Potential Factors in China from 2019 to 2020 | Zhao, Y.; Wang, R.; Li, J.; Zhang, Y.; Yang, H.; Zhao, Y. | 2020 | BioMed Research International | 2020 |  | 3842470 | Wrong intervention |
| Psychological distress in North America during COVID-19: The role of pandemic-related stressors | Zheng, J; Morstead, T; Sin, N; et al | 2021 | Social Science & Medicine Vol 270 2021, ArtID 113687 | 270 |  |  | Wrong intervention |
| Is Lockdown Bad for Social Anxiety in COVID-19 Regions?: A National Study in The SOR Perspective | Zheng, L.; Miao, M.; Lim, J.; Li, M.; Nie, S.; Zhang, X. | 2020 | International Journal of Environmental Research & Public Health [Electronic Resource] | 17 | 12 | 24 | Wrong comparator |
| A COVID-19 descriptive study of life after lockdown in Wuhan, China | Zhou, T.; Nguyen, T. T.; Zhong, J.; Liu, J. | 2020 | Royal Society Open Science | 7 | 9 | 200705 | Wrong comparator |
| Pandemic Spread-an Empirical Analysis | Ziegler, Z. | 2020 | Rambam Maimonides Medical Journal | 30 |  | 30 | Wrong intervention |
| Association of the Timing of School Closings and Behavioral Changes With the Evolution of the Coronavirus Disease 2019 Pandemic in the US | Zimmerman, F. J.; Anderson, N. W. | 2021 | JAMA Pediatrics | 175 | 5 | 501-509 | Wrong intervention |
| Our fight against the rapidly evolving COVID-19 pandemic: A review of India's actions and proposed way forward | Zodpey, S.; Negandhi, H.; Dua, A.; Vasudevan, A.; Raja, M. | 2020 | Indian Journal of Community Medicine | 45 | 2 | 117-124 | Wrong study design |

## References

5. Hoehn-Velasco L, Silverio-Murillo A, Balmori de la Miyar JR. The long downturn: The impact of the great lockdown on formal employment. *Journal of Economics and Business* 2021;115(May-June):p.105983.

30. Alfano V, Ercolano S. The efficacy of lockdown against COVID-19: A cross-country panel analysis. *Applied Health Economics & Health Policy* 2020, 18:509-517.

31. Askitas N, Tatsiramos K, Verheyden B. Estimating worldwide effects of non-pharmaceutical interventions on COVID-19 incidence and population mobility patterns using a multiple-event study. *Scientific Reports* 2021, 11:1972.

32. Castillo RC, Staguhn ED, Weston-Farber E. The effect of state-level stay-at-home orders on COVID-19 infection rates. *American journal of infection control* 2020, 48:958-960.

33. Chae SH, Park HJ. Effectiveness of penalties for lockdown violations during the COVID-19 Pandemic in Germany. *American Journal of Public Health* 2020, 110:1844-1849.

34. Cobb JS, Seale MA. Examining the effect of social distancing on the compound growth rate of COVID-19 at the county level (United States) using statistical analyses and a random forest machine learning model. *Public health* 2020, 185:27-29.

35. Courtemanche C, Garuccio J, Le A, Pinkston J, Yelowitz A. Strong social distancing measures in The United States reduced the COVID-19 growth rate. *Health Affairs* 2020, 39:1237-1246.

36. Deb P, Furceri D, Ostry JD, Tawk N. The effect of containment measures on the COVID-19 pandemic. 2020. *International Monetary Fund*, Working Paper No. 2020/159. https://www.imf.org/en/Publications/WP/Issues/2020/08/07/The-Effect-of-Containment-Measures-on-the-COVID-19-Pandemic-49572#:~:text=We%20examine%20this%20question%20using,is%20significant%20heterogeneity%20across%20countries. Accessed 6 Jun 2022.

37. Dreher N, Spiera Z, McAuley FM, Kuohn L, Durbin JR, Marayati NF, Ali M, Li AY, Hannah TC, Gometz A *et al*. Policy interventions, social distancing, and SARS-CoV-2 transmission in the United States: A retrospective state-level analysis. *Am J Med Sci* 2021, 361:575-584.

38. Duhon J, Bragazzi N, Kong JD. The impact of non-pharmaceutical interventions, demographic, social, and climatic factors on the initial growth rate of COVID-19: A cross-country study. *Sci Total Environ* 2021, 760:144325.

39. Ebrahim S, Ashworth H, Noah C, Kadambi A, Toumi A, Chhatwal J. Reduction of COVID-19 incidence and nonpharmacologic interventions: Analysis using a US county-level policy data set. *Journal of Medical Internet Research* 2020, **22**(12):e24614.

40. Esra R, Jamieson L, Fox MP, Letswalo D, Ngcobo N, Mngadi S, Estill J, Meyer-Rath G, Keiser O. Evaluating the impact of non-pharmaceutical interventions for SARS-CoV-2 on a global scale. *MedRxiv* 2020.

41. Fisher KA, Olson SM, Tenforde MW, Feldstein LR, Lindsell CJ, Shapiro NI, Files DC, Gibbs KW, Erickson HL, Prekker ME *et al*. Telework before illness onset among symptomatic adults aged >/=18 years with and without COVID-19 in 11 outpatient health care facilities - United States, July 2020. *MMWR Morb Mortal Wkly Rep* 2020, 69:1648-1653.

42. Gokmen Y, Baskici C, Ercil Y. Effects of non-pharmaceutical interventions against COVID-19: A cross-country analysis. *International Journal of Health Planning & Management* 2021, 05:05.

43. Guzzetta G, Riccardo F, Marziano V, Poletti P, Trentini F, Bella A, Andrianou X, Del Manso M, Fabiani M, Bellino S *et al*. Impact of a nationwide lockdown on SARS-CoV-2 transmissibility, Italy. *Emerging Infectious Diseases* 2021, 27:01.

44. Haug N, Geyrhofer L, Londei A, Dervic E, Desvars-Larrive A, Loreto V, Pinior B, Thurner S, Klimek P. Ranking the effectiveness of worldwide COVID-19 government interventions. *Nat Hum Behav* 2020, 4:1303-1312.

45. Herstein JJ, Degarege A, Stover D, Austin C, Schwedhelm MM, Lawler JV, Lowe JJ, Ramos AK, Donahue M. Characteristics of SARS-CoV-2 transmission among meat processing workers in Nebraska, USA, and effectiveness of risk mitigation measures. *Emerging Infectious Diseases* 2021, 27:1032-1038.

46. Islam N, Sharp SJ, Chowell G, Shabnam S, Kawachi I, Lacey B, Massaro JM, D'Agostino RB, Sr., White M. Physical distancing interventions and incidence of coronavirus disease 2019: natural experiment in 149 countries. *BMJ* 2020, 370:m2743.

47. Koh WC, Naing L, Wong J. Estimating the impact of physical distancing measures in containing COVID-19: an empirical analysis. *International Journal of Infectious Diseases* 2020, 100:42-49.

48. Lau H, Khosrawipour V, Kocbach P, Mikolajczyk A, Schubert J, Bania J, Khosrawipour T. The positive impact of lockdown in Wuhan on containing the COVID-19 outbreak in China. *Journal of Travel Medicine* 2021, 27:1-7.

49. Li Y, Campbell H, Kulkarni D, Harpur A, Nundy M, Wang X, Nair H, Usher Network for Covid-Evidence Reviews group. The temporal association of introducing and lifting non-pharmaceutical interventions with the time-varying reproduction number (R) of SARS-CoV-2: a modelling study across 131 countries. *The Lancet Infectious Diseases* 2021, 21:193-202.

50. Li Y, Li M, Rice M, Zhang H, Sha D, Li M, Su Y, Yang C. The impact of policy measures on human mobility, COVID-19 cases, and mortality in the US: a spatiotemporal perspective. *International Journal of Environmental Research and Public Health* 2021, 18:996.

51. Lin Z, Meissner CM. Health vs. wealth? Public health policies and the economy during Covid-19. In*.*: National Bureau of Economic Research, Inc, NBER Working Papers: 27099; 2020.

52. Liu Y, Morgenstern C, Kelly J, Lowe R, Cmmid Covid- Working G, Jit M. The impact of non-pharmaceutical interventions on SARS-CoV-2 transmission across 130 countries and territories. *BMC Medicine* 2021, 19:40.

53. Lyu W, Wehby GL. Comparison of estimated rates of coronavirus disease 2019 (COVID-19) in border counties in Iowa without a stay-at-home order and border counties in Illinois with a stay-at-home order. *JAMA network open* 2020, 3:e2011102-e2011102.

54. Lyu W, Wehby GL. Shelter-in-place orders reduced covid-19 mortality and reduced the rate of growth in hospitalizations. *Health Affairs* 2020, 39:1615-1623.

55. Padalabalanarayanan S, Hanumanthu VS, Sen B. Association of state stay-at-home orders and state-level African American population with COVID-19 case rates. *JAMA network open* 2020, 3:e2026010-e2026010.

56. Saez M, Tobias A, Varga D, Barcelo MA. Effectiveness of the measures to flatten the epidemic curve of COVID-19. The case of Spain. *Sci Total Environ* 2020, 727:138761.

57. Salvatore M, Basu D, Ray D, Kleinsasser M, Purkayastha S, Bhattacharyya R, Mukherjee B. Comprehensive public health evaluation of lockdown as a non-pharmaceutical intervention on COVID-19 spread in India: national trends masking state-level variations. *BMJ Open* 2020, 10:e041778.

58. Santamaria L, Hortal J. Chasing the ghost of infection past: identifying thresholds of change during the COVID-19 infection in Spain. *Epidemiology & Infection* 2020, 148:e282.

59. Santamaria L, Hortal J. COVID-19 effective reproduction number dropped during Spain's nationwide dropdown, then spiked at lower-incidence regions. *Science of the Total Environment* 2021, 751:142257.

60. Saul A, Scott N, Crabb BS, Majumdar SS, Coghlan B, Hellard ME. Impact of Victoria's Stage 3 lockdown on COVID-19 case numbers. *Medical Journal of Australia* 2020, 213:494-496.e491.

61. Schroder M, Bossert A, Kersting M, Aeffner S, Coetzee J, Timme M, Schluter J. COVID-19 in South Africa: outbreak despite interventions. *Scientific Reports* 2021, 11:4956.

62. Silva L, Figueiredo Filho D, Fernandes A. The effect of lockdown on the COVID-19 epidemic in Brazil: evidence from an interrupted time series design. *Cadernos de Saude Publica* 2020, 36:e00213920.

63. Singh BB, Lowerison M, Lewinson RT, Vallerand IA, Deardon R, Gill JPS, Singh B, Barkema HW. Public health interventions slowed but did not halt the spread of COVID-19 in India. *Transboundary & Emerging Diseases* 2020, 04:04.

64. Singh S, Shaikh M, Hauck K, Miraldo M. Impacts of introducing and lifting nonpharmaceutical interventions on COVID-19 daily growth rate and compliance in the United States. *Proceedings of the National Academy of Sciences of the United States of America* 2021, 118:23.

65. Thayer WM, Hasan MZ, Sankhla P, Gupta S. An interrupted time series analysis of the lockdown policies in India: a national-level analysis of COVID-19 incidence. *Health Policy & Planning* 2021, 26:26.

66. Tobias A. Evaluation of the lockdowns for the SARS-CoV-2 epidemic in Italy and Spain after one month follow up. *Science of the Total Environment* 2020, 725:138539.

67. White ER, Hebert-Dufresne L. State-level variation of initial COVID-19 dynamics in the United States. *PLoS ONE [Electronic Resource]* 2020, 15:e0240648.

68. Wong CKH, Wong JYH, Tang EHM, Au CH, Lau KTK, Wai AKC. Impact of national containment measures on decelerating the increase in daily new cases of COVID-19 in 54 countries and 4 epicenters of the Pandemic: Comparative observational study. *Journal of Medical Internet Research* 2020, 22:e19904.

69. Xu J, Hussain S, Lu G, Zheng K, Wei S, Bao W, Zhang L. Associations of stay-at-home order and face-masking recommendation with trends in daily new cases and deaths of laboratory-confirmed COVID-19 in the United States. *Exploratory research and hypothesis in medicine* 2020;5(3):77-86.

70. Zhang X, Warner ME. COVID-19 Policy Differences across US states: Shutdowns, reopening, and mask mandates. *International Journal of Environmental Research & Public Health [Electronic Resource]* 2020, 17:18.

71. Badellino H, Gobbo ME, Torres E, Aschieri ME, Biotti M, Alvarez V, Gigante C, Cachiarelli M. 'It's the economy, stupid': Lessons of a longitudinal study of depression in Argentina. *International Journal of Social Psychiatry* 2022;68(2):384-91.

72. Barone Gibbs B, Kline CE, Huber KA, Paley JL, Perera S. Covid-19 shelter-at-home and work, lifestyle and well-being in desk workers. *Occupational Medicine (Oxford)* 2021, 71:86-94.

73. Canet-Juric L, Andres ML, Del Valle M, Lopez-Morales H, Poo F, Galli JI, Yerro M, Urquijo S. A longitudinal study on the emotional impact cause by the COVID-19 Pandemic quarantine on general population. *Frontiers in Psychology* 2020, 11:565688.

74. Castellini G, Rossi E, Cassioli E, Sanfilippo G, Innocenti M, Gironi V, Silvestri C, Voller F, Ricca V. A longitudinal observation of general psychopathology before the COVID-19 outbreak and during lockdown in Italy. *Journal of Psychosomatic Research* 2021, 141:110328.

75. Cecchini JA, Carriedo A, Fernandez-Rio J, Mendez-Gimenez A, Gonzalez C, Sanchez-Martinez B, Rodriguez-Gonzalez P. A longitudinal study on depressive symptoms and physical activity during the Spanish lockdown. *International Journal of Clinical & Health Psychology* 2021, 21:100200.

76. Fancourt D, Steptoe A, Bu F. Trajectories of anxiety and depressive symptoms during enforced isolation due to COVID-19 in England: a longitudinal observational study. *The Lancet Psychiatry* 2021, 8:141-149.

77. Gonzalez-Sanguino C, Ausin B, Castellanos MA, Saiz J, Munoz M. Mental health consequences of the Covid-19 outbreak in Spain. A longitudinal study of the alarm situation and return to the new normality. *Prog Neuropsychopharmacol Biol Psychiatry* 2021, 107:110219.

78. Gopal A, Sharma AJ, Subramanyam MA. Dynamics of psychological responses to COVID-19 in India: A longitudinal study. *PLoS ONE [Electronic Resource]* 2020, 15:e0240650.

79. Hyland P, Shevlin M, Murphy J, McBride O, Fox R, Bondjers K, Karatzias T, Bentall RP, Martinez A, Vallieres F. A longitudinal assessment of depression and anxiety in the Republic of Ireland before and during the COVID-19 pandemic. *Psychiatry Research* 2021, 300:113905.

80. Le K, Nguyen M. The psychological consequences of COVID-19 lockdowns. *International Review of Applied Economics* 2021, 35:147-163.

81. Mergel E, Schutzwohl M. A longitudinal study on the COVID-19 pandemic and its divergent effects on social participation and mental health across different study groups with and without mental disorders. *Social Psychiatry & Psychiatric Epidemiology* 2021, 10:10.

82. O'Connor RC, Wetherall K, Cleare S, McClelland H, Melson AJ, Niedzwiedz CL, O'Carroll RE, O'Connor DB, Platt S, Scowcroft E *et al*. Mental health and well-being during the COVID-19 pandemic: longitudinal analyses of adults in the UK COVID-19 Mental Health & Wellbeing study. *British Journal of Psychiatry* 2021:218:326-33.

83. Ozamiz-Etxebarria N, Idoiaga Mondragon N, Dosil Santamaria M, Picaza Gorrotxategi M. Psychological symptoms during the two stages of lockdown in response to the COVID-19 outbreak: An investigation in a sample of citizens in northern Spain. *Frontiers in Psychology* 2020, 11:1491.

84. Roma P, Monaro M, Colasanti M, Ricci E, Biondi S, Di Domenico A, Verrocchio MC, Napoli C, Ferracuti S, Mazza C. A 2-month follow-up study of psychological distress among Italian people during the COVID-19 lockdown. *International Journal of Environmental Research & Public Health [Electronic Resource]* 2020, 17:05.

85. Somma A, Krueger RF, Markon KE, Gialdi G, Colanino M, Ferlito D, Liotta C, Frau C, Fossati A. A longitudinal study on clinically relevant self-reported depression, anxiety and acute stress features among Italian community-dwelling adults during the COVID-19 related lockdown: Evidence of a predictive role for baseline dysfunctional personality dimensions. *Journal of Affective Disorders* 2021, 282:364-371.

86. Beland L-P, Brodeur A, Wright T. COVID-19, stay-at-home orders and employment: Evidence from CPS data. In*.*: Carleton University, Department of Economics, Carleton Economic Papers: 20-04; 2020: 111 pages.

87. Churchill B. COVID-19 and the immediate impact on young people and employment in Australia: A gendered analysis. *Gender, Work & Organization* 2020, 31:31.

88. Coibion O, Gorodnichenko Y, Weber M. The cost of the Covid-19 crisis: Lockdowns, macroeconomic expectations, and consumer spending. In*.*: National Bureau of Economic Research, Inc, NBER Working Papers: 27141; 2020.

89. Robinson E, Daly M. Explaining the rise and fall of psychological distress during the COVID-19 crisis in the United States: Longitudinal evidence from the Understanding America Study. *British Journal of Health Psychology* 2021, 26:570-587.
